# Synthesis and Structural Characterization of Amidine, Amide, Urea and Isocyanate Derivatives of the Amino-*closo*-dodecaborate Anion [B_12_H_11_NH_3_]^−^

**DOI:** 10.3390/molecules23123137

**Published:** 2018-11-29

**Authors:** Yuanbin Zhang, Yuji Sun, Tao Wang, Jiyong Liu, Bernhard Spingler, Simon Duttwyler

**Affiliations:** 1 Department of Chemistry, Zhejiang University, 38 Zheda Road, Hangzhou 310027, China; 15868145065@126.com (Y.Z.); sunyuji1991@163.com (Y.S.); 21637046@zju.edu.cn (T.W.); liujiyong1205@163.com (J.L.); 2 Key Laboratory of Biomass Chemical Engineering of Ministry of Education, Department of Chemical and Biological Engineering, Zhejiang University, 38 Zheda Road, Hangzhou 310027, China; 3 Department of Chemistry, University of Zurich, Winterthurerstrasse 190, 8057 Zurich, Switzerland; spingler@chem.uzh.ch

**Keywords:** dodecaborate, boron cluster, borane, amidine, amide, urea, isocyanate

## Abstract

The synthesis and structural characterization of new derivatives of [B_12_H_12_]^2−^ is of fundamental interest and is expected to allow for extended applications. Herein we report on the synthesis of a series of amidine, amide, urea and isocyanate derivatives based on the amino-*closo*-dodecaborate anion [B_12_H_11_NH_3_]^−^. Their structures have been confirmed by spectroscopic methods, and nine crystal structures are presented.

## 1. Introduction

**Figure 1:**
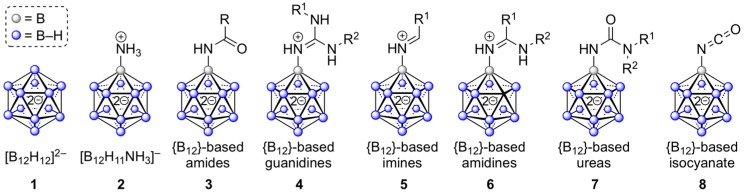
General structures of the parent *closo*-dodecaborate dianion [B_12_H_12_]^2−^ and its derivatives.

The *closo*-dodecaborate dianion [B_12_H_12_]^2−^ (**1**, Figure 1) is an icosahedral boron cluster with 12 identical B–H vertices, and it possesses unique properties such as spherical electron delocalization and high thermal/chemical stability [1,2,3,4]. It is considered a 3D analogue of benzene, and applications of [B_12_H_12_]^2−^ as well as its derivatives have been found in many fields, such as weakly coordinating anions [5,6,7,8,9,10,11,12,13,14], medicinal chemistry [15,16,17], catalysis [18,19], ligand design [20,21,22] and supramolecular chemistry [23,24,25,26,27].

Since the isolation of **1** in 1960 [28], many routes to substituted *closo*-dodecaborate anions have been reported *via* the construction of B–N, B–O, B–S, B–Hal or B–C bonds [29], and the ammonium dodecaborate [B_12_H_11_NH_3_]^−^ (**2**) serves as one of the fundamental building blocks for further functionalization [30]. Monoanionic **2** can be synthesized from the reaction of **1** with hydroxylamine-*O*-sulfonic acid (H_2_N-SO_3_H) on a multi-gram scale [30,31,32]. The –NH_3_ site can be deprotonated under basic conditions and then combined with acyl chlorides, carbodiimides or aldehydes to afford the corresponding amides **3** [33,34,35,36,37], guanidines **4** [34] and imines **5** [38]. Dodecaborate amidines **6** have not been explored yet, and urea derivatives **7** were unknown until we recently found that the reaction of **2** with dialkylcarbamoyl chlorides ClC(O)NMe_2_ or ClC(O)NEt_2_ affords the corresponding {B_12_}-substituted *N*,*N*-dialkyl ureas [39]. The isocyanate derivative **8** was originally synthesized from the reaction of the carbonyl derivative [B_12_H_11_(CO)]^−^ with NaN_3_ but characterized only by ^11^B-NMR and IR spectroscopy [40]. Herein we present: (1) the synthesis of three new {B_12_}-based amidines **6** with two crystal structures; (2) six crystal structures of {B_12_}-based amides **3** and a simple approach for the interconversion between their dianionic and monoanionic forms; (3) the synthesis of two new {B_12_}-based aromatic ureas **7**; (4) a new route to isocyanate **8** and its crystal structure.

## 2. Results and Discussion

### 2.1. Synthesis of {B_12_}-based Amidinium Ions

**Scheme 1:**
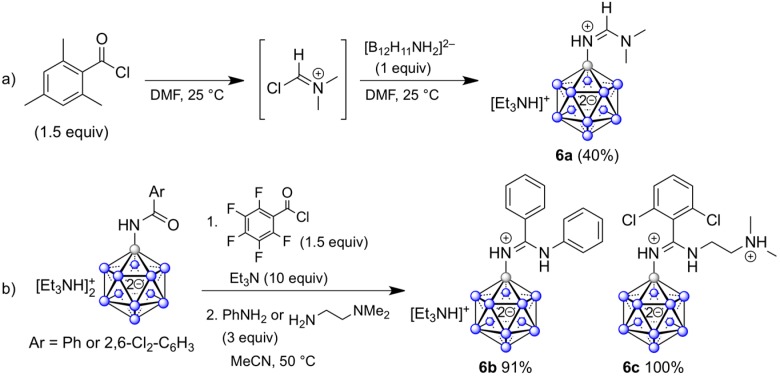
Synthesis of {B_12_}-based amidinium ions **6a**–**c** using (**a**) the chloroiminium intermediate derived from dimethylformamide and (**b**) pentafluorophenylbenzoyl chloride as activating agent.

Amidine derivatives based on the {B_12_} cluster have not been reported before, and in the following, methods to synthesize them are presented. Combination of dimethyl formamide with 2,4,6-trimethylbenzoyl chloride afforded the chloroiminium intermediate, which upon attack by [B_12_H_11_NH_2_]^2−^ afforded **6a** (Scheme 1a). This reaction allowed for the isolation of the desired product; however, the yield of 40% was moderate, and furthermore extension of the substrate scope by this method did not appear convenient. Using a related strategy, we found that the carbonyl group of {B_12_}-based amides can be activated by pentafluorobenzoyl chloride, and subsequent attack by amines would then lead to {B_12_}-based amidinium ions (Scheme 1b). Following this approach, products **6b** and **6c** were isolated in excellent yields of 91% and 100%, respectively.

**Figure 2:**
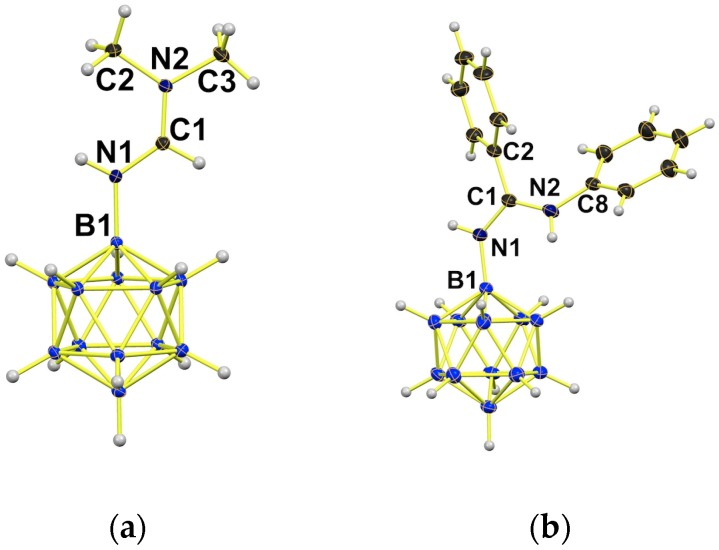
Crystal structures of (**a**) **6a** and (**b**) **6b**; cations and solvent molecules are omitted for clarity, and thermal ellipsoids are displayed at the 30% level.

Single crystals of **6a** and **6c** were obtained from acetonitrile solutions, and ORTEP representations of **6a** and **6c** are displayed in Figure 2. Observed distances (Å) are B1–N1 1.520(2), N1–C1 1.3078(18), C1–N2 1.3092(19), N2–C2 1.451(2) and N2–C3 1.4662(19) for **6a**; B1–N1 1.534(4), N1–C1 1.312(4), C1–N2 1.331(4), C1–C2 1.485(4) and N2–C8 1.420(4) for **6b**. These structural features are similar to those of typical organic amidinium ions; in addition, the coordination geometry around C1 is perfectly trigonal-planar for both products with a sum of angles of 360°. The torsion angles N1-C1-N2-C2 and N1-C1-N2-C3 of **6a** are 0.0(2) and –175.35(15)°, respectively, indicating coplanarity of the amidine and the dimethylamino moieties. On the other hand, **6b** is more twisted with a torsion angle N1-C1-N2-C8 of 154.4(3)°.

### 2.2. Synthesis of {B_12_}-based Amides

**Scheme 2:**
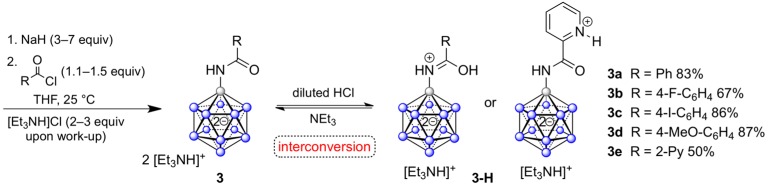
Synthesis of {B_12_}-based amides **3** and interconversion between their dianionic and monoanionic forms; compounds **3a**–**d** were prepared according to [37].

{B_12_}-based amides **3** were originally synthesized by other groups [33,34,35,36]. The products were isolated in their *O*-protonated form **3-H**, and there was only one crystal structure reported, namely **3a-H** with R = Ph (Scheme 2). Our group recently modified the reaction conditions, and the products were obtained in up to 95% yields in the *O*-deprotonated form **3** after chromatography on silica gel [36]. Our approach uses smaller amounts of acyl chlorides and a slight excess of sodium hydride base, which results in the isolation of the dianionic form (for this study, **3a**–**d** were resynthesized according to [37] in order to probe their solid-state structures). Adjustment of the pH value with diluted hydrochloric acid during the work-up or after isolation leads to **3-H** in their *O*-protonated form. Back-conversion to **3** can be achieved by dissolution in MeCN, treatment with Et_3_N and distillation of all the volatiles. Pyridine-substituted **3e** was synthesized as a new product. Since purification by chromatography proved difficult, it was isolated as **3e-H** in 50% yield upon acidic work-up and recrystallization from methanol. Conversion to **3e** occurred quantitatively using the above-mentioned procedure. In contrast to previously described amides, protonation of **3e** takes place at the pyridine ring, indicating the higher basicity of the heterocycle as compared to the amide moiety. The ^11^B{^1^H}-NMR spectra of **3b**, **3b-H**, **3e** and **3e-H** are shown in Figure S1 as representative examples to demonstrate the effect of protonation. For R = Ph (**3a**), 4-F-C_6_H_4_ (**3b**), 4-I-C_6_H_4_ (**3c**) 4-MeO-C_6_H_4_ (**3d-H**), 2-pyridyl (**3e**) and 2-pyridyl-H (**3e-H**), crystal structures were elucidated, and selected structural features are discussed below.

**Figure 3:**
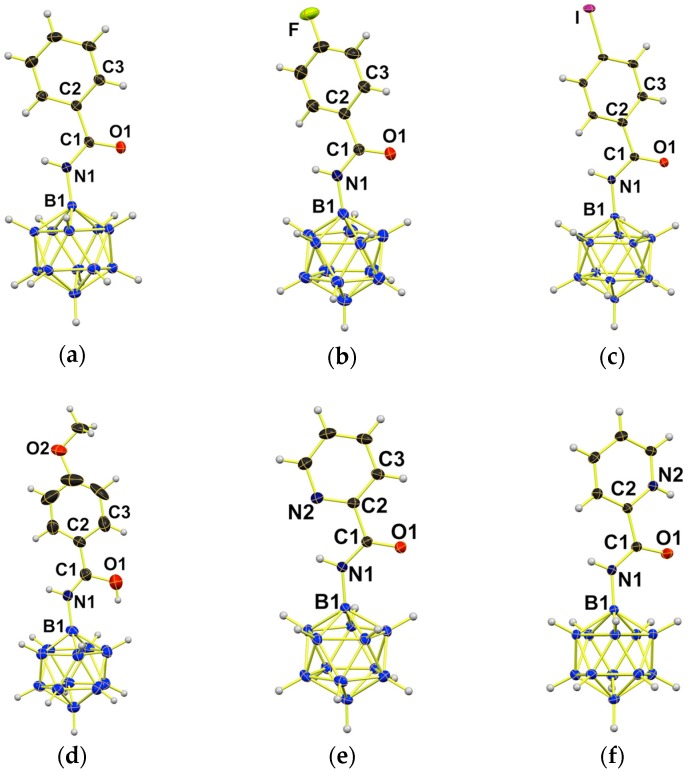
Crystal structures of (**a**) **3a**, (**b**) **3b**, (**c**) **3c**, (**d**) **3d-H**, (**e**) **3e** and (**f**) **3e-H**; cations and solvent molecules are omitted for clarity, and thermal ellipsoids are displayed at the 30% level.

Single crystals of **3a**, **3b**, **3c, 3d-h**, **3e** and **3e-H** were obtained from acetonitrile, acetone, acetonitrile-acetone or acetonitrile-methanol solutions. ORTEP representations are displayed in Figure 3, and a summary of structural parameters is given in Table 1, including data for *O*-protonated **3a-H**, originally reported by Gabel and coworkers [34]. The seven compounds can be grouped into the series **3a**/**3b**/**3c/3e**/**3e-H** and **3a-H**/**3d-H**. For the former series, distances (Å) fall within B1–N1 1.51–1.52, N1–C1 1.31–1.33 and C1–O1 1.23–1.25. These compounds thus exhibit strong structural resemblence to classical organic amides. For the latter pair, observed ranges (Å) are B1–N1 1.53–1.58, N1–C1 1.26–1.30 and C1–O1 1.31–1.34. These values are consistent with O-protonation, leading to a more pronounced allyl cation-type equalization of bond lengths. On the other hand, all seven products share two features: The central carbon atom C1 has perfect trigonal-planar geometry with a sum of angles of 360°. Furthermore, the torsion angles O1-C1-C2-C3 (O1-C1-C2-N2 for **3e-H**) fall in the range of –18° to +32° and indicate a certain degree of conjugation between the aromatic rings and the N1-C1-O1 π system.

### 2.3. Synthesis of {B_12_}-based Ureas

**Scheme 3:**
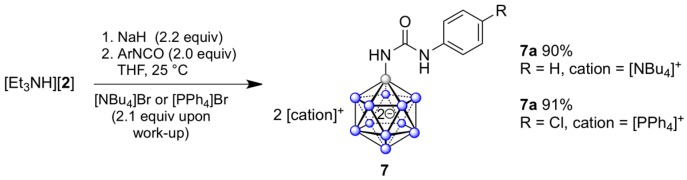
Synthesis of {B_12_}-based ureas **7**.

{B_12_}-based ureas with *N*{B_12_},*N*′(aryl) substitution have not been reported before. The synthetic strategy to prepare dodecaboranyl *N*,*N*-dialkyl ureas recently reported by our group involved the combination of **2** with dialkylcarbamoyl chlorides [39]. We wondered whether aromatic isocyanates could be used instead of carbamoyl chlorides to achieve the new substitution pattern, given the commercial availability of many ArNCO reagents. We found that the reaction of **2** with aromatic isocyanates under basic conditions directly leads to the formation of the corresponding urea derivatives of the structure {B_12_}NH-C(O)-NHAr. Thus, the transformations with commercially available PhNCO and 4-Cl-C_6_H_4_NCO cleanly gave products **7a** and **7b** in yields of 90% and 91% (Scheme 3). They were isolated by precipitation upon cation exchange to [NBu_4_]^+^ or [PPh_4_]^+^ and characterized by NMR spectroscopy and mass spectrometry.

### 2.4. Synthesis of Dodecaboranyl Isocyanate

**Scheme 4:**
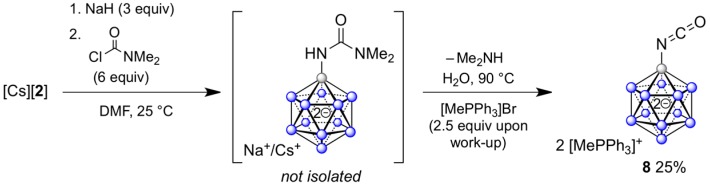
Synthesis of isocyanate **8**.

Isocyanates are important intermediates in organic synthesis that are used in the manufacture of, e.g., agrochemicals and polyurethanes [41]. Only one publication by Alam and coworkers from 1989 mentioned the isolation of dodecaboranyl isocyanate **8** [40]. It was prepared *via* the reaction of [B_12_H_11_(CO)]^−^ with NaN_3_ and analyzed by ^11^B-NMR and IR spectroscopy. However, further characterization was not given, and in particular the crystal structure was not reported. Because the isocyanate moiety serves as versatile functional group handle capable of providing access to a number of novel {B_12_}-based derivatives, we were interested in resynthesizing **8**. However, multiple attempts to reproduce the original procedure were not successful in our laboratory, and we therefore sought to establish an alternative route. Since dodecaboranyl *N*,*N*-dialkyl ureas can be prepared easily [39], their thermal fragmentation appeared as an attractive strategy. Indeed, treatment of **2** with base and ClC(O)NMe_2_ to give the intermediate urea, followed by heating in water, afforded **8** in 25% overall yield (Scheme 4). The yield of this sequence is rather low, and efforts to improve the protocol are ongoing.

**Figure 4:**
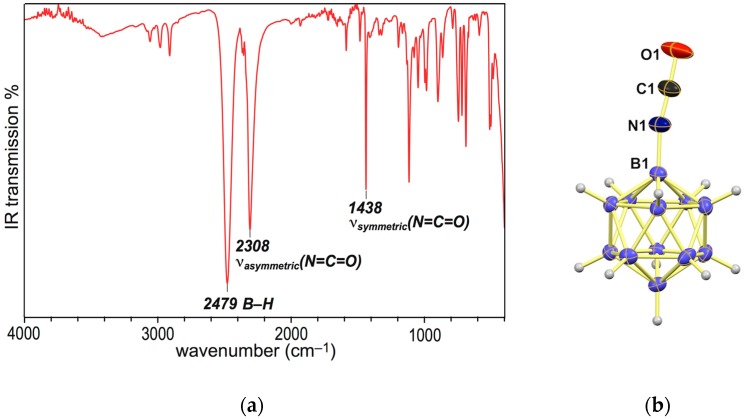
(**a**) IR spectrum and (**b**) crystal structure of [MePPh_3_]_2_[B_12_H_11_NCO]; cations in the crystal structure omitted for clarity, thermal ellipsoids displayed at the 30 % level.

In acetonitrile-*d*_3_ solution, the ^11^B NMR shifts of **8** were −7.7, −15.4, −16.7 and −19.3 ppm, while ^1^H NMR resonances appeared at 1.23, 0.97 and 0.75 ppm. The NCO ^13^C NMR signal could not be detected unambiguously; it is known from organic isocyanates that this signal can be very broad and difficult to observe. Interestingly, **8** is inert towards air and moisture. Solutions in acetone, kept under ambient conditions, remained unchanged over six months. The IR spectrum showed characteristic absorbtions at 2479 cm^−1^, 2308 cm^−1^ and 1438 cm^−1^ stemming from B–H, ν_asymmetric_(NCO) and ν_symmetric_(NCO) stretchings, respectively (Figure 4a) [42].

Colorless single crystals of **8** were obtained from acetone solution. X-Ray diffraction revealed distances (Å) of B1–N1 1.499(3), N1–C1 1.138(3) and C1-O1 1.186(3), clearly indicating the two double bonds of the N=C=O moiety (Figure 4b). This finding is in agreement with the angle N1-C1-O1 of 176.5(3)°, showing almost linear geometry of the isocyanate group. The B1-N1-C1 angle of 163.1(3)° is also quite large, while no unusual structural features were observed for the {B_12_} cluster.

## 3. Materials and Methods

### 3.1. General

If not otherwise specified, reagents and organic solvents were commercially available and used without further purification. Anhydrous solvents were prepared by passage through activated Al_2_O_3_ and stored over 3 Å molecular sieves. CD_3_CN and CD_2_Cl_2_ were purchased from Cambridge Isotope Laboratories and filtered through Al_2_O_3_ prior to use. [B_12_H_12_]^2−^ and [B_12_H_11_NH_3_]^−^ salts and dodecaborate amides **3a**–**e** were prepared according to the literature [10,36].

Glassware for air-sensitive reations was dried at 150 °C and allowed to cool in a vacuum. Reactions carried out in a glovebox were run under a nitrogen atmosphere with O_2_, H_2_O < 1 ppm.

Thin-layer chromatography (TLC) was carried out using silica gel 60, F254 with a thickness of 0.25 mm. Column chromatography was performed on silica gel 60 (200–300 mesh).

Low-resolution ESI-MS data were recorded on Advion Expression CMS instrument (Advion, Ithaca, NY, USA). High-resolution MS data were recorded using IT-TOF detection (Shimadzu, Kyoto, Japan) equipped with an electrospray ionization source (ESI). Accurate mass determination was corrected by calibration using sodium trifluoroacetate clusters as a reference.

Single-crystal X-ray diffraction studies were performed on an Oxford Diffraction Gemini A Ultra diffractometer (Agilent Technologies, Santa Clara, CA, USA) equipped with an 135 mm Atlas CCD detector and using Mo K-a radiation.

NMR spectra were recorded on a Bruker AVANCE III 500 spectrometer (^1^H NMR 500.13 MHz, ^13^C NMR 125.77 MHz, ^11^B NMR 160.46 MHz) or a Bruker AVANCE III 400 spectrometer (Bruker, Billerica, MA, USA) (^1^H NMR 400.13 MHz, ^13^C NMR 100.62 MHz, ^11^B NMR 128.38 MHz) at the temperature indicated. Data are reported as follows: Chemical shift in ppm, multiplicity (s = singlet, d = doublet, t = triplet, q = quartet, m = multiplet, dd = doublet of doublets, etc.), coupling constant *J* in Hz, integration, and (where applicable) interpretation. Signals were referenced against solvent peaks (^1^H: residual CHD_2_C(O)CD_3_ = 2.05 ppm, residual CHD_2_CN = 1.94 ppm, residual CHDCl_2_ = 5.32 ppm, ^13^C{^1^H}: *C*D_3_C(O)*C*D_3_ = 29.84 ppm, *C*D_3_CN = 1.32 ppm, CD_2_Cl_2_ = 53.32 ppm). ^11^B and ^11^B{^1^H} NMR spectra were calibrated against external BF_3_*Et_2_O = 0 ppm (BF_3_*Et_2_O capillary in C_6_D_6_).

### 3.2. Experimental Section

*Synthesis of [Et*_3_*NH]*(**3e-H**): In a glovebox filled with N_2_, a 20 mL vial was charged with [Et_3_NH][B_12_H_11_NH_3_] (212.4 mg, 0.817 mmol, 1 equiv), NaH (138.2 mg, 5.758 mmol, 7 equiv) and a stir bar. THF (4 mL) and DMF (4 mL) were added, and the mixture was stirred at room temperature for 10 min until there was no H_2_ evolution anymore. Then pyridine-2-carbonyl chloride hydrochloride PyCOCl·HCl (220.2 mg, 1.237 mmol, 1.5 equiv) was slowly added. The conversion was complete after stirring for 5 h. The flask was transferred out of the glovebox. H_2_O (4 mL) was added, and the pH value of the reaction mixture was adjusted to 2–3 with 1 M aqueous HCl. [NEt_3_H]Cl (300 mg, 2.180 mmol, 2.7 equiv) was added, and the reaction mixture was extracted with MeCN/EtOAc (1:2 *v*/*v*). The organic layers were concentrated on a rotary evaporator. The residue was purified by recrystallization from methanol to afford yellowish crystals of [Et_3_NH][**3e-H**] (150 mg, 50%). ^1^H{^11^B} NMR (400 MHz, CD_3_CN): δ = 8.96 (s, 1H, anionic NH), 8.90–8.86 (m, 1H, Py H), 8.18–8.14 (overlapping m, 2H, Py H), 7.89–7.72 (m, 1H, Py H), 6.63 (t, 1H, *J*_NH_ = 52 Hz, NH), 3.27 (s, 1H, NH), 3.20–3.15 (m, 6H, cationic N–C*H*_2_), 1.47 (broad signal, 5H, B–H), 1.24 (t, *J* = 7.4 Hz, 9H, cationic C*H*_3_), 1.20 (broad signal, 5H, B–H), 1.13 (broad signal, 1H, B–H). ^13^C{^1^H} NMR (101 MHz, CD_3_CN): δ = 166.7, 149.5, 143.9, 141.5, 129.8, 124.5 (6 anionic signals), 48.0, 9.2 (2 cationic signals). ^11^B{^1^H} NMR (128 MHz, CD_3_CN): δ = –7.6 (1B, B–N), –15.3 (5B, B–H), –15.7 (overlapping signals, 6B, B–H). High-resolution ESI-MS (negative mode, MeOH): *m*/*z* calcd for [C_6_H_17_B_12_N_2_O]^−^ 263.2430. Found: 263.2459.

*Transformation of [Et*_3_*NH]*(**3e-H**) *to [Et*_3_*NH]*_2_(**3e**): A 20 mL vial was charged with [Et_3_NH][**3e-H**] (50 mg) and a stir bar. MeCN (3 mL) and Et_3_N (0.5 mL) were added, and the solution was stirred at room temperature for 1 h. Then the stir bar was removed, and the solution was concentrated on a rotary evaporator and dried overnight under vacuum at 80 °C to afford compound [Et_3_NH]_2_[**3e**] in quantitative yield. This method can also be applied for the transformation of other compounds **3-H** to **3** quantitatively. ^11^B{^1^H} NMR spectra of **3b**, **3b-H**, **3e** and **3e-H** are displayed in Figure S1. ^1^H{^11^B} NMR (400 MHz, CD_3_CN): δ = 8.56 (broad signal, 1H, Py H), 8.09–8.00 (m, 1H, Py H), 7.99–7.80 (overlapping m, 2H, Py H and amide N–H), 7.50–7.38 (m, 1H, Py H), 4.63 (broad t, 2H, *J*_NH_ = 52 Hz, N–H from cation), 3.25–3.01 (m, 12H, cationic N–CH_2_), 1.34 (s, 5H, B–H), 1.24 (t, *J* = 7.4 Hz, 9H, cationic CH_3_), 1.03 (broad signal, 5H, B–H), 0.89 (broad signal, 1H, B–H). ^13^C{^1^H} NMR (101 MHz, CD_3_CN): δ = 166.2, 152.9, 149.0, 138.5, 126.4, 122.2 (6 anionic signals), 47.8, 9.1 (2 cationic signals). ^11^B{^1^H} NMR (128 MHz, CD_3_CN): δ = –5.3 (1B, B–N), –15.3 (5B, B–H), –16.4 (5B, B–H), -18.7 (1B, B–H). High-resolution ESI-MS (negative mode, MeOH): *m*/*z* calcd for [C_6_H_17_B_12_N_2_O]^2−^ 131.1226. Found: 131.1254.

*Synthesis of amidine [Et*_3_*NH]*(**6a**): In a glovebox, a dry 20 mL vial, equipped with a stir bar, was charged with [Et_3_NH][B_12_H_11_NH_3_] (102 mg, 0.40 mmol, 1 equiv). Then anhydrous DMF (1 mL) was added. The vial was transferred to a fumehood, and dry Et_3_N (1.0 mL, 7.20 mmol, 18 equiv) was added to the solution under N_2_ protection. Then 2,4,6-trimethylphenylcarboxylic acid chloride (110 mg, 0.60 mmol, 1.5 equiv) was added. The mixture was stirred at 25 °C for 4 h. The reaction was quenched with an aqueous [Et_3_NH]Cl solution (2 mL H_2_O + 2 equiv [Et_3_NH]Cl); the pH value at this point was ca. 7–8. The mixture was extracted with DCM/MeCN = 4: 1 (8 × 10 mL). The combined organic layers were dried over MgSO_4_, and the solution was filtered and concentrated by rotary evaporation. The cloudy residue was purified by silica gel column chromatography (eluent DCM/MeCN = 10:3, fraction size 20 mL). The combined eluates were concentrated on a rotary evaporator and dried under vacuum at 60 °C overnight to afford compound [Et_3_NH][**6a**] as a colorless solid (50.4 mg, 40%). ^1^H{^11^B} NMR (400 MHz, CD_3_CN, 23 °C): δ 7.76 (d, *J* = 16.0 Hz, 1H, N=CH–N), 6.41 (broad signal, 1H, N–H), 3.13 (q, *J* = 7.2 Hz, 6H, cationic N–CH_2_), 3.08 (s, 3H, anionic N–CH_3_), 2.83 (s, 3H, anionic N–CH_3_), 1.26 (broad signal, 5H, B–H), 1.24 (t, *J* = 7.2 Hz, 9H, cationic N–CH_2_C*H*_3_), 1.03 (broad signal, 5H, B–H), 0.85 (broad signal, 1H, B–H). ^13^C{^1^H} NMR (100 MHz, CD_3_CN, 23 °C): δ 157.3 (N=C–N), 48.0 (cationic CH_2_), 43.1, 35.7 (two N–C signals), 9.2 (cationic CH_3_). ^11^B{^1^H} NMR (160 MHz, CD_3_CN, 23 °C): δ −4.2 (1B, B–N), −14.5 to −17.0 (10B, B–H), −19.0 (1B, B–H). High-resolution ESI-MS (negative mode, MeOH): *m*/*z* calcd for [C_3_H_19_B_12_N_2_]^−^: 213.2738. Found: 213.2762.

*Synthesis of amidine [Et*_3_*NH]*(**6b**): A dry 20 mL vial, equipped with a stir bar, was charged with [Et_3_NH]_2_[B_12_H_11_NHCOC_6_H_5_] (101 mg, 0.22 mmol, 1 equiv). Then anhydrous MeCN (3 mL) was added, and dry Et_3_N (0.3 mL, 2.16 mmol, 9.8 equiv) was added to the solution under N_2_ protection. Pentafluorophenylcarboxylic acid chloride (80.0 mg, 0.35 mmol, 1.5 equiv) was added at 25 °C. The temperature was raised to 50 °C. After 30 min, aniline (61 mg, 0.66 mmol, 3.0 equiv) was added. The mixture was stirred for another 4 h and concentrated by rotary evaporation. The cloudy residue was purified by silica gel column chromatography (eluent DCM/MeCN = 4:1, fraction size 20 mL). The combined eluates were concentrated on a rotary evaporator and dried under vacuum at 60 °C overnight to afford compound [Et_3_NH][**6b**] as a yellow solid (87.7 mg, 91%). ^1^H{^11^B} NMR (400 MHz, CD_2_Cl_2_, 23 °C): δ 10.00 (s, 1H, N–H), 7.53–7.48 (m, 1H, phenyl H), 7.41–7.35 (overlapping m, 4H, phenyl H), 7.24-7.09 (overlapping m, 3H, phenyl H), 7.03–6.78 (overlapping broad signal and m, 3H, phenyl H and N–H), 6.65 (broad signal, 1H, N–H) 3.29–3.22 (m, 6H, cationic N–CH_2_), 1.62 (broad signal, 5H, B–H), 1.40 (t, *J* = 7.2 Hz, 9H, cationic N–CH_2_C*H*_3_), 1.22 (broad signal, 5H, B–H), 1.05 (broad signal, 1H, B–H). This spectrum contained small signals at 7.18, 6.71 and 6.67 ppm ascribed to residual aniline. ^13^C{^1^H} NMR (100 MHz, CD_3_CN, 23 °C): δ 165.6 (N=C–N), 138.1, 133.2, 131.3, 130.4, 130.1, 129.9, 127.5, 125.6 (8 aryl signals), 48.3 (cationic N–CH_2_), 9.4 (cationic N–CH_3_). This spectrum showed small signals at 149.1, 130.2, 118.3 and 115.6 ppm ascribed to residual aniline. ^11^B{^1^H} NMR (128 MHz, CD_2_Cl_2_, 23 °C): δ −5.8 (1B, B–N), −13.5 to −16.5 (10B, B–H), −17.4 (1B, B–H). High-resolution ESI-MS (negative mode, MeOH): *m*/*z* calcd for [C_13_H_23_B_12_N_2_]^−^: 337.3056. Found: 337.2382.

*Synthesis of amidine [Et*_3_*NH]*(**6c**): A dry 20 mL vial, equipped with a stir bar, was charged with [Et_3_NH]_2_[B_12_H_11_NHCOC_6_H_3_Cl_2_] (177 mg, 0.33 mmol, 1 equiv). Then anhydrous MeCN (3 mL) was added, and dry Et_3_N (0.45 mL, 3.25 mmol, 9.8 equiv) was added to the solution under N_2_ protection. Pentafluorophenylcarboxylic acid chloride (128 mg, 0.55 mmol, 1.7 equiv) was added at 25 °C. The temperature was raised to 50 °C. After 30 min, *N,N*-dimethylethylamine (88 mg, 1.00 mmol, 3.0 equiv) was added. The mixture was stirred for another 4 h, and 1 M aqueous HCl (5 mL) was added. The suspension was extracted with EtOAc/MeCN 3:1 (5 × 10 mL). The combined organic layers were dried over MgSO_4_, and the solution was filtered and concentrated by rotary evaporation. The cloudy residue was purified by silica gel column chromatography (eluent DCM/MeCN = 4:3, fraction size 20 mL). The combined eluates were concentrated and dried under vacuum at 60 °C overnight to afford compound [Et_3_NH][**6c**] as a yellow solid (132 mg, 100%). ^1^H{^11^B} NMR (400 MHz, CD_3_CN, 23 °C): δ 8.54 (broad signal, 1H, N–H), 7.59–7.55 (overlapping m, 3H, aryl H), 7.46 (broad signal, 1H, N–H), 6.98 (very broad signal, 1H, N–H), 3.43 (dt, *J* = 7.2 Hz, 7.2 Hz, 2H, CH_2_), 3.24 (t, *J* = 7.2 Hz, 2H, CH_2_), 2.77 (s, 6H, N–CH_3_), 1.41 (broad signal, 5H, B–H), 1.12 (broad signal, 5H, B–H), 1.06 (broad signal, 1H, B–H). ^13^C{^1^H} NMR (100 MHz, CD_3_CN, 23 °C): δ 161.9 (N=C–N), 134.6, 134.2, 129.8, 129.2 (4 aryl signals), 56.9, 44.8, 40.0. ^11^B{^1^H} NMR (128 MHz, CD_3_CN, 23 °C): δ −6.9 (1B, B–N), −13.0 to −18.0 (overlapping signals with peaks at −15.2 and −16.1 ppm, 11B, B–H). High-resolution ESI-MS (negative mode, MeOH): *m*/*z* calcd for [C_11_H_27_B_12_Cl_2_N_3_–H]^−^: 400.2699. Found: 400.2714.

*Synthesis of urea [NBu*_4_*]*_2_(**7a**): In a glovebox filled with N_2_, a 20 mL vial was charged with [Et_3_NH][B_12_H_11_NH_3_] (260 mg, 1.00 mmol, 1 equiv), NaH (53 mg, 2.2 mmol, 2.2 equiv) and a stir bar. THF (10 mL) was added, and the mixture was stirred at room temperature for 10 min until there was no H_2_ evolution anymore. Phenyl isocyanate (238 mg, 2.0 mmol, 2 equiv) was slowly added. The conversion was complete after stirring for 5 h. The flask was transferred out of the glovebox. The solvent was removed under vacuum, and H_2_O (10 mL) was added. The aqueous solution was heated to 50 °C, and [NBu_4_]Br (677 mg, 2.1 mmol, 2.1 equiv) was added. A white solid precipitated immediately and was collected by filtration. It was dried under vacuum overnight to afford [NBu_4_]_2_[**7a**] as a colorless microcrystalline product (685 mg, 90%). ^1^H{^11^B} NMR (400 MHz, CD_3_CN): δ = 8.52 (broad s, 1H, anionic NH), 7.41 (d, 2H, *J* = 8.2 Hz, Ph H), 7.18 (dd, 2H, *J* = 8.2 Hz, 7.6 Hz, Ph–H), 6.83 (t,1H, *J* = 7.6 Hz, Ph–H), 3.96 (broad s, 1H, NH), 3.25–3.01 (m, 16H, cationic N–C*H*_2_), 1.67-1.50 (m, 16H, cationic N-CH_2_C*H*_2_), 1.41–1.27 (overlapping m and s, 21H, cationic N–CH_2_CH_2_C*H*_2_ and B–H), 1.04 (s, 5H, B–H), 0.95 (t, 24H, *J* = 7.3 Hz, cationic C*H*_3_), 0.85 (s, 1H, B–H). ^13^C{^1^H} NMR (101 MHz, CD_3_CN): δ = 158.6, 142.8, 129.5 (overlapping signals), 121.2, 59.2, 24.3, 20.3, 10.8. ^11^B{^1^H} NMR (128 MHz, CD_3_CN): δ = –5.0 (1B, B–N), –15.4 (5B, B–H), –16.2 (5B, B–H), –19.3 (1B, B–H). High-resolution ESI-MS (negative mode, MeOH): *m*/*z* calcd for [C_7_H_18_B_12_N_2_O]^2−^ 138.1320. Found: 138.1331.

*Synthesis of urea [PPh*_4_*]*_2_(**7b**): This product was prepared in a similar manner to [NBu_4_]_2_[**7a**], using 4-chlorophenyl isocyanate (307 mg, 2.0 mmol, 2 equiv) and [PPh_4_]Br (881 mg, 2.1 mmol, 2.1 equiv). [PPh_4_]_2_[**7b**] was obtained as a colorless microcrystalline solid (869 mg, 91%). ^1^H{^11^B} NMR (400 MHz, CD_3_CN): δ = 8.59 (s, 1H, anionic NH), 7.95-7.85 (m, 8H, cationic H), 7.81–5.58 (overlapping m, 32H, cationic H), 7.41–7.28 (m, 2H, Ph–H), 7.13–6.96 (m, 2H, Ph–H), 4.00 (s, 1H, N–H), 1.33 (broad signal, 5H, B–H), 1.07 (broad signal, 5H, B–H), 0.88 (broad signal, 1H, B–H). ^13^C{^1^H} NMR (101 MHz, CD_3_CN): δ = 158.4, 141.7, 136.4 (d, *J*_P,C_ = 2.4 Hz, cation CH), 135.6 (d, *J*_P,C_ = 10 Hz, cation CH), 131.3 (d, *J*_P,C_ =13.0 Hz, cation CH), 129.2, 124.9, 119.6, 118.8 (d, *J*_P,C_ = 89 Hz, cation C_q_). ^11^B{^1^H} NMR (128 MHz, CD_3_CN): δ = −5.0 (1B, B–N), −15.5 (5B, B–H), −16.2 (5B, B-H), −19.2 (1B, B–H). High-resolution ESI-MS (negative mode, MeOH): *m*/*z* calcd for [C_7_H_17_B_12_N_2_OCl]^2−^ 155.1125. Found: 155.1133.

*Synthesis of isocyanate [MePPh*_3_*]*_2_(**8**): In a glovebox filled with N_2_, a 50 mL round-bottom flask was charged with Cs[B_12_H_11_NH_3_] (594 mg, 2.0 mmol, 1 equiv), NaH (144 mg, 6.0 mmol, 3 equiv) and a stir bar. DMF (10 mL) was added, and the mixture was stirred at 25 °C for 10 min until there was no H_2_ evolution anymore. Then ClC(O)NMe_2_ (6 equiv) diluted in DMF (2 mL) was slowly added by an Eppendorf pipet. The conversion was complete after stirring for 4 h. The flask was transferred out of the glovebox, and the volatiles were removed under vacuum. The residue was dissolved in H_2_O (10 mL) at *ca.* 90 °C, giving a slightly yellow solution. The solution was stirred at 80–100 °C for 1 h, and [MePPh_3_]Br (1.29 g, 5 mmol, 2.5 equiv) was added. A white precipitate formed, and it was was collected by filtration. Purification by column chromatography (eluent DCM/MeCN 4:3) afforded [MePPh_3_]_2_[8] as a colorless solid (369 mg, 25%). ^1^H{^11^B} NMR (400 MHz, CD_3_CN): δ = 7.90–7.83 (m, 6H, cationic CH), 7.76–7.62 (overlapping m, 24H, cationic CH), 2.83 (d, *J* = 13.8 Hz, 6H, CH_3_), 1.23 (broad signal, 5H, B–H), 0.97 (broad signal, 5H, B-H), 0.75 (broad signal, 1H, B–H). ^13^C{^1^H} NMR (101 MHz, CD_3_CN): δ = 136.1 (d, *J*_P,C_= 3.0 Hz, cation CH), 134.2 (d, *J*_P,C_= 11 Hz, cation CH), 131.1 (d, *J*_P,C_= 13 Hz, cation CH), 120.4 (d, *J*_P,C_= 89 Hz, cation C_q_), 9.37 (d, ^1^*J*_P,C_= 58 Hz, cation CH_3_). The N=C=O carbon atom could not be detected unambiguously. ^11^B{^1^H} NMR (128 MHz, CD_3_CN): δ = −7.74 (1B, B–N), −15.4 (5B, B–H), −16.7 (5B, B–H), −19.6 (1B, B–H). Mass-spectrometric characterization of this product proved difficult; the results that were obtained by negative-mode ESI-MS are shown in Figure S2, along with the IR spectrum in Figure S3.

Additional figures, X-ray crystallographic data and copies of NMR spectra are provided in the Supporting Information file; see “Supplementary Materials”.

## 4. Conclusions

In summary, the synthesis and characterization of a series of compounds based on the amino-dodecaborate anion **2** has been achieved, including amidinium ions **6**, amides **3**, ureas **7** and dodecaboranyl isocyanate **8**. The new products have been fully characterized spectroscopically, and solid-state structures have been elucidated for nine derivatives. Amidinium ions **7** are reported for the first time, as well as aromatic ureas of the structure {B_12_}NH-C(O)-NHAr. A facile method for the interconversion of the dianionic and monoanionic form of amides **3** has been developed, which is of relevance in view their different physical properties, in particular solubility and crystallinity. A fragmentation-based approach to dodecaboranyl isocyanate **8** was developed, which is expected to provide access to novel {B_12_} derivatives by subsequent nucleophilic addition to the NCO group or by pericyclic reactions. The transformations leading to **6**, **7** and **8** extend the synthetic toolbox to produce boron clusters for applications in various areas.

## Figures and Tables

**Table 1:** Selected bond lengths and angles for **3a**, **3a-H 3b**, **3c**, **3d-H**, **3e** and **3e-H**.

Distance [Å]/Angle [°]	3a ^1^	3a-H ^2^	3b	3c	3d-H	3e	3e-H
B1–N1	1.520(6)	1.534(4)	1.516(4)	1.512(5)	1.577(7)	1.516(3)	1.524(3)
N1–C1	1.324(6)	1.295(7)	1.320(4)	1.327(4)	1.263(7)	1.306(2)	1.316(3)
C1–O1	1.250(5)	1.314(2)	1.238(3)	1.227(4)	1.343(8)	1.234(2)	1.232(3)
C1–C2	1.496(6)	1.471(2)	1.500(4)	1.500(5)	1.466(17)	1.502(3)	1.512(3)
Σ(C1)	359.9	359.9	360.0	360.0	359.9	360.0	360.0
O1-C1-C2-C3 ^3^	–17.8(3)	–9.95(13)	–15.6(4)	32.0(3)	–4.9(4)	5.77(2)	7.4(3)

^1^ Parameters of one of the two molecules in the asymmetric unit; ^2^ data from reference [34]; ^3^ torsion angle O1-C1-C2-N2 for **3e-H**.

## Supplementary Materials

The following are available online: Supporting Information file with experimental procedures and spectroscopic data. Crystal structures have been deposited with the Cambridge Crystallographic Data Centre: CCDC1861483–1861492. They are available free of charge from www.ccdc.cam.ac.uk.
