# Supplementary material for: Synthesis and Structural Characterization of Amidine, Amide, Urea and Isocyanate Derivatives of the Amino-closo-dodecaborate Anion [B12H11NH3]−
Source: Molecules. 2018 Nov 29;23(12):3137. doi: 10.3390/molecules23123137 (PMC6321512; doi:10.3390/molecules23123137)
Supplement: Supplementary file 1 [file molecules-23-03137-s001.pdf]

Supplementary Information for

Synthesis and X-ray structural characterization of amidine, amide, urea and isocyanate derivatives of the *closo*-aminododecaborate anion $[B_{12}H_{11}(NH_3)]^-$

Yuanbin Zhang[1, 2‡], Yuji Sun[1‡], Tao Wang[1], Jiyong Liu[1], Bernhard Spingler[3] and Simon Duttwyler[1,*]

[‡]These authors contributed equally.

[1] Department of Chemistry, Zhejiang University 38 Zheda Road, 310027 Hangzhou, P. R. China
[2] Key Laboratory of Biomass Chemical Engineering of Ministry of Education, Department of Chemical and Biological Engineering, Zhejiang University, 38 Zheda Road, 310027 Hangzhou, P. R. China
[3] Department of Chemistry, University of Zurich, Winterthurerstrasse 190, 8057 Zurich, Switzerland
* Correspondence: duttwyler@zju.edu.cn

Table of Contents

**I**	**General Information**	**p. S2–S3**
**II**	**Experimental Section**	**p. S4–S14**
**III**	**X-ray Crystallography**	**p. S15–S35**
**IV**	**References**	**p. S36**
**V**	**NMR spectra**	**p. S37–S68**

I General Information

Chemicals

If not otherwise specified, reagents and organic solvents were commercially available and used without further purification. Anhydrous solvents were prepared by passage through activated Al_2O_3 and stored over 3 Å molecular sieves. CD_3CN and CD_2Cl_2 were purchased from Cambridge Isotope Laboratories and filtered through Al_2O_3 prior to use. $[B_{12}H_{12}]^{2-}$ and $[B_{12}H_{11}NH_3]^{-}$ salts and dodecaborate amides **3a**–**e** were prepared according to the literature.[1–3]

Reaction Conditions

Glassware for air-sensitive reations was dried at 150 °C and allowed to cool in a vacuum. Reactions carried out in a glovebox were run under a nitrogen atmosphere with O_2, H_2O <1 ppm.

Characterization

Thin-layer chromatography (TLC) was carried out using silica gel 60, F254 with a thickness of 0.25 mm. Column chromatography was performed on silica gel 60 (200-30 mesh).

Low-resolution ESI-MS data were recorded on Advion Expression CMS instrument. High-resolution MS data were recorded using IT-TOF detection (Shimadzu, Japan) equipped with an electrospray ionization source (ESI). Accurate mass determination was corrected by calibration using sodium trifluoroacetate clusters as a reference.

Single-crystal X-ray diffraction studies were performed on an Oxford Diffraction Gemini A Ultra diffractometer equipped with an 135mm Atlas CCD detector and using Mo K-α radiation

NMR spectra were recorded on a Bruker AVANCE III 500 spectrometer (^{1}H NMR 500.13 MHz, ^{13}C NMR 125.77 MHz, ^{11}B NMR 160.46 MHz) or a Bruker AVANCE III 400 spectrometer (^{1}H NMR 400.13 MHz, ^{13}C NMR 100.62 MHz, ^{11}B NMR 128.38 MHz) at the temperature indicated. Data are reported as follows: Chemical shift in ppm, multiplicity (s = singlet, d = doublet, t = triplet, q = quartet, m = multiplet, dd = doublet of doublets, etc.), coupling constant *J* in Hz, integration, and (where applicable) interpretation. Signals were referenced against solvent peaks (^{1}H: residual $CHD_2C(O)CD_3$ = 2.05 ppm, residual CHD_2CN = 1.94 ppm, residual $CHDCl_2$ = 5.32 ppm, ^{13}C{^{1}H}: $\mathit{C}D_3C(O)\mathit{C}D_3$ = 29.84 ppm, $\mathit{C}D_3CN$ = 1.32 ppm, CD_2Cl_2 = 53.32 ppm). ^{11}B and ^{11}B{^{1}H} NMR spectra were calibrated against external BF_3*Et_2O = 0 ppm (BF_3*Et_2O capillary in C_6D_6).

II Experimental Section

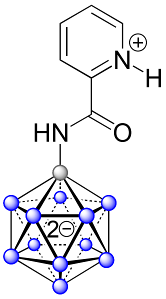

[$Et_3NH]^+$

Synthesis of [Et$_3$NH][3e-H]: In a glovebox filled with N_2, a 20 mL vial was charged with [Et$_3$NH][$B_{12}H_{11}NH_3$] (212.4 mg, 0.817 mmol, 1 equiv), NaH (138.2 mg, 5.758 mmol, 7 equiv) and a stir bar. THF (4 mL) and DMF (4 mL) were added, and the mixture was stirred at room temperature for 10 minutes until there was no H_2 evolution anymore. Then pyridine-2-carbonyl chloride hydrochloride PyCOCl·HCl (220.2 mg, 1.237 mmol, 1.5 equiv) was slowly added. The conversion was complete after stirring for 5 h. The flask was transferred out of the glovebox. H_2O (4 mL) was added, and the pH value of the reaction mixture was adjusted to 2–3 with 1 M aqueous HCl. [NEt$_3$H]Cl (300 mg, 2.180 mmol, 2.7 equiv) was added, and the reaction mixture was extracted with MeCN/EtOAc (1:2 *v*/*v*). The organic layers were concentrated on a rotary evaporator. The residue was purified by recrystallization from methanol to afford yellowish crystals of [Et$_3$NH][**3e-H**] (150 mg, 50%).

$^1H\{^{11}B\}$ NMR (400 MHz, CD_3CN): δ = 8.96 (s, 1H, anionic N*H*), 8.90-8.86 (m, 1H, Py H), 8.18-8.14 (overlapping m, 2H, Py H), 7.89-7.72 (m, 1H, Py H), 6.63 (t,1H, J_{NH} = 52 Hz, N*H*), 3.27 (s, 1H, N*H*), 3.20-3.15 (m, 6H, cationic N-CH_2), 1.47 (broad signal, 5H, B-H), 1.24 (t, *J* = 7.4 Hz, 9H, cationic CH_3), 1.20 (broad signal, 5H, B-H), 1.13 (broad signal, 1H, B-H).

$^{13}C\{^1H\}$ NMR (101 MHz, CD_3CN): δ = 166.7, 149.5, 143.9, 141.5, 129.8, 124.5 (6 anionic signals), 48.0, 9.2 (2 cationic signals).

$^{11}B\{^1H\}$ NMR (128 MHz, CD_3CN): δ = -7.6 (1B, *B*-N), -15.3 (5B, *B*-H), -15.7 (overlapping signals, 6B, *B*-H).

High-resolution ESI-MS (negative mode, MeOH): *m*/*z* calcd for $[C_6H_{17}B_{12}N_2O]^-$ 263.2430. Found: 263.2459.

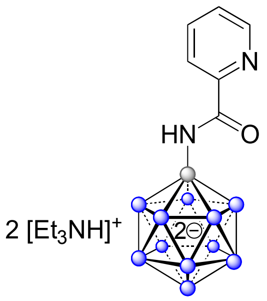

Transformation of [Et_3NH][3e-H] to [Et_3NH]$_2$[3e]: A 20 mL vial was charged with [Et_3NH][**3e-H**] (50 mg) and a stir bar. MeCN (3 mL) and Et_3N (0.5 mL) were added, and the solution was stirred at room temperature for 1 h. Then the stir bar was removed, and the solution was concentrated on a rotary evaporator and dried overnight under vacuum at 80 °C to afford compound [Et_3NH]$_2$[**3e**] in quantitative yield.

This method can also be applied for the transformation of other compounds **3-H** to **3** quantitatively. $^{11}B\{^1H\}$ NMR spectra of **3b**, **3b-H**, **3e** and **3e-H** are displayed in Figure S1.

$^1H\{^{11}B\}$ NMR (400 MHz, CD_3CN): δ = 8.56 (broad signal, 1H, Py H), 8.09-8.00 (m, 1H, Py H), 7.99-7.80 (overlapping m, 2H, Py H and amide N-H), 7.50-7.38 (m, 1H, Py H), 4.63 (broad t, 2H, J_{NH} = 52 Hz, N-H from cation), 3.25-3.01 (m, 12H, cationic N-CH_2), 1.34 (s, 5H, B-H), 1.24 (t, J = 7.4 Hz, 9H, cationic CH_3), 1.03 (broad signal, 5H, B-H), 0.89 (broad signal, 1H, B-H).

$^{13}C\{^1H\}$ NMR (101 MHz, CD_3CN): δ = 166.2, 152.9, 149.0, 138.5, 126.4, 122.2 (6 anionic signals), 47.8, 9.1 (2 cationic signals).

$^{11}B\{^1H\}$ NMR (128 MHz, CD_3CN): δ = -5.3 (1B, B-N), -15.3 (5B, B-H), -16.4 (5B, B-H), -18.7 (1B, B-H).

High-resolution ESI-MS (negative mode, MeOH): *m/z* calcd for $[C_6H_{17}B_{12}N_2O]^{2-}$ 131.1226. Found: 131.1254.

The $^{11}B\{^{1}H\}$ NMR spectra of **3b**, **3b-H**, **3e** and **3e-H** are shown in Figure S1 as representative examples to demonstrate the effect of protonation. For both product pairs **3b**/**3b-H** and **3e**/**3e-H**, similar effects are observed. Upon protonation, the B–N signal is shifted from –5 ppm to –8 ppm. On the other hand, the B–H vertices become more deshielded; the B12 signal appears at –19 ppm in the dianionic form and overlaps with the B2–11 resonances in the monoanionic form.

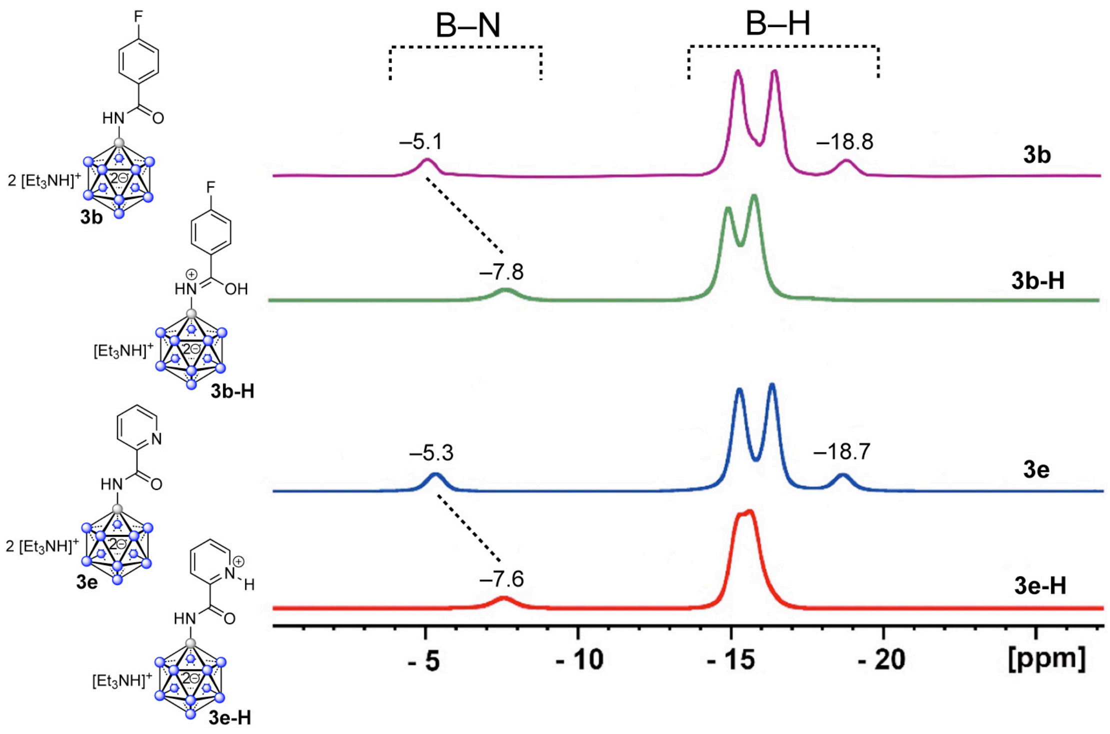

Figure S1. $^{11}B\{^{1}H\}$ NMR spectra of **3b**, **3b-H**, **3e** and **3e-H** (acetonitrile-d_3,128 MHz, 23 °C).

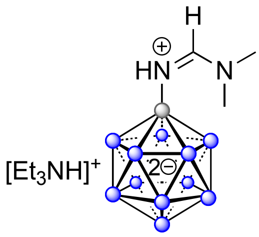

Synthesis of amidine [Et_3NH][6a]: In a glovebox, a dry 20 mL vial, equipped with a stir bar, was charged with [Et_3NH][$B_{12}H_{11}NH_3$] (102 mg, 0.40 mmol, 1 equiv). Then anhydrous DMF (1 mL) was added. The vial was transferred to a fumehood, and dry Et_3N (1.0 mL, 7.20 mmol, 18 equiv) was added to the solution under N_2 protection. Then 2,4,6-trimethylphenylcarboxylic acid chloride (110 mg, 0.60 mmol, 1.5 equiv) was added. The mixture was stirred at 25 °C for 4 h. The reaction was quenched with an aqueous [Et_3NH]Cl solution (2 mL H_2O + 2 equiv [Et_3NH]Cl); the pH value at this point was ca. 7–8. The mixture was extracted with DCM/MeCN = 4 : 1 (8 x 10 mL). The combined organic layers were dried over $MgSO_4$, and the solution was filtered and concentrated by rotary evaporation. The cloudy residue was purified by silica gel column chromatography (eluent DCM/MeCN = 10:3, fraction size 20 mL). The combined eluates were concentrated on a rotary evaporator and dried under vacuum at 60 °C overnight to afford compound [Et_3NH][**6a**] as a colorless solid (50.4 mg, 40%).

$^{1}H\{^{11}B\}$ NMR (400 MHz, CD_3CN, 23 °C): δ 7.76 (d, J = 16.0 Hz, 1H, N=CH-N), 6.41 (broad signal, 1H, N-H), 3.13 (q, J = 7.2 Hz, 6H, cationic N-CH2), 3.08 (s, 3H, anionic N-CH_3), 2.83 (s, 3H, anionic N-CH_3), 1.26 (broad signal, 5H, B-H), 1.24 (t, J = 7.2 Hz, 9H, cationic N-CH_2CH_3), 1.03 (broad signal, 5H, B-H), 0.85 (broad signal, 1H, B-H).

$^{13}C\{^{1}H\}$ NMR (100 MHz, CD_3CN, 23 °C): δ 157.3 (N=C-N), 48.0 (cationic CH_2), 43.1, 35.7 (two N-C signals), 9.2 (cationic CH_3).

$^{11}B\{^{1}H\}$ NMR (160 MHz, CD_3CN, 23 °C): δ -4.2 (1B, B-N), -14.5 to -17.0 (10B, B-H), -19.0 (1B, B-H).

High-resolution ESI-MS (negative mode, MeOH): m/z calcd for $[C_3H_{19}B_{12}N_2]^-$: 213.2738. Found: 213.2762.

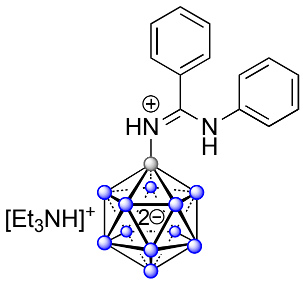

Synthesis of amidine $[Et_3NH]$[6b]: A dry 20 mL vial, equipped with a stir bar, was charged with $[Et_3NH]_2[B_{12}H_{11}NHCOC_6H_5]$ (101 mg, 0.22 mmol, 1 equiv). Then anhydrous MeCN (3 mL) was added, and dry Et_3N (0.3 mL, 2.16 mmol, 9.8 equiv) was added to the solution under N_2 protection. Pentafluorophenylcarboxylic acid chloride (80.0 mg, 0.35 mmol, 1.5 equiv) was added at 25 °C. The temperature was raised to 50 °C. After 30 min, aniline (61 mg, 0.66 mmol, 3.0 equiv) was added. The mixture was stirred for another 4 h and concentrated by rotary evaporation. The cloudy residue was purified by silica gel column chromatography (eluent DCM/MeCN = 4:1, fraction size 20 mL). The combined eluates were concentrated on a rotary evaporator and dried under vacuum at 60 °C overnight to afford compound $[Et_3NH]$[**6b**] as a yellow solid (87.7 mg, 91%).

$^1H\{^{11}B\}$ NMR (400 MHz, CD_2Cl_2, 23 °C): δ 10.00 (s, 1H, N-H), 7.53-7.48 (m, 1H, phenyl H), 7.41-7.35 (overlapping m, 4H, phenyl H), 7.24-7.09 (overlapping m, 3H, phenyl H), 7.03-6.78 (overlapping broad signal and m, 3H, phenyl H and N-H), 6.65 (broad signal, 1H, N-H) 3.29-3.22 (m, 6H, cationic N-CH_2), 1.62 (broad signal, 5H, B-H), 1.40 (t, J = 7.2 Hz, 9H, cationic N-$CH_2C\mathit{H_3}$), 1.22 (broad signal, 5H, B-H), 1.05 (broad signal, 1H, B-H).

This spectrum contained small signals at 7.18, 6.71 and 6.67 ppm ascribed to residual aniline

$^{13}C\{^1H\}$ NMR (100 MHz, CD_3CN, 23 °C): δ 165.6 (N=C-N), 138.1, 133.2, 131.3, 130.4, 130.1, 129.9, 127.5, 125.6 (8 aryl signals), 48.3 (cationic N-CH_2), 9.4 (cationic N-CH_3).

This spectrum showed small signals at 149.1, 130.2, 118.3 and 115.6 ppm ascribed to residual aniline.

$^{11}B\{^1H\}$ NMR (128 MHz, CD_2Cl_2, 23 °C): δ -5.8 (1B, B-N), -13.5 to -16.5 (10B, B-H), -17.4 (1B, B-H).

High-resolution ESI-MS (negative mode, MeOH): *m/z* calcd for $[C_{13}H_{23}B_{12}N_2]^-$: 337.3056. Found: 337.2382.

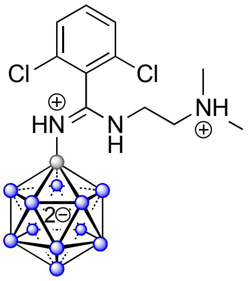

Synthesis of amidine [Et_3NH][6c]: A dry 20 mL vial, equipped with a stir bar, was charged with $[Et_3NH]_2[B_{12}H_{11}NHCOC_6H_3Cl_2]$ (177 mg, 0.33 mmol, 1 equiv). Then anhydrous MeCN (3 mL) was added, and dry Et_3N (0.45 mL, 3.25 mmol, 9.8 equiv) was added to the solution under N_2 protection. Pentafluorophenylcarboxylic acid chloride (128 mg, 0.55 mmol, 1.7 equiv) was added at 25 °C. The temperature was raised to 50 °C. After 30 min, *N,N*-dimethylethylamine (88 mg, 1.00 mmol, 3.0 equiv) was added. The mixture was stirred for another 4 h, and 1 M aqueous HCl (5 mL) was added. The suspension was extracted with EtOAc/MeCN 3:1 (5 x 10 mL). The combined organic layers were dried over $MgSO_4$, and the solution was filtered and concentrated by rotary evaporation. The cloudy residue was purified by silica gel column chromatography (eluent DCM/MeCN = 4:3, fraction size 20 mL). The combined eluates were concentrated and dried under vacuum at 60 °C overnight to afford compound [Et_3NH][**6c**] as a yellow solid (132 mg, 100%).

$^{1}H\{^{11}B\}$ NMR (400 MHz, CD_3CN, 23 °C): δ 8.54 (broad signal, 1H, N-H), 7.59-7.55 (overlapping m, 3H, aryl H), 7.46 (broad signal, 1H, N-H), 6.98 (very broad signal, 1H, N-H), 3.43 (dt, J = 7.2 Hz, 7.2 Hz, 2H, CH_2), 3.24 (t, J = 7.2 Hz, 2H, CH_2), 2.77 (s, 6H, N-CH_3), 1.41 (broad signal, 5H, B-H), 1.12 (broad signal, 5H, B-H), 1.06 (broad signal, 1H, B-H).

$^{13}C\{^{1}H\}$ NMR (100 MHz, CD_3CN, 23 °C): δ 161.9 (N=C-N), 134.6, 134.2, 129.8, 129.2 (4 aryl signals), 56.9, 44.8, 40.0.

$^{11}B\{^{1}H\}$ NMR (128 MHz, CD_3CN, 23 °C): δ -6.9 (1B, B-N), -13.0 to -18.0 (overlapping signals with peaks at -15.2 and -16-1 ppm, 11B, B-H).

High-resolution ESI-MS (negative mode, MeOH): m/z calcd for $[C_{11}H_{27}B_{12}Cl_2N_3\text{-}H]^-$: 400.2699. Found: 400.2714.

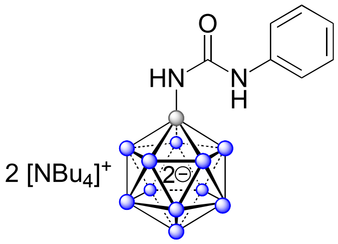

Synthesis of urea $[NBu_4]_2$[7a]: In a glovebox filled with N_2, a 20 mL vial was charged with $[Et_3NH][B_{12}H_{11}NH_3]$ (260 mg, 1.00 mmol, 1 equiv), NaH (53 mg, 2.2 mmol, 2.2 equiv) and a stir bar. THF (10 mL) was added, and the mixture was stirred at room temperature for 10 minutes until there was no H_2 evolution anymore. Phenyl isocyanate (238 mg, 2.0 mmol, 2 equiv) was slowly added. The conversion was complete after stirring for 5 h. The flask was transferred out of the glovebox. The solvent was removed under vacuum, and H_2O (10 mL) was added. The aqueous solution was heated to 50 °C, and $[NBu_4]Br$ (677 mg, 2.1 mmol, 2.1 equiv) was added. A white solid precipitated immediately and was collected by filtration. It was dried under vacuum overnight to afford $[NBu_4]_2$[**7a**] as a colorless microcrystalline product (685 mg, 90%).

$^{1}H\{^{11}B\}$ NMR (400 MHz, CD_3CN): δ = 8.52 (broad s, 1H, anionic NH), 7.41 (d, 2H, *J* = 8.2 Hz, Ph H), 7.18 (dd, 2H, *J* = 8.2 Hz, 7.6 Hz, Ph *H*), 6.83 (t,1H, *J* = 7.6 Hz, Ph *H*), 3.96 (broad s, 1H, N*H*), 3.25-3.01 (m, 16H, cationic N-CH_2), 1.67-1.50 (m, 16H, cationic N-CH_2CH_2), 1.41-1.27 (overlapping m and s, 21H, cationic N-CH_2CH_2CH_2 and B-*H*), 1.04 (s, 5H, B-H), 0.95 (t, 24H, *J* = 7.3 Hz, cationic CH_3), 0.85 (s, 1H, B-H).

$^{13}C\{^{1}H\}$ NMR (101 MHz, CD_3CN): δ = 158.6, 142.8, 129.5 (overlapping signals), 121.2, 59.2, 24.3, 20.3, 10.8.

$^{11}B\{^{1}H\}$ NMR (128 MHz, CD_3CN): δ = -5.0 (1B, B-N), -15.4 (5B, B-H), -16.2 (5B, B-H), -19.3 (1B, B-H).

High-resolution ESI-MS (negative mode, MeOH): *m*/*z* calcd for $[C_7H_{18}B_{12}N_2O]^{2}$ 138.1320. Found: 138.1331.

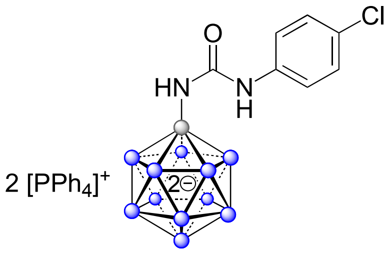

Synthesis of urea $[PPh_4]_2$[7b]: This product was prepared in a similar manner to $[NBu_4]_2$[**7a**], using 4-chlorophenyl isocyanate (307 mg, 2.0 mmol, 2 equiv) and $[PPh_4]$Br (881 mg, 2.1 mmol, 2.1 equiv). $[PPh_4]_2$[**7b**] was obtained as a colorless microcrystalline solid (869 mg, 91%).

^{1}H{^{11}B} NMR (400 MHz, CD_3CN): δ = 8.59 (s, 1H, anionic NH), 7.95-7.85 (m, 8H, cationic H), 7.81-5.58 (overlapping m, 32H, cationic H), 7.41-7.28 (m, 2H, Ph H), 7.13-6.96 (m, 2H, Ph H), 4.00 (s, 1H, N-H), 1.33 (broad signal, 5H, B-H), 1.07 (broad signal, 5H, B-H), 0.88 (broad signal, 1H, B-H).

^{13}C{^{1}H} NMR (101 MHz, CD_3CN): δ = 158.4, 141.7, 136.4 (d, $J_{P,C}$ = 2.4 Hz, cation CH), 135.6 (d, $J_{P,C}$ = 10 Hz, cation CH), 131.3 (d, $J_{P,C}$ =13.0 Hz, cation CH), 129.2, 124.9, 119.6, 118.8 (d, $J_{P,C}$ = 89 Hz, cation C_q).

^{11}B{^{1}H} NMR (128 MHz, CD_3CN): δ = -5.0 (1B, B-N), -15.5 (5B, B-H), -16.2 (5B, B-H), -19.2 (1B, B-H).

High-resolution ESI-MS (negative mode, MeOH): *m*/*z* calcd for $[C_7H_{17}B_{12}N_2OCl]^{2-}$ 155.1125. Found: 155.1133.

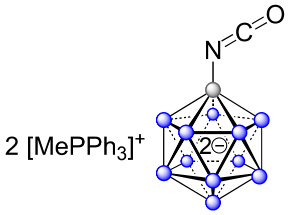

Synthesis of isocyanate [$MePPh_3$]$_2$[8]: In a glovebox filled with N_2, a 50 mL round-bottom flask was charged with Cs[$B_{12}H_{11}NH_3$] (594 mg, 2.0 mmol, 1 equiv), NaH (144 mg, 6.0 mmol, 3 equiv) and a stir bar. DMF (10 mL) was added, and the mixture was stirred at 25 °C for 10 minutes until there was no H_2 evolution anymore. Then ClC(O)NMe_2 (6 equiv) diluted in DMF (2 mL) was slowly added by an Eppendorf pipet. The conversion was complete after stirring for 4 h. The flask was transferred out of the glovebox, and the volatiles were removed under vacuum. The residue was dissolved in H_2O (10 mL) at *ca.* 90 °C, giving a slightly yellow solution. The solution was stirred at 80–100 °C for 1 h, and [$MePPh_3$]Br (1.29 g, 5 mmol, 2.5 equiv) was added. A white precipitate formed, and it was was collected by filtration. Purification by column chromatography (eluent DCM/MeCN 4:3) afforded [$MePPh_3$]$_2$[**8**] as a colorless solid (369 mg, 25%).

$^{1}H\{^{11}B\}$ NMR (400 MHz, CD_3CN): δ = 7.90-7.83 (m, 6H, cationic CH), 7.76-7.62 (overlapping m, 24H, cationic CH), 2.83 (d, J = 13.8 Hz, 6H, CH_3), 1.23 (broad signal, 5H, B-H), 0.97 (broad signal, 5H, B-H), 0.75 (broad signal, 1H, B-H).

$^{13}C\{^{1}H\}$ NMR (101 MHz, CD_3CN): δ = 136.1 (d, $J_{P,C}$= 3.0 Hz, cation CH), 134.2 (d, $J_{P,C}$= 11 Hz, cation CH), 131.1 (d, $J_{P,C}$= 13 Hz, cation CH), 120.4 (d, $J_{P,C}$= 89 Hz, cation C_q), 9.37 (d, $^{1}J_{P,C}$= 58 Hz, cation CH_3). The N=C=O carbon atom could not be detected unambiguously.

$^{11}B\{^{1}H\}$ NMR (128 MHz, CD_3CN): δ = -7.74 (1B, B-N), -15.4 (5B, B-H), -16.7 (5B, B-H), -19.6 (1B, B-H).

Mass-spectrometric characterization of this product proved difficult; the results that were obtained by negative-mode ESI-MS are shown in Figure S2, along with the IR spectrum in Figure S3.

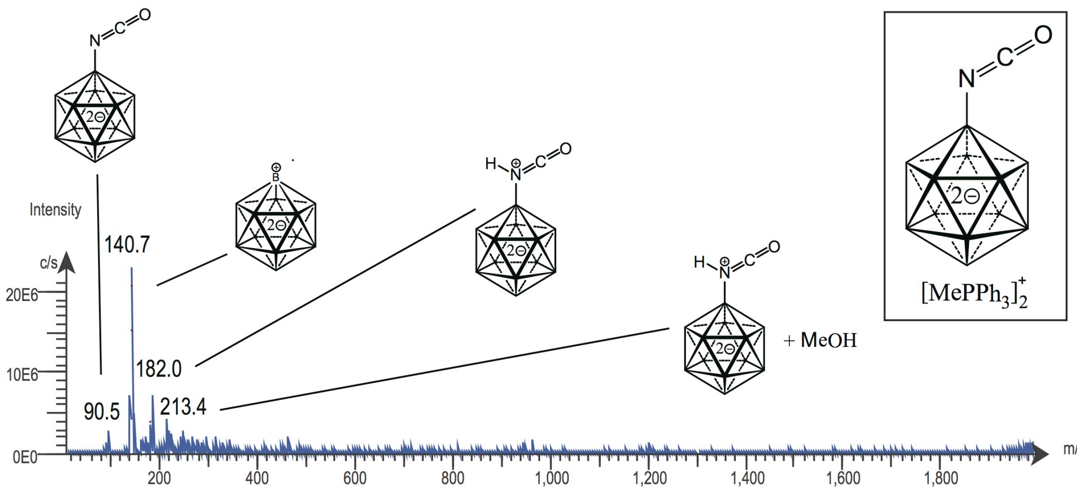

Figure S2. (–)-ESI Mass spectrum of **8** in MeOH.

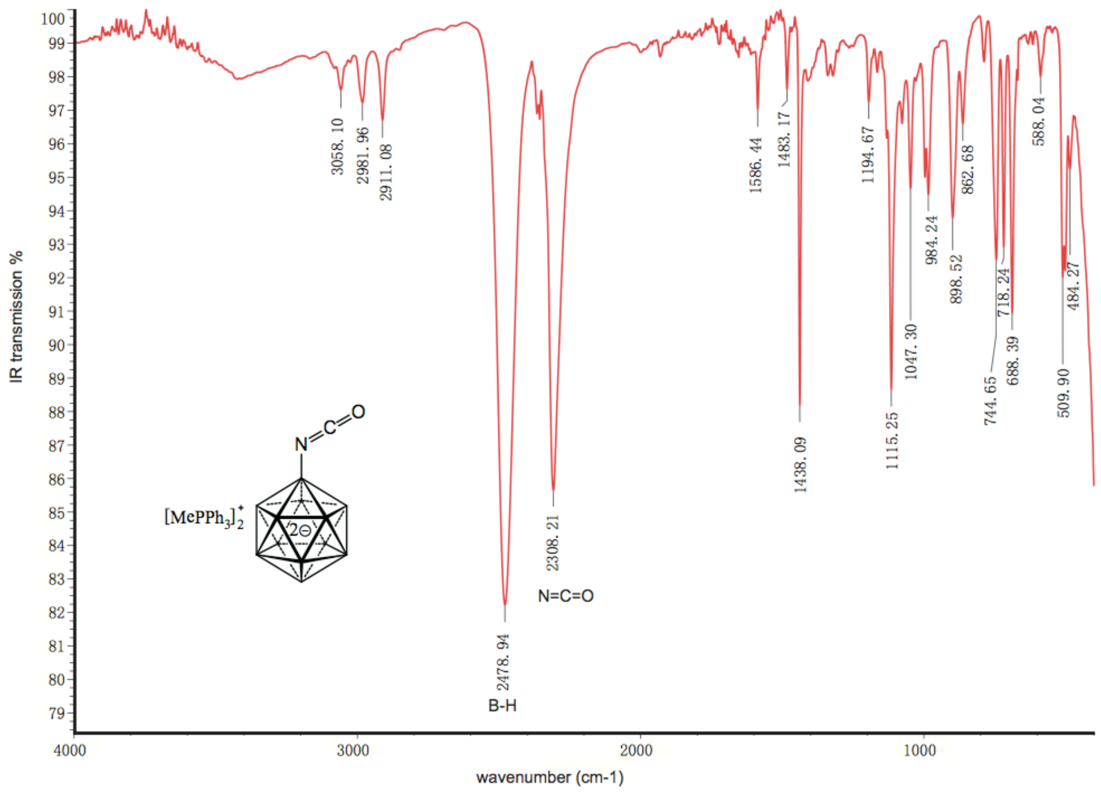

Figure S3. IR spectrum of $[PPh_4]_2$[**8**].

III X-ray Crystallography

CCDC1861483–1861492 contain the supplementary crystallographic data for this publication. These data can be obtained free of charge from the Cambridge Crystallographic Data Centre *via* www.ccdc.cam.ac.uk/data_request/cif.

Crystals of the products [Et_3NH]$_2$[**3b**], [Et_3NH][**3d-H**], [Et_3NH]$_2$[**3e**], [Et_3NH][**3e-H**], [$MePPh_3$][**6a**], [Et_3NH][**6c**] and [$MePPh_3$]$_2$[**8**] were measured at room temperature because the X-ray facility of our department does not routinely offer measurements with nitrogen cooling.

Crystal structure of $[Et_3NH]_2$[3a] (CCDC1861488)

Compound $[Et_3NH]_2$[**3a**] (20 mg) was dissolved in acetone/MeCN (0.25 mL/0.25 mL) in a 1 mL glass vial. The resulting colorless solution was filtered into an 18 cm long NMR tube and layered with hexanes (1 mL). Colorless crystals of the composition $[Et_3NH]_4[B_{12}H_{11}NHCOPh]_2 \cdot H_2O$ suitable for X-ray diffraction grew within 3 d at 25 °C.

```
Bond precision:   C-C = 0.0084 A          Wavelength=0.71073

Cell:             a=10.3802(8)      b=15.9133(12)     c=18.0577(15)
                  alpha=79.497(7)   beta=87.786(7)    gamma=87.828(6)
Temperature:      170 K

                   Calculated                  Reported
Volume             2929.2(4)                   2929.2(4)
Space group        P -1                        P -1
Hall group         -P 1                        -P 1
Moiety formula     2(C7 H17 B12 N O), 4(C6     2(C7 H17 B12 N O), 4(C6
                   H16 N), H2 O                H16 N), H2 O
Sum formula        C38 H100 B24 N6 O3          C38 H100 B24 N6 O3
Mr                 948.68                      948.67
Dx,g cm-3          1.076                       1.076
Z                  2                           2
Mu (mm-1)          0.060                       0.060
F000               1028.0                      1028.0
F000'              1028.24
h,k,lmax           12,19,21                    12,19,21
Nref               10756                       10623
Tmin,Tmax          0.972,0.977                 0.849,1.000
Tmin'              0.972

Correction method= # Reported T Limits: Tmin=0.849 Tmax=1.000
AbsCorr = MULTI-SCAN

Data completeness= 0.988            Theta(max)= 25.350

R(reflections)= 0.1239( 6692)       wR2(reflections)= 0.3535( 10623)

S = 1.034                   Npar= 655
```

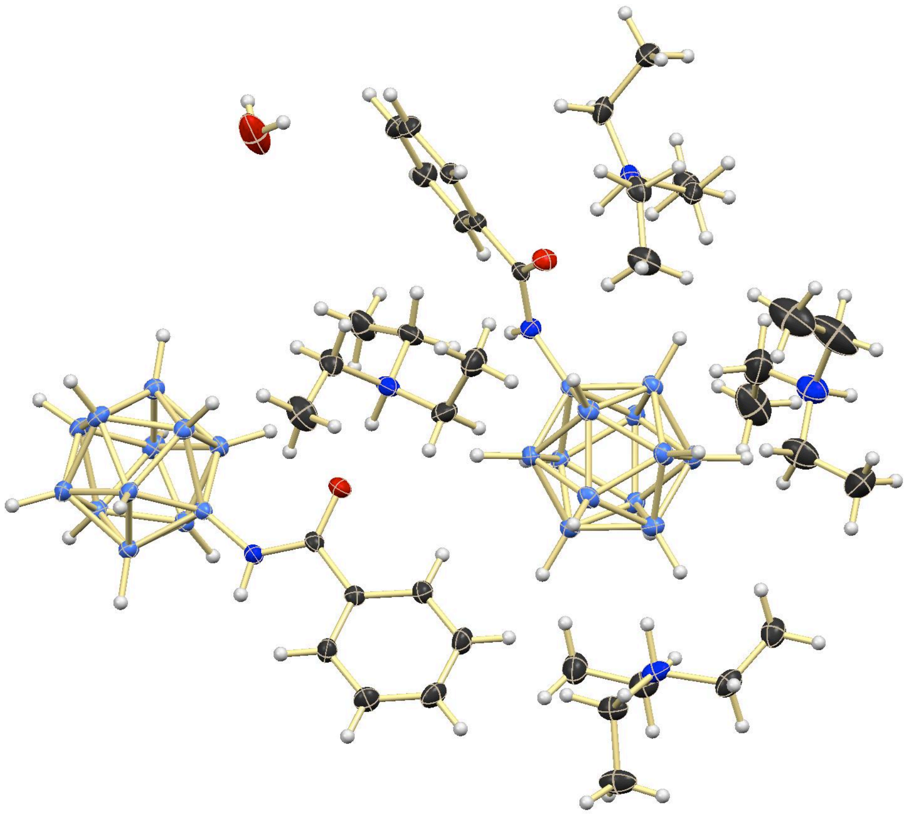

Figure S4. ORTEP representation of $[Et_3NH]_4[B_{12}H_{11}NHCOPh]_2{\cdot}H_2O$; 30% displacement ellipsoids.

Crystal structure of [$Et_3NH]_2$[3b] (CCDC1861489)

Compound [$Et_3NH]_2$[**3b**] (20 mg) was dissolved in acetone/MeCN (0.25 mL/0.25 mL) in a 1 mL glass vial. The resulting colorless solution was filtered into an 18 cm long NMR tube and layered with hexanes (1 mL). Colorless crystals of the composition [$Et_3NH]_2[B_{12}H_{11}NHCO-C_6H_4$-F]·$0.5CH_3C(O)CH_3$ suitable for X-ray diffraction grew within 1 d at 25 °C.

```
Bond precision:   C-C = 0.0060 A          Wavelength=0.71073

Cell:             a=17.517(2)    b=10.7703(8)        c=35.537(4)
                  alpha=90       beta=106.010(11)    gamma=90
Temperature:      293 K
```

	Calculated	Reported
Volume	6444.5(12)	6444.4(12)
Space group	C 2/c	C 1 2/c 1
Hall group	-C 2yc	-C 2yc
Moiety formula	2(C7 H16 B12 F N O), 4(C6 H16 N), C3 H6 O	2(C7 H16 B12 F N O), C3 H6 O, 4(C6 H16 N)
Sum formula	C41 H102 B24 F2 N6 O3	C41 H102 B24 F2 N6 O3
Mr	1024.73	1024.72
Dx,g cm-3	1.056	1.056
Z	4	4
Mu (mm-1)	0.063	0.063
F000	2208.0	2208.0
F000'	2208.65	
h,k,lmax	21,12,42	21,12,42
Nref	5906	5882
Tmin,Tmax	0.973,0.992	0.780,1.000
Tmin'	0.970	

```
Correction method= # Reported T Limits: Tmin=0.780 Tmax=1.000
AbsCorr = MULTI-SCAN

Data completeness= 0.996          Theta(max)= 25.350

R(reflections)= 0.0819( 3329)     wR2(reflections)= 0.2399( 5882)

S = 1.015                    Npar= 351
```

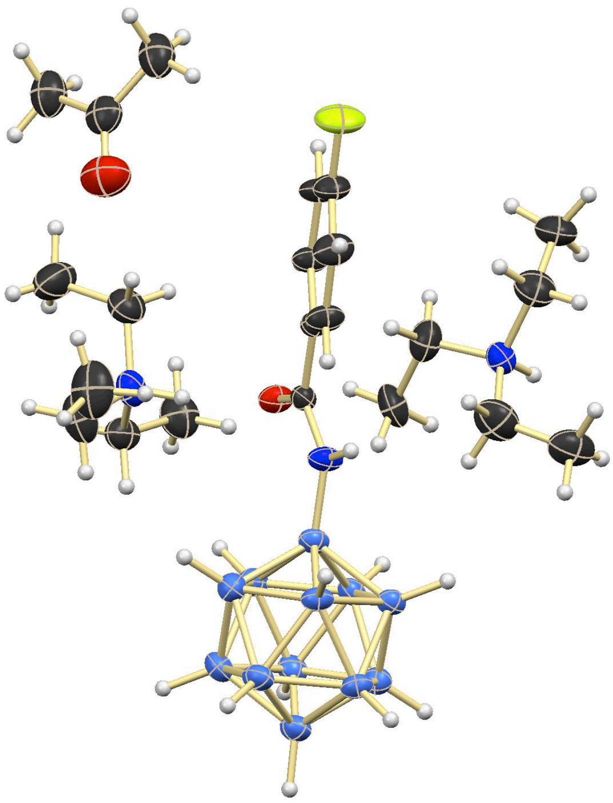

Figure S5. ORTEP representation of $[Et_3NH]_4[B_{12}H_{11}NHCO\text{-}C_6H_4\text{-}F]_2\cdot CH_3C(O)CH_3$; 30% displacement ellipsoids.

Crystal structure of [$Et_3NH]_2$[3c] (CCDC1861486)

Compound [$Et_3NH]_2$[**3c**] (20 mg) was dissolved in MeCN (0.5 mL) in a 1 mL glass vial. The resulting colorless solution was filtered into an 18 cm long NMR tube and layered with Et_2O (1 mL). Colorless crystals of the composition [$Et_3NH]_2$ [$B_{12}H_{11}NHCO-C_6H_4-I$] suitable for X-ray diffraction grew within 1 d at 25 °C.

Bond precision: C-C = 0.0060 A Wavelength=0.71073

Cell:	a=10.4220(6)	b=14.6666(8)	c=20.0458(10)
	alpha=90	beta=96.507(5)	gamma=90
Temperature:	181 K		

	Calculated	Reported
Volume	3044.4(3)	3044.4(3)
Space group	P 21/n	P 1 21/n 1
Hall group	-P 2yn	-P 2yn
Moiety formula	C7 H16 B12 I N O, 2(C6 H16 N)	C7 H16 B12 I N O, 2(C6 H16 N)
Sum formula	C19 H48 B12 I N3 O	C19 H48 B12 I N3 O
Mr	591.22	591.22
Dx,g cm-3	1.290	1.290
Z	4	4
Mu (mm-1)	1.071	1.071
F000	1216.0	1216.0
F000'	1214.35	
h,k,lmax	14,20,27	14,20,27
Nref	8520	7186
Tmin,Tmax	0.705,0.807	0.838,1.000
Tmin'	0.681	

Correction method= # Reported T Limits: Tmin=0.838 Tmax=1.000
AbsCorr = MULTI-SCAN

Data completeness= 0.843 Theta(max)= 29.551

R(reflections)= 0.0530(5330) wR2(reflections)= 0.1254(7186)

S = 1.077 Npar= 331

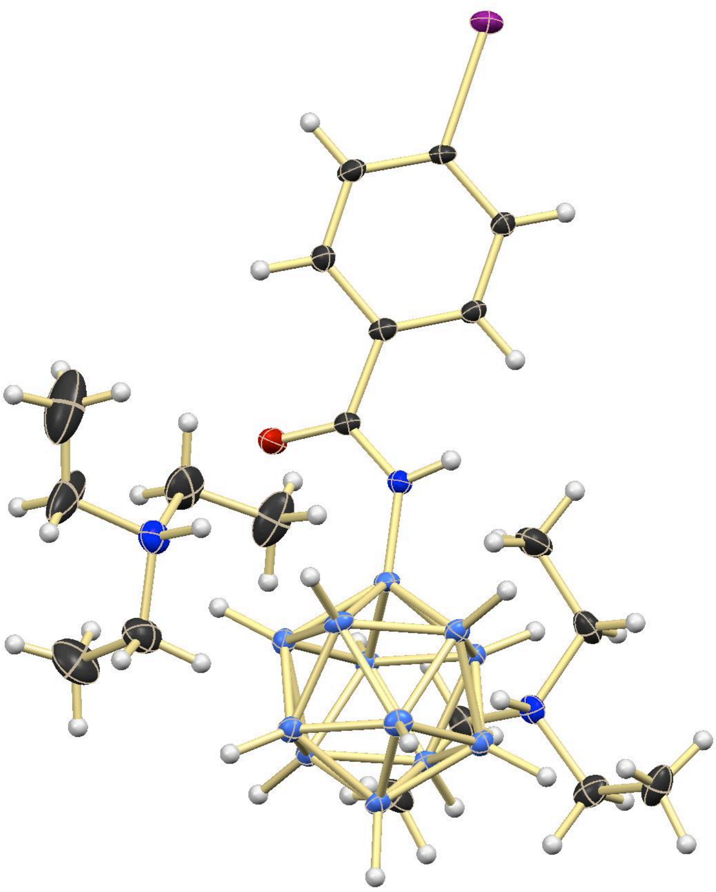

Figure S6. ORTEP representation of $[Et_3NH]_2[B_{12}H_{11}NHCO\text{-}C_6H_4\text{-}I]$; 30% displacement ellipsoids.

Crystal structure of [Et_3NH][3d-H] (CCDC1861491)

Compound [Et_3NH][**3d-H**] (10 mg) was dissolved in acetone (0.5 mL) in a 1 mL glass vial. The resulting colorless solution was filtered into a 18 cm long NMR tube and layered with hexanes (1 mL). Colorless crystals of the composition [Et_3NH] [$B_{12}H_{11}$NHC(OH)-C_6H_4-OCH_3] suitable for X-ray diffraction grew within 5 d at 25 °C.

```
Bond precision:   B- B = 0.0051 A          Wavelength=0.71073

Cell:              a=9.0952(6)      b=35.086(2)      c=14.8327(9)
                   alpha=90         beta=90          gamma=90
Temperature:       293 K

                 Calculated                  Reported
Volume           4733.3(5)                   4733.4(5)
Space group      P c c n                     P c c n
Hall group       -P 2ab 2ac                  -P 2ab 2ac
Moiety formula   C8 H20 B12 N O2, C6 H16 N   C8 H20 B12 N O2, C6 H16 N
Sum formula      C14 H36 B12 N2 O2           C14 H36 B12 N2 O2
Mr               394.17                      394.17
Dx,g cm-3        1.106                       1.106
Z                8                           8
Mu (mm-1)        0.062                       0.062
F000             1680.0                      1680.0
F000'            1680.43
h,k,lmax         10,42,17                    10,42,17
Nref             4329                        4318
Tmin,Tmax        0.981,0.989                 0.935,1.000
Tmin'            0.971

Correction method= # Reported T Limits: Tmin=0.935 Tmax=1.000
AbsCorr = MULTI-SCAN

Data completeness= 0.997             Theta(max)= 25.349

R(reflections)= 0.0927( 2910)        wR2(reflections)= 0.3020( 4318)

S = 1.042                   Npar= 366
```

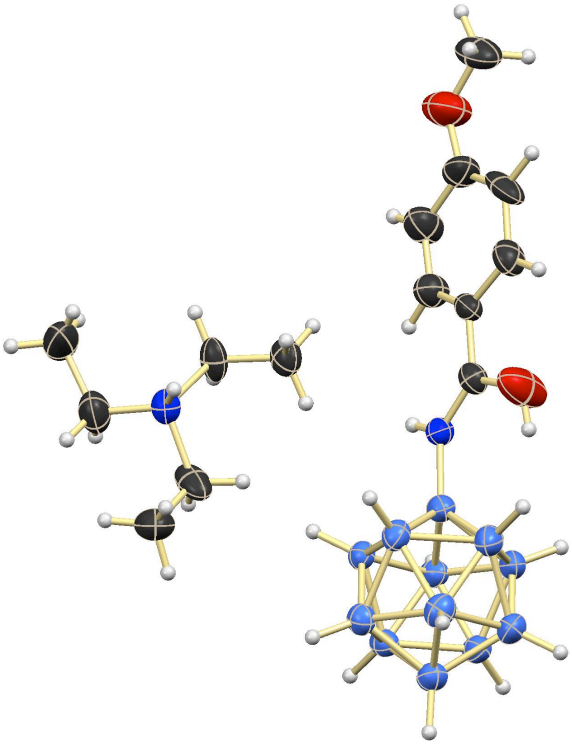

Figure S7. ORTEP representation of $[Et_3NH][B_{12}H_{11}NHC(OH)\text{-}C_6H_4\text{-}OCH_3]$; the protonated 4-methoxybenzamide moiety and the triethylammonium cation are both disordered. Only one of the two disordered parts is shown for clarity; 30% displacement ellipsoids.

Crystal structure of [Et_3NH]$_2$[3e] (CCDC1861492)

Compound [Et_3NH]$_2$[**3e**] (20 mg) was dissolved in MeCN (0.5 mL) in a 1 mL glass vial. The resulting colorless solution was filtered into an 18 cm long NMR tube and layered with Et_2O (1 mL). Colorless crystals of the composition [Et_3NH]$_2$ [$B_{12}H_{11}NHCO$-C_5H_4N] suitable for X-ray diffraction grew within 2 d at 25 °C.

```
Bond precision:   C-C = 0.0035 A          Wavelength=0.71073

Cell:             a=31.573(2)     b=10.9139(7)        c=17.2044(13)
                  alpha=90        beta=101.056(7)     gamma=90
Temperature:      293 K
```

	Calculated	Reported
Volume	5818.3(7)	5818.3(7)
Space group	C 2/c	C 1 2/c 1
Hall group	-C 2yc	-C 2yc
Moiety formula	C6 H16 B12 N2 O, 2(C6 H16 N)	C6 H16 B12 N2 O, 2(C6 H16 N)
Sum formula	C18 H48 B12 N4 O	C18 H48 B12 N4 O
Mr	466.32	466.32
Dx,g cm-3	1.065	1.065
Z	8	8
Mu (mm-1)	0.059	0.059
F000	2016.0	2016.0
F000’	2016.43	
h,k,lmax	38,13,20	38,13,20
Nref	5345	5342
Tmin,Tmax	0.972,0.977	0.949,1.000
Tmin’	0.972	

```
Correction method= # Reported T Limits: Tmin=0.949 Tmax=1.000
AbsCorr = MULTI-SCAN

Data completeness= 0.999          Theta(max)= 25.350

R(reflections)= 0.0605( 3486)     wR2(reflections)= 0.1775( 5342)

S = 1.050                  Npar= 350
```

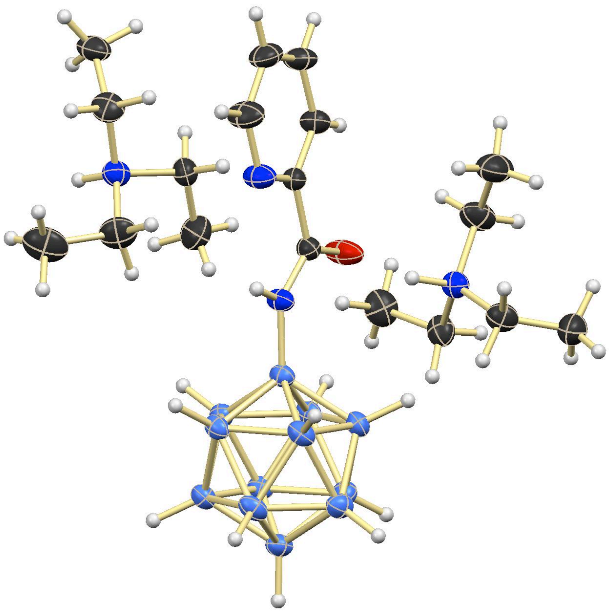

Figure S8. ORTEP representation of $[Et_3NH]_2[B_{12}H_{11}NHCO\text{-}C_5H_4N]$; 30% displacement ellipsoids.

Crystal structure of 3e-H (CCDC1861490)

Compound [Et_3NH][**3e-H**] (25 mg) was dissolved in MeOH/MeCN (1 mL/1 mL) at *ca.* 50 °C in a 4 mL glass vial and allowed to cool to room temperature. Colorless crystals of the composition [Et_3NH][$B_{12}H_{11}NHCO-C_5H_4N-H$]·$CH_3CN$ suitable for X-ray diffraction were obtained within 1 d.

```
Bond precision:   C-C = 0.0041 A          Wavelength=0.71073

Cell:             a=12.4068(12)    b=15.2490(18)       c=13.0624(12)
                  alpha=90         beta=102.374(10)    gamma=90
Temperature:      293 K

                   Calculated                    Reported
Volume             2413.9(4)                     2413.9(4)
Space group        P 21/c                        P 1 21/c 1
Hall group         -P 2ybc                       -P 2ybc
Moiety formula     C6 H17 B12 N2 O, C6 H16 N,    C6 H17 B12 N2 O, C6 H16 N,
                   C2 H3 N                       C2 H3 N
Sum formula        C14 H36 B12 N4 O              C14 H36 B12 N4 O
Mr                 406.19                        406.19
Dx,g cm-3          1.118                         1.118
Z                  4                             4
Mu (mm-1)          0.062                         0.062
F000               864.0                         864.0
F000'              864.18
h,k,lmax           14,18,15                      14,18,15
Nref               4420                          4405
Tmin,Tmax          0.976,0.982                   0.948,1.000
Tmin'              0.976

Correction method= # Reported T Limits: Tmin=0.948 Tmax=1.000
AbsCorr = MULTI-SCAN

Data completeness= 0.997           Theta(max)= 25.348

R(reflections)= 0.0654( 2790)      wR2(reflections)= 0.1858( 4405)

S = 1.025                     Npar= 288
```

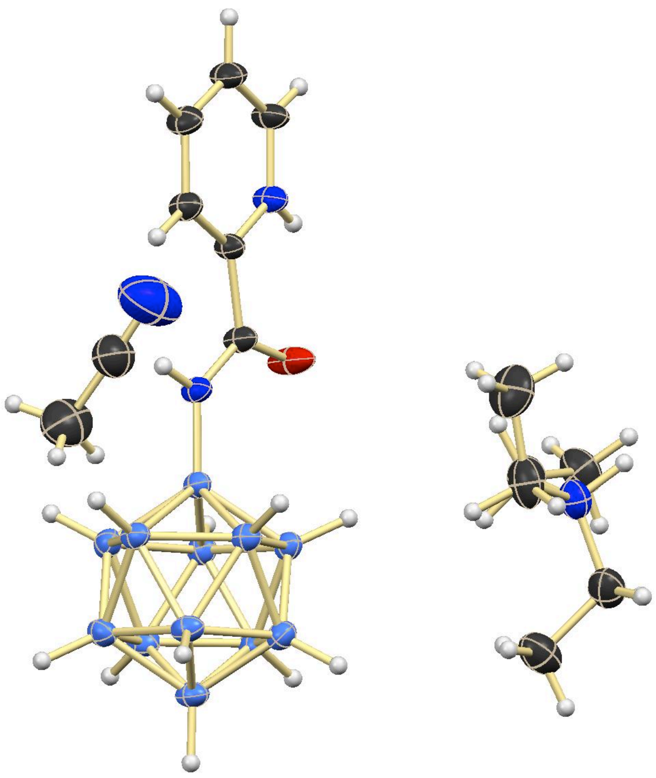

Figure S9. ORTEP representation of $[Et_3NH][B_{12}H_{11}NHCO\text{-}C_5H_4N\text{-}H]\cdot CH_3CN$; 30% displacement ellipsoids.

Crystal structure of [Et_3NH][6a] (CCDC1861483)

Compound [Et_3NH][**6a**] (10 mg, 0.031 mmol) was dissolved in acetonitrile (0.5 mL) in a 1 mL glass vial. The resulting colorless solution was filtered into a 18 cm long NMR tube and layered with diethylether (1 mL). Colorless crystals of the composition [Et_3NH][$B_{12}H_{11}NH{=}CH{-}N(CH_3)_2$]·$2CH_3CN$ suitable for X-ray diffraction grew within 5 d at 25 °C.

Bond precision: C-C = 0.0028 A Wavelength=0.71073

Cell:	a=8.7477(5)	b=21.7928(15)	c=13.5423(8)
	alpha=90	beta=97.268(5)	gamma=90
Temperature:	170 K		

	Calculated	Reported
Volume	2560.9(3)	2560.9(3)
Space group	P 21/n	P 1 21/n 1
Hall group	-P 2yn	-P 2yn
Moiety formula	C3 H19 B12 N2, C6 H16 N, 2(C2 H3 N)	C3 H19 B12 N2, C6 H16 N, 2(C2 H3 N)
Sum formula	C13 H41 B12 N5	C13 H41 B12 N5
Mr	397.23	397.23
Dx,g cm-3	1.030	1.030
Z	4	4
Mu (mm-1)	0.055	0.055
F000	856.0	856.0
F000'	856.13	
h,k,lmax	10,26,16	10,26,16
Nref	4700	4689
Tmin,Tmax	0.982,0.986	0.917,1.000
Tmin'	0.979	

Correction method= # Reported T Limits: Tmin=0.917 Tmax=1.000
AbsCorr = MULTI-SCAN

Data completeness= 0.998 Theta(max)= 25.350

R(reflections)= 0.0464(3655) wR2(reflections)= 0.1289(4689)

S = 1.028 Npar= 278

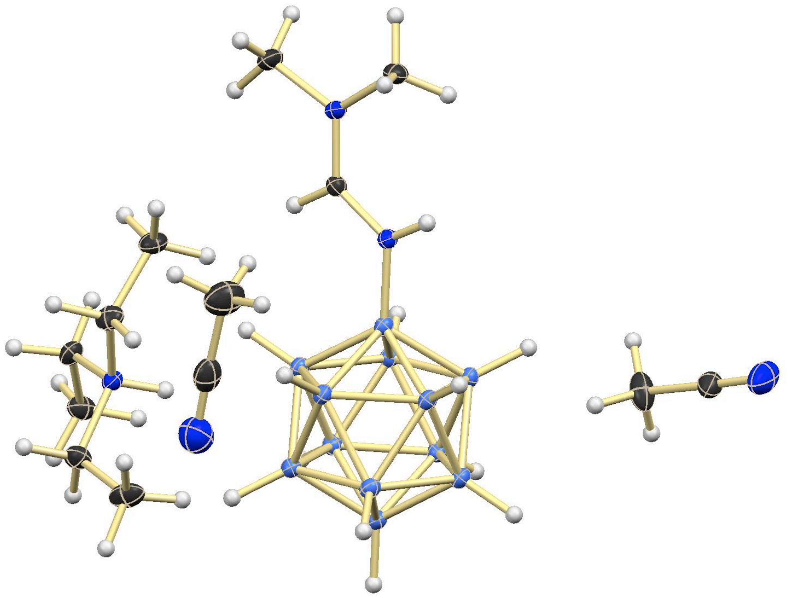

Figure S10. ORTEP representation of [Et_3NH][$B_{12}H_{11}NH{=}CH{-}N(CH_3)_2$]·2$CH_3CN$; 30% displacement ellipsoids.

Crystal structure of [MePPh$_3$][6a] (CCDC1861484)

Single crystals of **6a** were also obtained with the [MePPh$_3$]$^+$ cation, and the structure is similar to that of [Et$_3$NH][**6a**]. [Et$_3$NH][**6a**] (30 mg) was suspended in water (1 mL), and NaOH (2 equiv) was added to form the Na$^+$ salt. To this solution [MePPh$_3$]Br (2 equiv) was added to give [MePPh$_3$][**6a**] as a colorless precipitate. [MePPh$_3$][**6a**] (20 mg) was dissolved in acetone (0.5 mL). The resulting colorless solution was filtered into an 18 cm long NMR tube and layered with Et$_2$O (1 mL). Colorless crystals of the composition [MePPh$_3$] [$B_{12}H_{11}$NH=CH–N(CH$_3$)$_2$] suitable for X-ray diffraction grew within 2 d at 25 °C.

```
Bond precision:   C-C = 0.0045 A          Wavelength=0.71073

Cell:             a=13.1538(7)     b=20.5673(9)        c=11.3688(6)
                  alpha=90         beta=103.263(5)     gamma=90
Temperature:      293 K

                  Calculated                   Reported
Volume            2993.7(3)                    2993.7(3)
Space group       P 21/c                       P 1 21/c 1
Hall group        -P 2ybc                      -P 2ybc
Moiety formula    C19 H18 P, C3 H19 B12 N2     C19 H18 P, C3 H19 B12 N2
Sum formula       C22 H37 B12 N2 P             C22 H37 B12 N2 P
Mr                490.23                       490.23
Dx,g cm-3         1.088                        1.088
Z                 4                            4
Mu (mm-1)         0.108                        0.108
F000              1032.0                       1032.0
F000'             1032.62
h,k,lmax          15,24,13                     15,24,13
Nref              5485                         5453
Tmin,Tmax         0.948,0.958                  0.982,1.000
Tmin'             0.948

Correction method= # Reported T Limits: Tmin=0.982 Tmax=1.000
AbsCorr = MULTI-SCAN

Data completeness= 0.994          Theta(max)= 25.350

R(reflections)= 0.0587( 3574)     wR2(reflections)= 0.1595( 5453)

S = 1.033                    Npar= 337
```

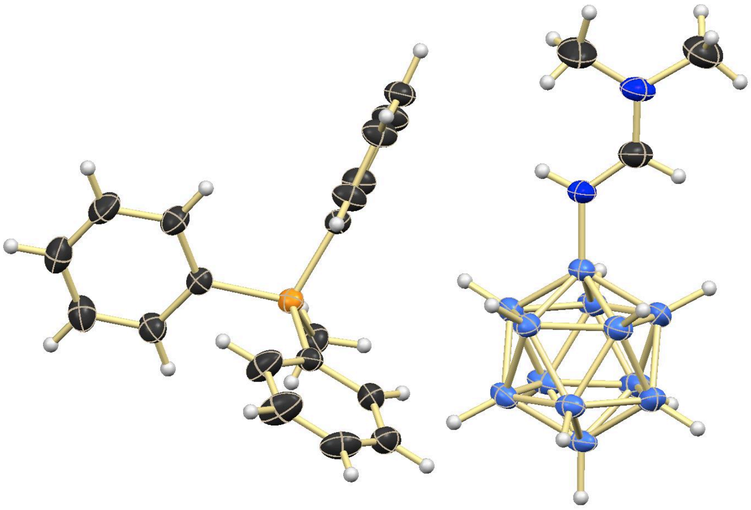

Figure S11. ORTEP representation of [$MePPh_3$][$B_{12}H_{11}NH{=}CH{-}N(CH_3)_2$]; 30% displacement ellipsoids.

Crystal structure of [Et_3NH][6c] (CCDC1861485)

Compound [Et_3NH][**6c**] (10 mg) was dissolved in acetonitrile (0.5 mL) in a 1 mL glass vial. The resulting colorless solution was filtered into a 18 cm long NMR tube and layered with diethylether (1 mL). Colorless crystals of the composition [Et_3NH][$B_{12}H_{11}NH{=}C(C_6H_5)(NH\text{-}C_6H_5)$]·$H_2O$ suitable for X-ray diffraction grew within 5 d at 25 °C.

```
Bond precision:   C-C = 0.0059 A          Wavelength=0.71073

Cell:            a=10.8688(7)       b=12.0426(6)      c=13.1037(9)
                 alpha=66.402(5)    beta=67.187(6)    gamma=85.080(5)
Temperature:     293 K
```

	Calculated	Reported
Volume	1443.85(18)	1443.85(15)
Space group	P -1	P -1
Hall group	-P 1	-P 1
Moiety formula	C13 H23 B12 N2, C6 H16 N, H2 O	C13 H23 B12 N2, C6 H16 N, H2 O
Sum formula	C19 H41 B12 N3 O	C19 H41 B12 N3 O
Mr	457.27	457.27
Dx,g cm-3	1.052	1.052
Z	2	2
Mu (mm-1)	0.057	0.057
F000	488.0	488.0
F000'	488.11	
h,k,lmax	13,14,15	13,14,15
Nref	5276	5202
Tmin,Tmax	0.976,0.980	0.985,1.000
Tmin'	0.976	

```
Correction method= # Reported T Limits: Tmin=0.985 Tmax=1.000
AbsCorr = MULTI-SCAN

Data completeness= 0.986          Theta(max)= 25.350

R(reflections)= 0.0962( 3419)     wR2(reflections)= 0.3064( 5202)

S = 1.050                 Npar= 329
```

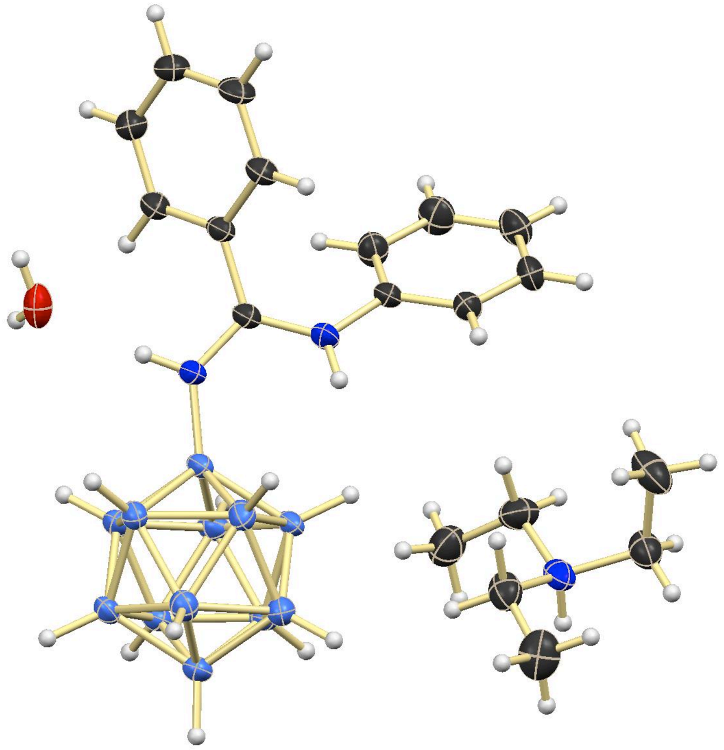

Figure S12. ORTEP representation of $[B_{12}H_{11}NH{=}C(C_6H_5)(NH\text{-}C_6H_5)]\cdot H_2O$; 30% displacement ellipsoids.

Crystal structure of [$MePPh_3$]$_2$[8] (CCDC1861487)

[$MePPh_3$]$_2$[**8**] **(**10 mg) was dissolved in acetone (0.5 mL) in a 1 mL glass vial. The resulting colorless solution was filtered into an 18 cm long NMR tube and layered with Et_2O (1 mL). Colorless crystals of the composition [$MePPh_3$]$_2$[$B_{12}H_{11}N{=}C{=}O$] suitable for X-ray diffraction grew within 2 d at 25 °C. Single crystals could also be obtained by recrystallization from aceotone.

```
Bond precision:   C-C = 0.0041 A          Wavelength=0.71073

Cell:           a=11.3939(14)     b=13.1505(15)    c=14.8700(15)
                alpha=89.844(9)   beta=81.969(9)   gamma=71.540(11)
Temperature:    293 K
```

	Calculated	Reported
Volume	2090.6(4)	2090.6(4)
Space group	P -1	P -1
Hall group	-P 1	-P 1
Moiety formula	2(C19 H18 P), C H11 B12 N O	2(C19 H18 P), C H11 B12 N O
Sum formula	C39 H47 B12 N O P2	C39 H47 B12 N O P2
Mr	737.44	737.44
Dx,g cm-3	1.171	1.171
Z	2	2
Mu (mm-1)	0.137	0.137
F000	772.0	772.0
F000'	772.62	
h,k,lmax	13,15,17	13,15,17
Nref	7655	7633
Tmin,Tmax	0.952,0.973	0.575,1.000
Tmin'	0.952	

```
Correction method= # Reported T Limits: Tmin=0.575 Tmax=1.000
AbsCorr = MULTI-SCAN

Data completeness= 0.997          Theta(max)= 25.350

R(reflections)= 0.0507( 4888)     wR2(reflections)= 0.1366( 7633)

S = 0.961                  Npar= 498
```

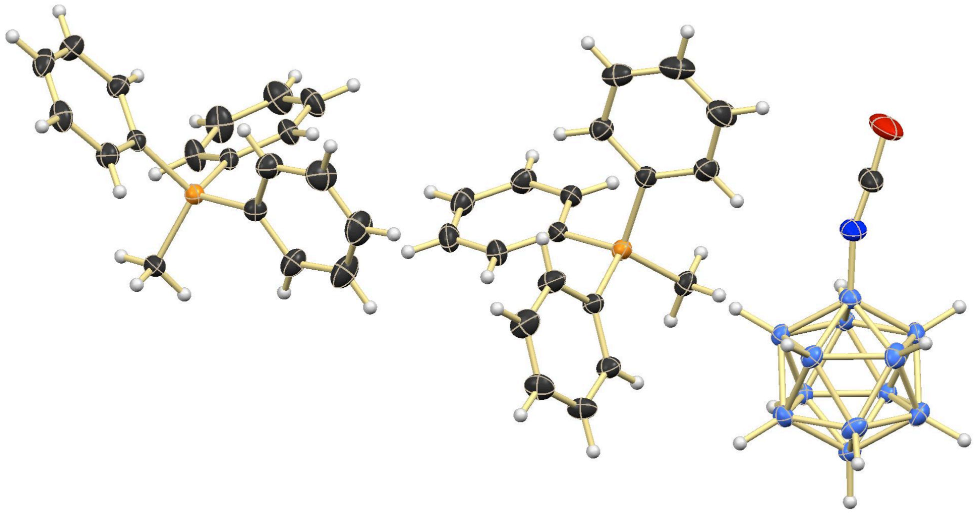

Figure S13. ORTEP representation of $[MePPh_3]_2[B_{12}H_{11}N{=}C{=}O]$; 30% displacement ellipsoids.

IV References

[1] V. Geis, K. Guttsche, C. Knapp, H. Scherer, R. Uzun, *Dalton Trans.* **2009**, 2687–2694.

[2] O. Bondarev, A. A. Khan, X. Tu, Y. V. Sevrugina, S. S. Jalisatgi, M. F. Hawthorne, *J. Am. Chem. Soc*. **2013**, *135*, 13204–13211.

[3] Y. Sun, J. Zhang, Y. Zhang, J. Liu, S. van der Veen, S. Duttwyler, *Chem. Eur. J.* **2018**, *24*, 10364–10371.

1H{11B} NMR 400MHz CD3CN

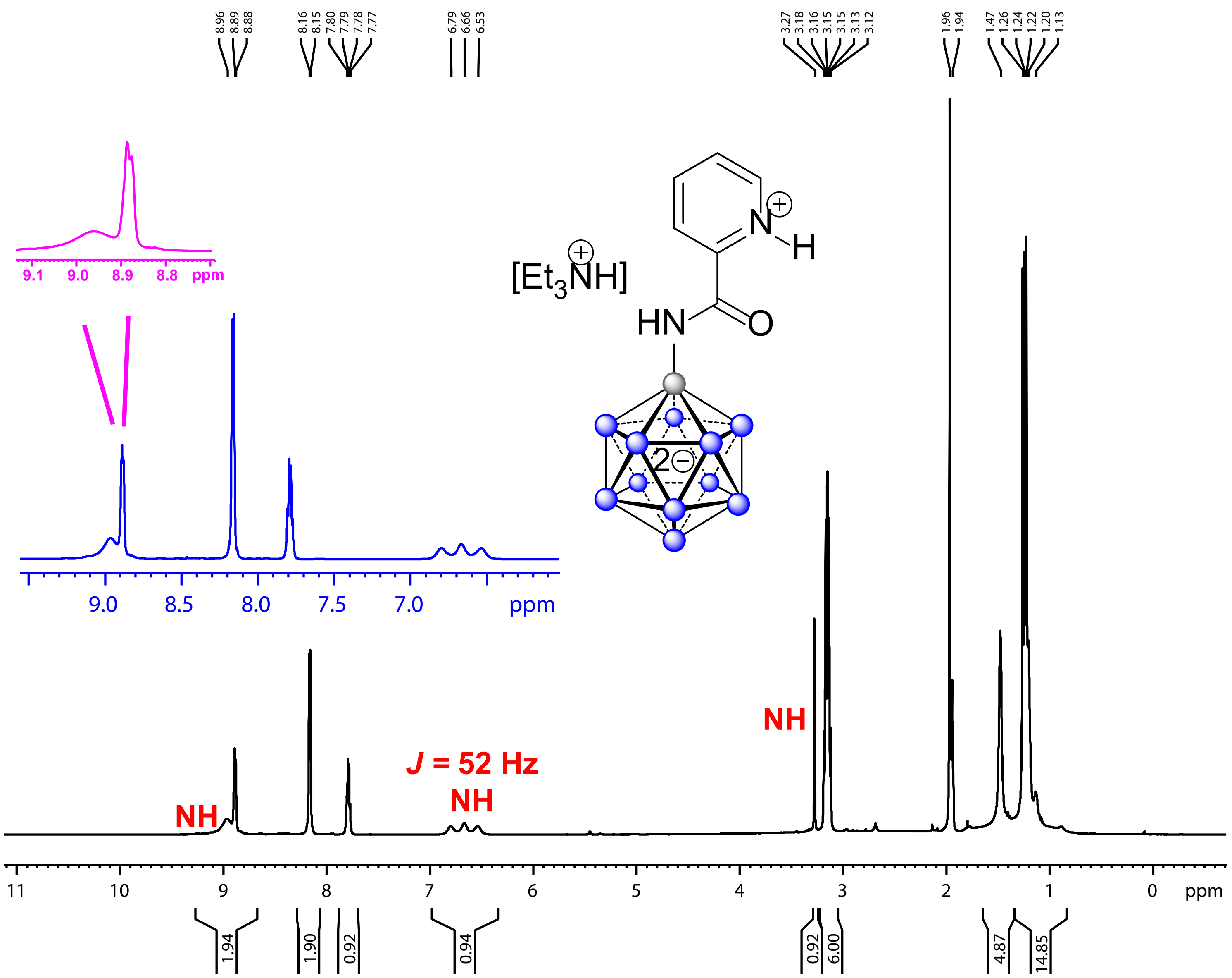


```
Current Data Parameters
NAME      20180517_early_wt-01071
EXPNO                     2
PROCNO                    1

F2 - Acquisition Parameters
Date_              20171213
Time                  20.20
INSTRUM               spect
PROBHD   5 mm PABBO BB/
PULPROG              zgig30
TD                    16384
SOLVENT               CD3CN
NS                       64
DS                        4
SWH               8012.820 Hz
FIDRES            0.489064 Hz
AQ               1.0223616 sec
RG                    86.58
DW                   62.400 usec
DE                     6.50 usec
TE                    294.0 K
D1              1.00000000 sec
D11             0.03000000 sec
TD0                       1

======== CHANNEL f1 ========
NUC1                     1H
P1                    15.00 usec
PLW1            12.50000000 W
SFO1            400.1320007 MHz

======== CHANNEL f2 ========
CPDPRG[2              garp4
NUC2                    11B
PCPD2                 90.00 usec
PLW2            52.96599960 W
PLW12            0.64477998 W
SFO2            128.3776050 MHz

F2 - Processing parameters
SI                    32768
SF              400.1300118 MHz
WDW                      EM
SSB      0
LB                     1.00 Hz
GB       0
PC                     1.40
```

13C{1H} NMR 101MHz CD3CN

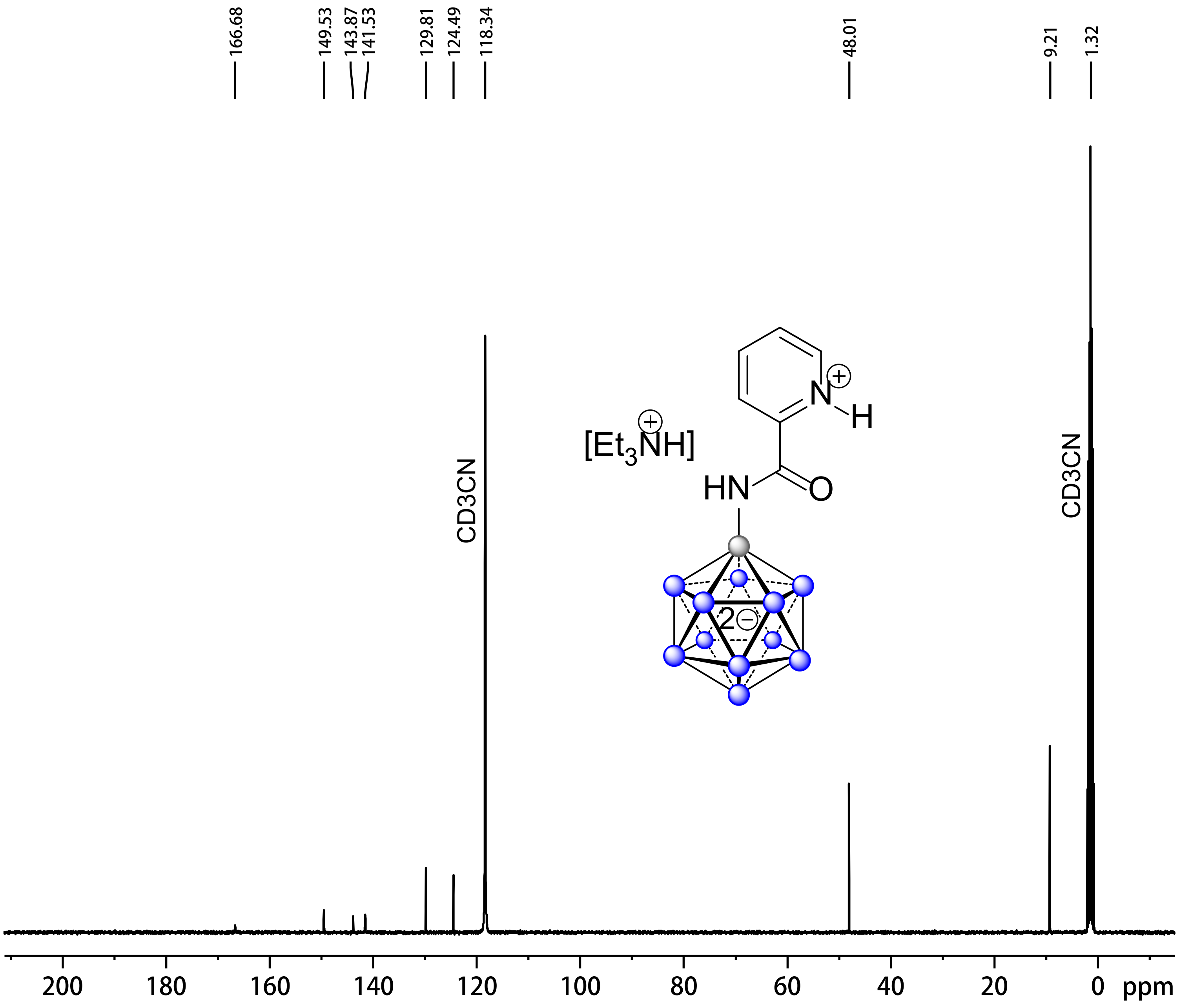


```
Current Data Parameters
NAME     wt-01071_crst_09th_rt
EXPNO                   5
PROCNO                  1

F2 - Acquisition Parameters
Date_           20180517
Time               14.33
INSTRUM            spect
PROBHD    5 mm PABBO BB/
PULPROG          zgpg30
TD                65536
SOLVENT           CD3CN
NS                 2048
DS                    4
SWH          29761.904 Hz
FIDRES        0.454131 Hz
AQ           1.1010048 sec
RG              193.34
DW              16.800 usec
DE                6.50 usec
TE               294.9 K
D1          1.50000000 sec
D11         0.03000000 sec
TD0                  1

======== CHANNEL f1 ========
NUC1                 13C
P1                 10.00 usec
PLW1         53.00000000 W
SFO1         100.6228293 MHz

======== CHANNEL f2 ========
CPDPRG[2         waltz16
NUC2                  1H
PCPD2              80.00 usec
PLW2         12.50000000 W
PLW12         0.43945000 W
PLW13         0.28125000 W
SFO2         400.1316005 MHz

F2 - Processing parameters
SI                 32768
SF           100.6126739 MHz
WDW                   EM
SSB      0
LB                  1.00 Hz
GB       0
PC                  1.40
```

11B NMR 126 MHz CD3CN

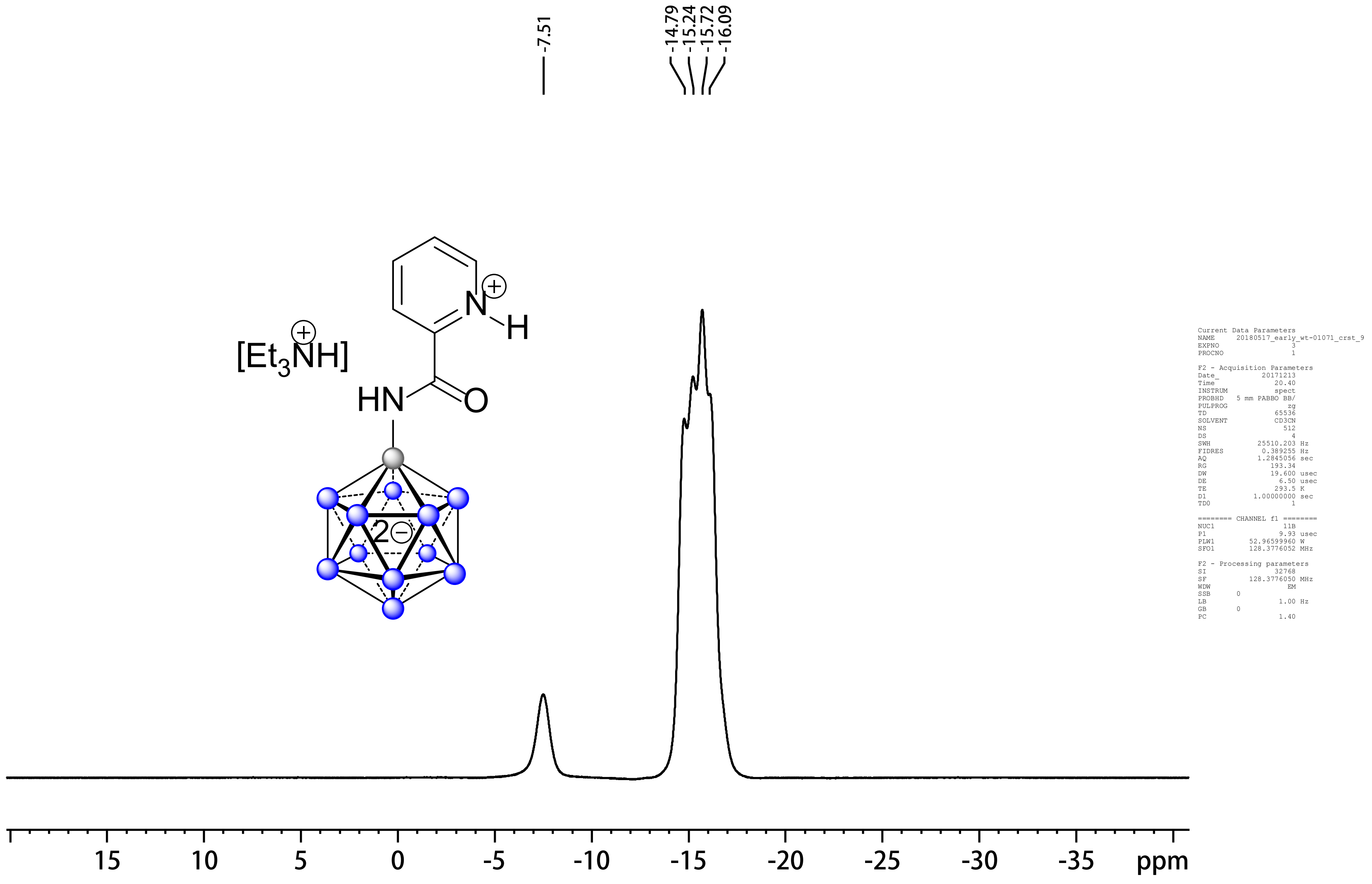

11B{1H} NMR 126 MHz CD3CN

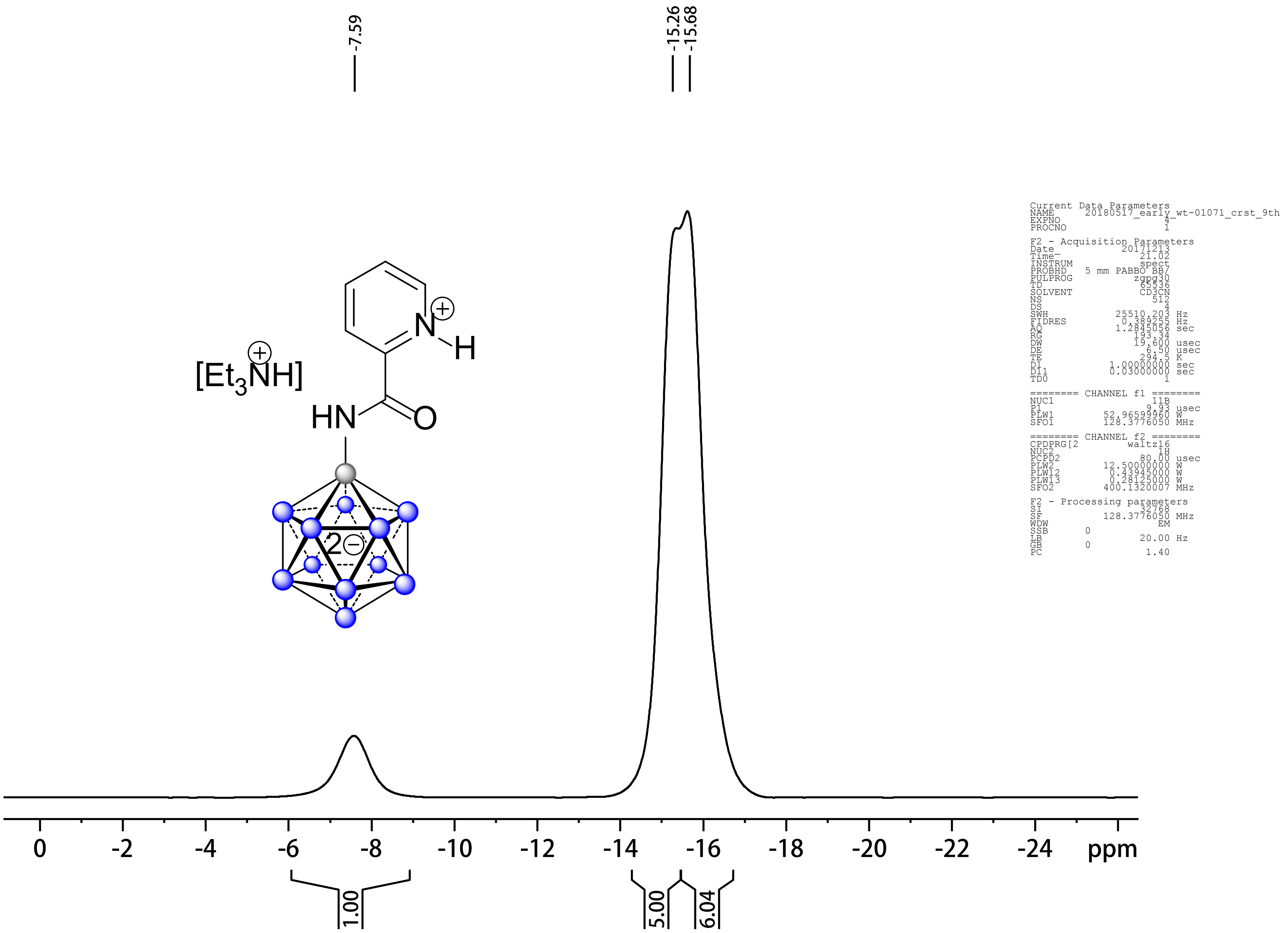

1H{11B} NMR 400MHz CD3CN

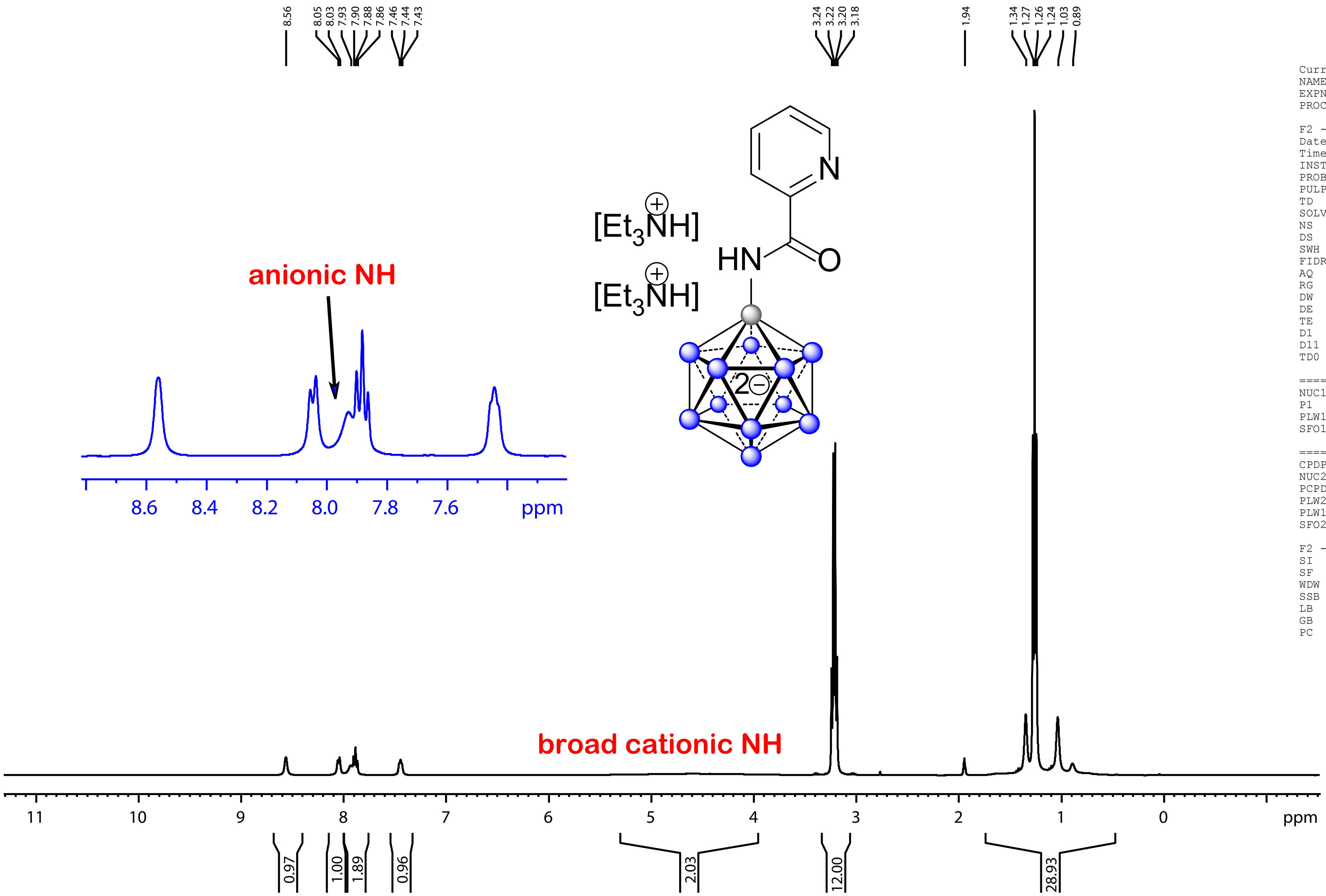


```
Current Data Parameters
NAME       wt-01078b
EXPNO              2
PROCNO             1

F2 - Acquisition Parameters
Date_          20180519
Time               1.37
INSTRUM           spect
PROBHD   5 mm PABBO BB/
PULPROG         zgig30
TD               16384
SOLVENT          CD3CN
NS                  16
DS                   4
SWH           8012.820 Hz
FIDRES        0.489064 Hz
AQ           1.0223616 sec
RG               55.74
DW              62.400 usec
DE                6.50 usec
TE               294.5 K
D1          1.00000000 sec
D11         0.03000000 sec
TD0                  1

======== CHANNEL f1 ========
NUC1                1H
P1               15.00 usec
PLW1       12.50000000 W
SFO1       400.1320007 MHz

======== CHANNEL f2 ========
CPDPRG[2         garp4
NUC2               11B
PCPD2            90.00 usec
PLW2       52.96599960 W
PLW12       0.64477998 W
SFO2       128.3776050 MHz

F2 - Processing parameters
SI               32768
SF         400.1300115 MHz
WDW                 EM
SSB      0
LB                1.00 Hz
GB       0
PC                1.40
```

13C{1H} NMR 101MHz CD3CN

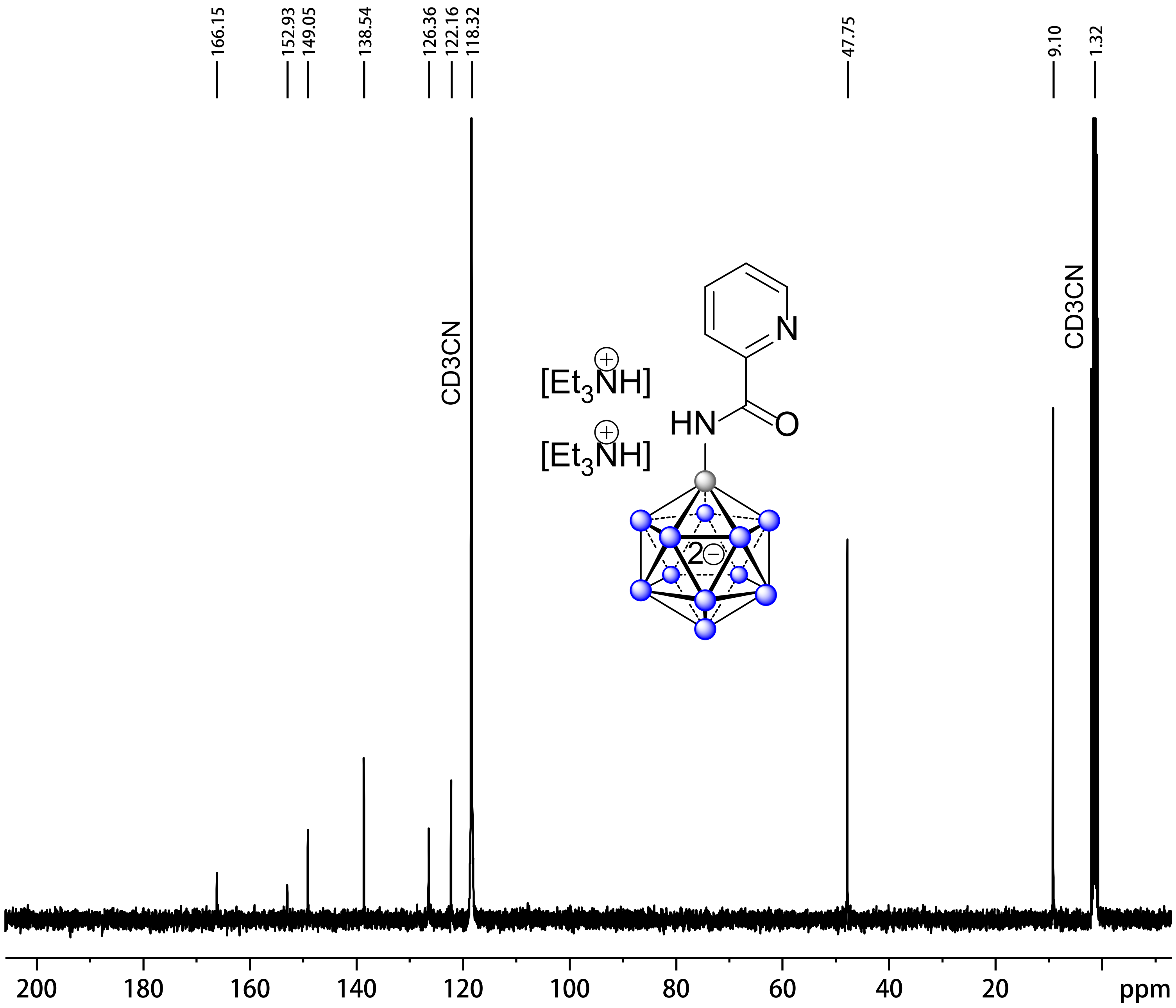


```
Current Data Parameters
NAME      wt-01078b
EXPNO                 5
PROCNO                1

F2 - Acquisition Parameters
Date_          20180519
Time               2.13
INSTRUM           spect
PROBHD   5 mm PABBO BB/
PULPROG         zgpg30
TD               65536
SOLVENT          CD3CN
NS                 512
DS                   4
SWH          29761.904 Hz
FIDRES        0.454131 Hz
AQ           1.1010048 sec
RG              193.34
DW              16.800 usec
DE                6.50 usec
TE               295.5 K
D1          1.50000000 sec
D11         0.03000000 sec
TD0                  1

======== CHANNEL f1 ========
NUC1               13C
P1               10.00 usec
PLW1       53.00000000 W
SFO1       100.6228293 MHz

======== CHANNEL f2 ========
CPDPRG[2       waltz16
NUC2                1H
PCPD2            80.00 usec
PLW2       12.50000000 W
PLW12       0.43945000 W
PLW13       0.28125000 W
SFO2       400.1316005 MHz

F2 - Processing parameters
SI               32768
SF         100.6126762 MHz
WDW                 EM
SSB       0
LB                1.00 Hz
GB        0
PC                1.40
```

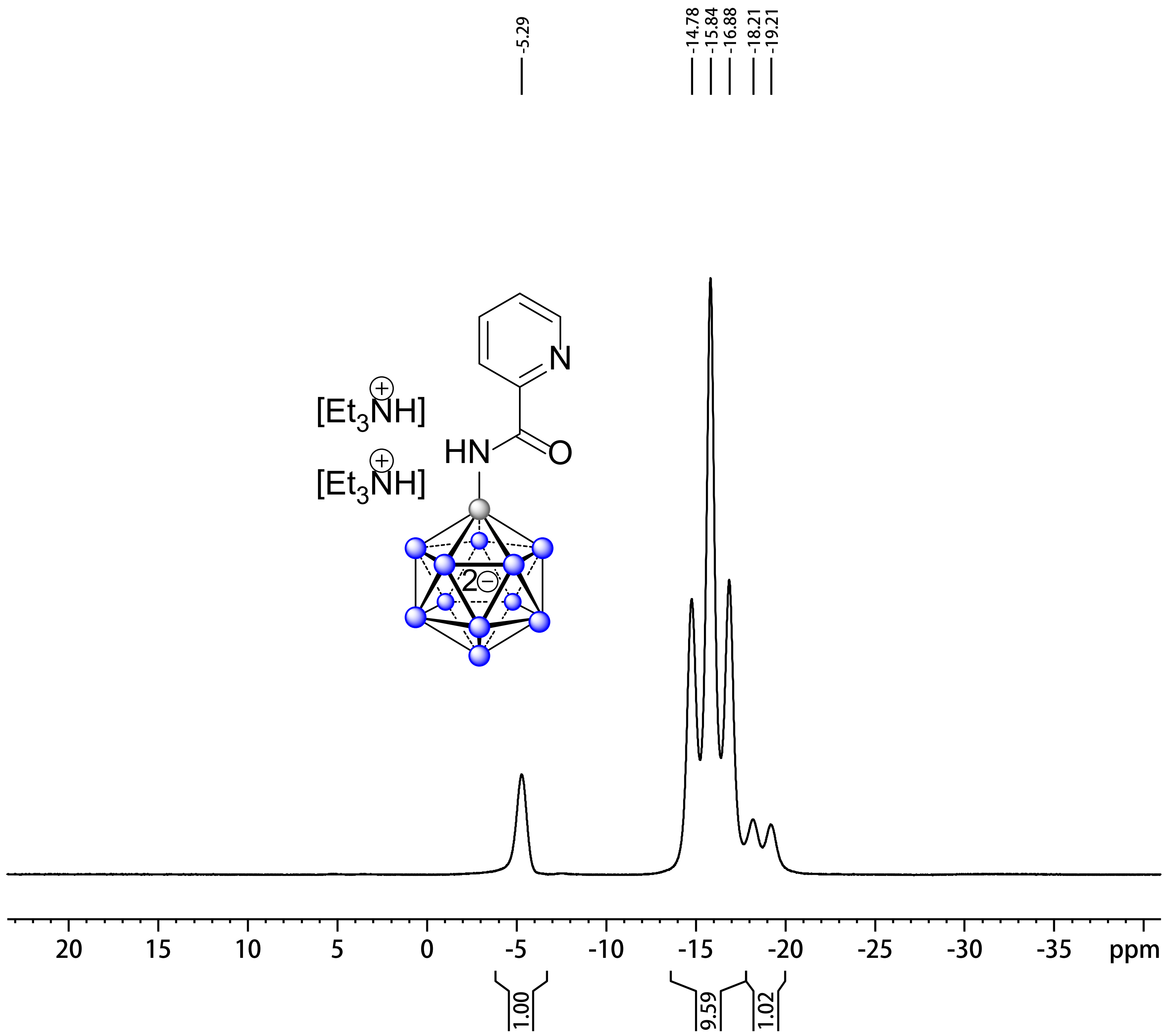


```
Current Data Parameters
NAME     wt-01078b-[NEt3H]2[B12H11NHCOPy]
EXPNO                  3
PROCNO                 1

F2 - Acquisition Parameters
Date_          20180519
Time               1.43
INSTRUM           spect
PROBHD   5 mm PABBO BB/
PULPROG              zg
TD                65536
SOLVENT           CD3CN
NS                  128
DS                    4
SWH           25510.203 Hz
FIDRES         0.389255 Hz
AQ            1.2845056 sec
RG               193.34
DW               19.600 usec
DE                 6.50 usec
TE                294.5 K
D1           1.00000000 sec
TD0                   1

======== CHANNEL f1 ========
NUC1                11B
P1                 9.93 usec
PLW1        52.96599960 W
SFO1        128.3776052 MHz

F2 - Processing parameters
SI                32768
SF          128.3776050 MHz
WDW                  EM
SSB      0
LB                 1.00 Hz
GB       0
PC                 1.40
```

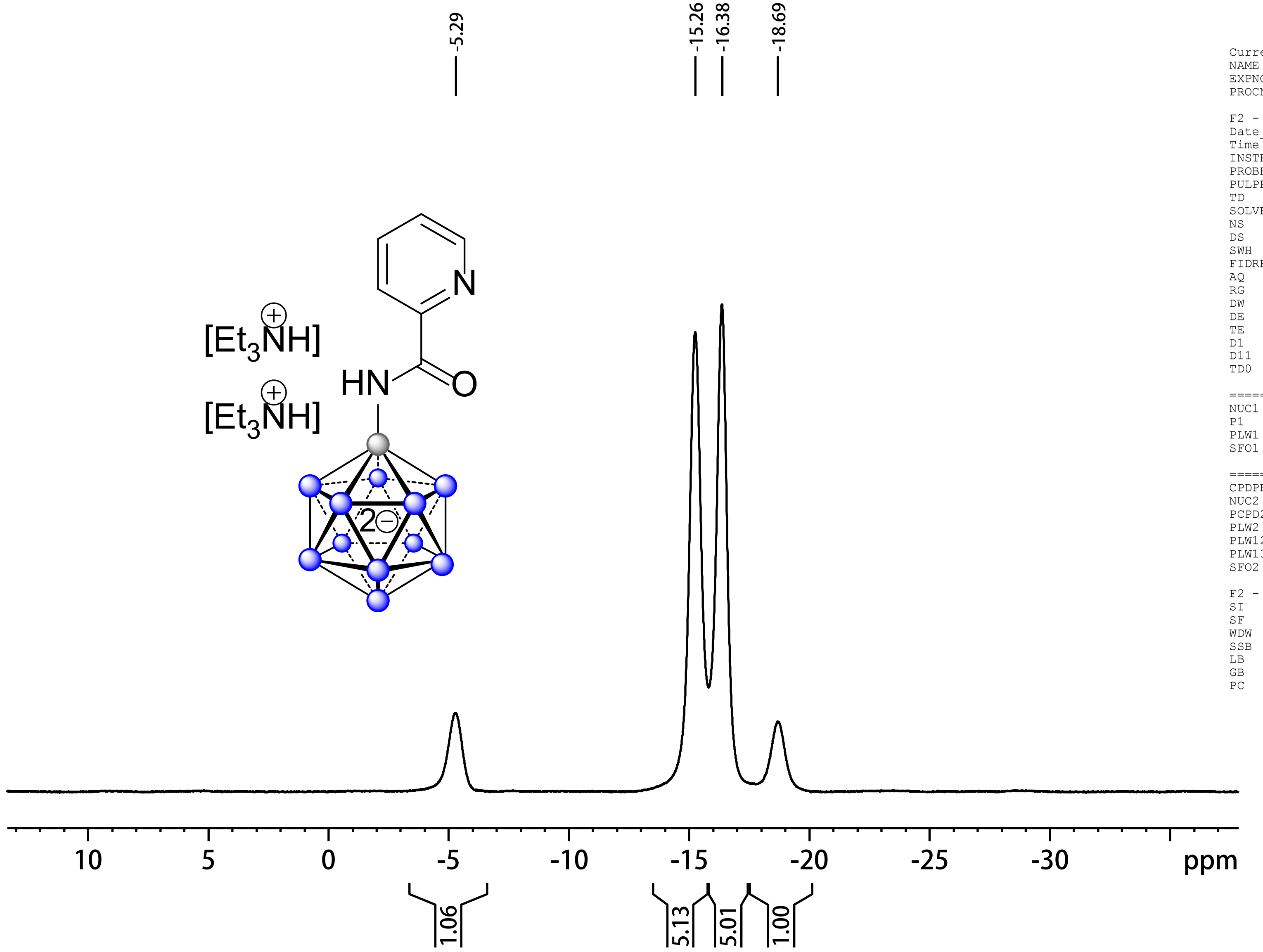


```
Current Data Parameters
NAME     wt-01078b-[NEt3H]2[B12H11NHCOPy]
EXPNO                 4
PROCNO                1

F2 - Acquisition Parameters
Date_          20180519
Time               1.49
INSTRUM           spect
PROBHD   5 mm PABBO BB/
PULPROG         zgpg30
TD               65536
SOLVENT           CD3CN
NS                  128
DS                    4
SWH           25510.203 Hz
FIDRES         0.389255 Hz
AQ            1.2845056 sec
RG               193.34
DW               19.600 usec
DE                 6.50 usec
TE                295.1 K
D1           1.00000000 sec
D11          0.03000000 sec
TD0                   1

======== CHANNEL f1 ========
NUC1                11B
P1                 9.93 usec
PLW1        52.96599960 W
SFO1        128.3776050 MHz

======== CHANNEL f2 ========
CPDPRG[2        waltz16
NUC2                 1H
PCPD2             80.00 usec
PLW2        12.50000000 W
PLW12        0.43945000 W
PLW13        0.28125000 W
SFO2        400.1320007 MHz

F2 - Processing parameters
SI                32768
SF          128.3776050 MHz
WDW                  EM
SSB      0
LB                 1.00 Hz
GB       0
PC                 1.40
```

20180602 [NBu4]2[B12H11NHCONHPh] 40mg dissolved in CD3CN
1H{11B} NMR 400 MHz

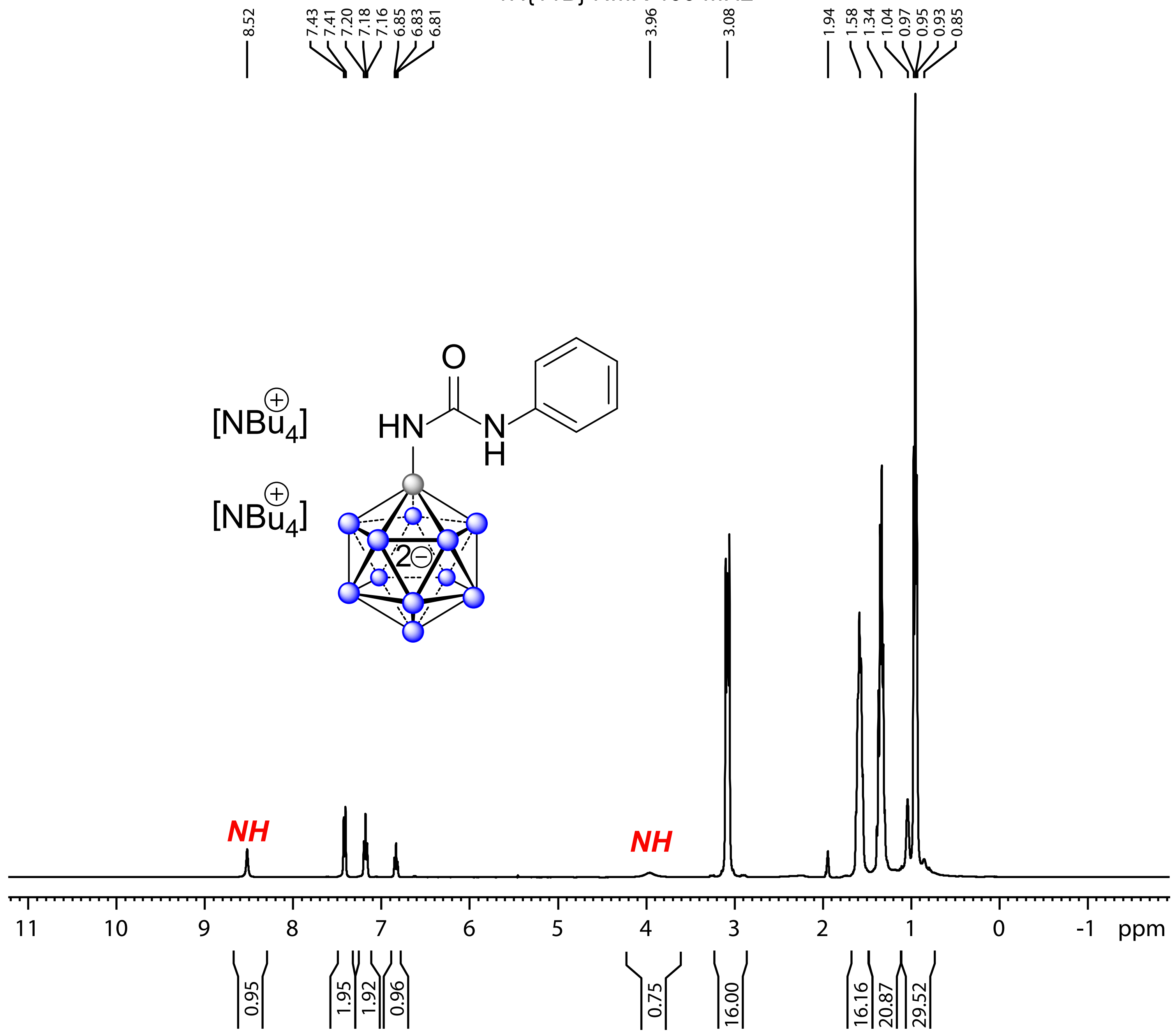


```
Current Data Parameters
NAME      20180602-B12H11NHCONHPh
EXPNO                  2
PROCNO                 1

F2 - Acquisition Parameters
Date_           20180603
Time                3.44
INSTRUM            spect
PROBHD    5 mm PABBO BB/
PULPROG          zgig30
TD                16384
SOLVENT           CD3CN
NS                   16
DS                    4
SWH            8012.820 Hz
FIDRES         0.489064 Hz
AQ            1.0223616 sec
RG                23.04
DW               62.400 usec
DE                 6.50 usec
TE                293.5 K
D1           1.00000000 sec
D11          0.03000000 sec
TD0                   1

======== CHANNEL f1 ========
NUC1                 1H
P1                15.00 usec
PLW1        12.50000000 W
SFO1        400.1320007 MHz

======== CHANNEL f2 ========
CPDPRG[2          garp4
NUC2                11B
PCPD2             90.00 usec
PLW2        52.96599960 W
PLW12        0.64477998 W
SFO2        128.3776050 MHz

F2 - Processing parameters
SI                32768
SF          400.1300118 MHz
WDW                  EM
SSB      0
LB                 1.00 Hz
GB       0
PC                 1.40
```

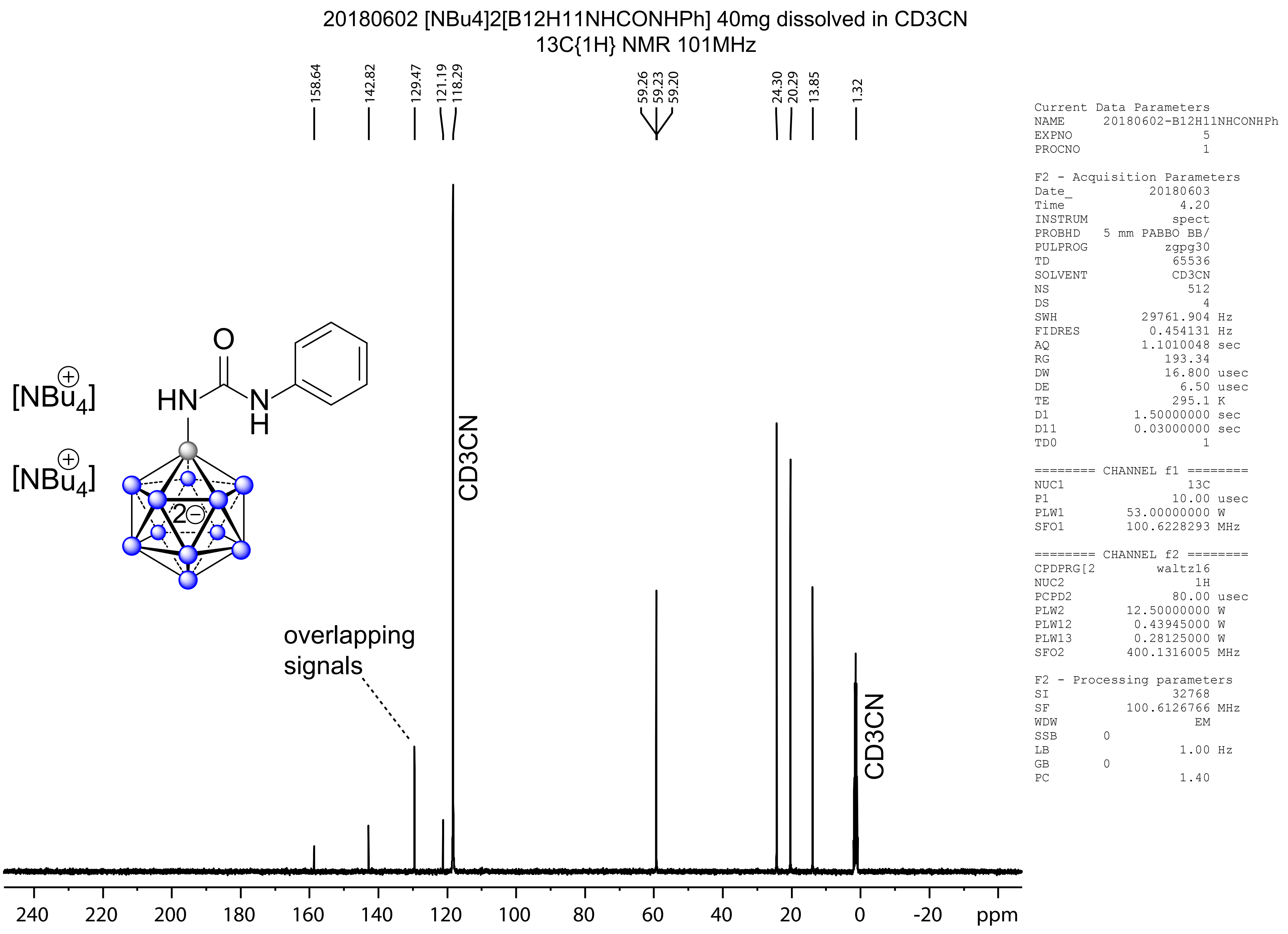
20180602 [NBu4]2[B12H11NHCONHPh] 40mg dissolved in CD3CN
13C{1H} NMR 101MHz
158.64
142.82
129.47
121.19
118.29
59.26
59.23
59.20
24.30
20.29
13.85
1.32
[NBu4]
[NBu4]
HN
O
N
H
2⊖
CD3CN
overlapping signals
CD3CN
240
220
200
180
160
140
120
100
80
60
40
20
0
-20
ppm
Current Data Parameters
NAME 20180602-B12H11NHCONHPh
EXPNO 5
PROCNO 1
F2 - Acquisition Parameters
Date_ 20180603
Time 4.20
INSTRUM spect
PROBHD 5 mm PABBO BB/
PULPROG zgpg30
TD 65536
SOLVENT CD3CN
NS 512
DS 4
SWH 29761.904 Hz
FIDRES 0.454131 Hz
AQ 1.1010048 sec
RG 193.34
DW 16.800 usec
DE 6.50 usec
TE 295.1 K
D1 1.50000000 sec
D11 0.03000000 sec
TD0 1
======== CHANNEL f1 ========
NUC1 13C
P1 10.00 usec
PLW1 53.00000000 W
SFO1 100.6228293 MHz
======== CHANNEL f2 ========
CPDPRG[2 waltz16
NUC2 1H
PCPD2 80.00 usec
PLW2 12.50000000 W
PLW12 0.43945000 W
PLW13 0.28125000 W
SFO2 400.1316005 MHz
F2 - Processing parameters
SI 32768
SF 100.6126766 MHz
WDW EM
SSB 0
LB 1.00 Hz
GB 0
PC 1.40

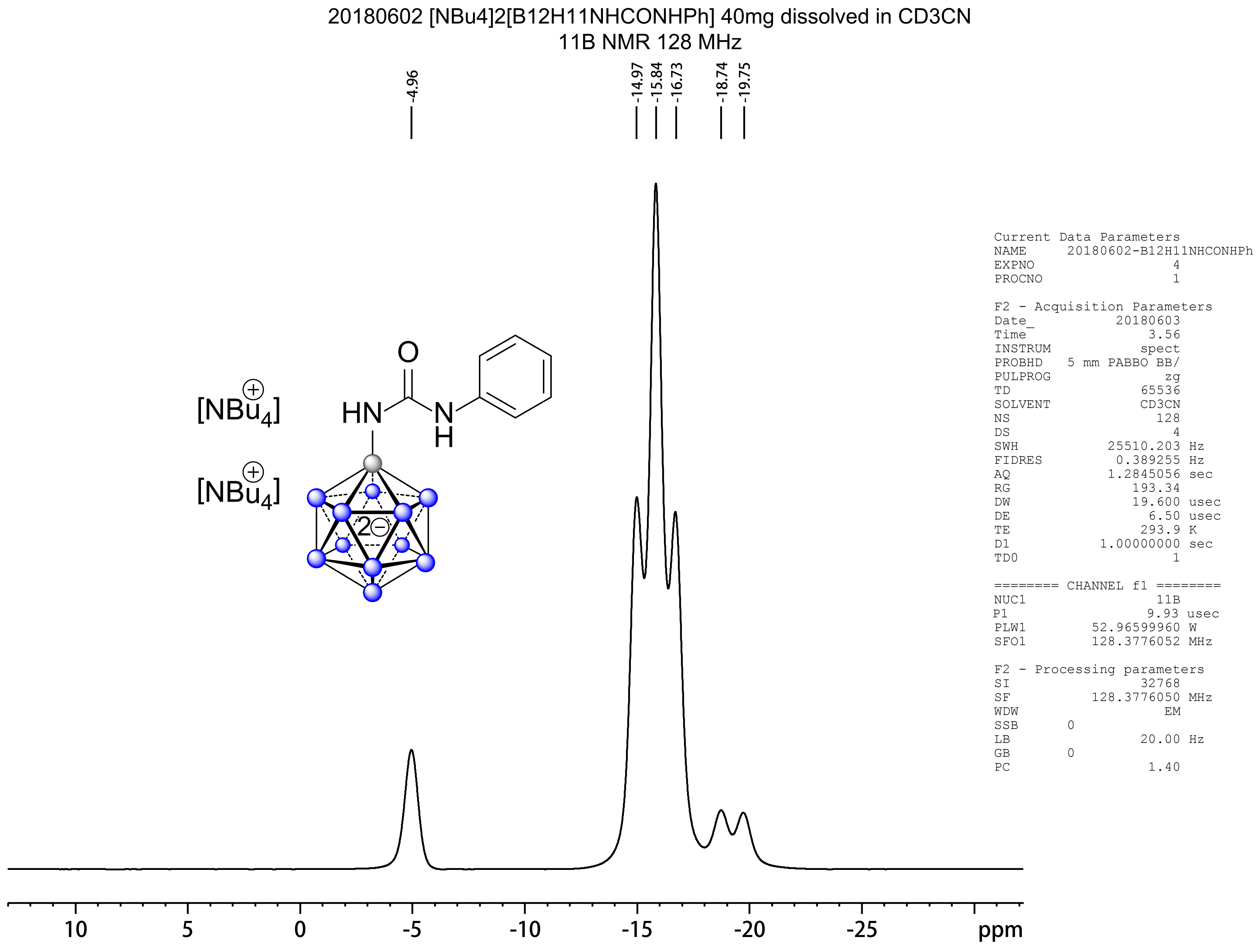
20180602 [NBu4]2[B12H11NHCONHPh] 40mg dissolved in CD3CN
11B NMR 128 MHz
-4.96
-14.97
-15.84
-16.73
-18.74
-19.75
[NBu4]
[NBu4]
HN
O
N
H
2⊖
10
5
0
-5
-10
-15
-20
-25
ppm
Current Data Parameters
NAME 20180602-B12H11NHCONHPh
EXPNO 4
PROCNO 1
F2 - Acquisition Parameters
Date_ 20180603
Time 3.56
INSTRUM spect
PROBHD 5 mm PABBO BB/
PULPROG zg
TD 65536
SOLVENT CD3CN
NS 128
DS 4
SWH 25510.203 Hz
FIDRES 0.389255 Hz
AQ 1.2845056 sec
RG 193.34
DW 19.600 usec
DE 6.50 usec
TE 293.9 K
D1 1.00000000 sec
TD0 1
======== CHANNEL f1 ========
NUC1 11B
P1 9.93 usec
PLW1 52.96599960 W
SFO1 128.3776052 MHz
F2 - Processing parameters
SI 32768
SF 128.3776050 MHz
WDW EM
SSB 0
LB 20.00 Hz
GB 0
PC 1.40

20180602 [NBu4]2[B12H11NHCONHPh] 40mg dissolved in CD3CN
11B{1H} NMR 128 MHz

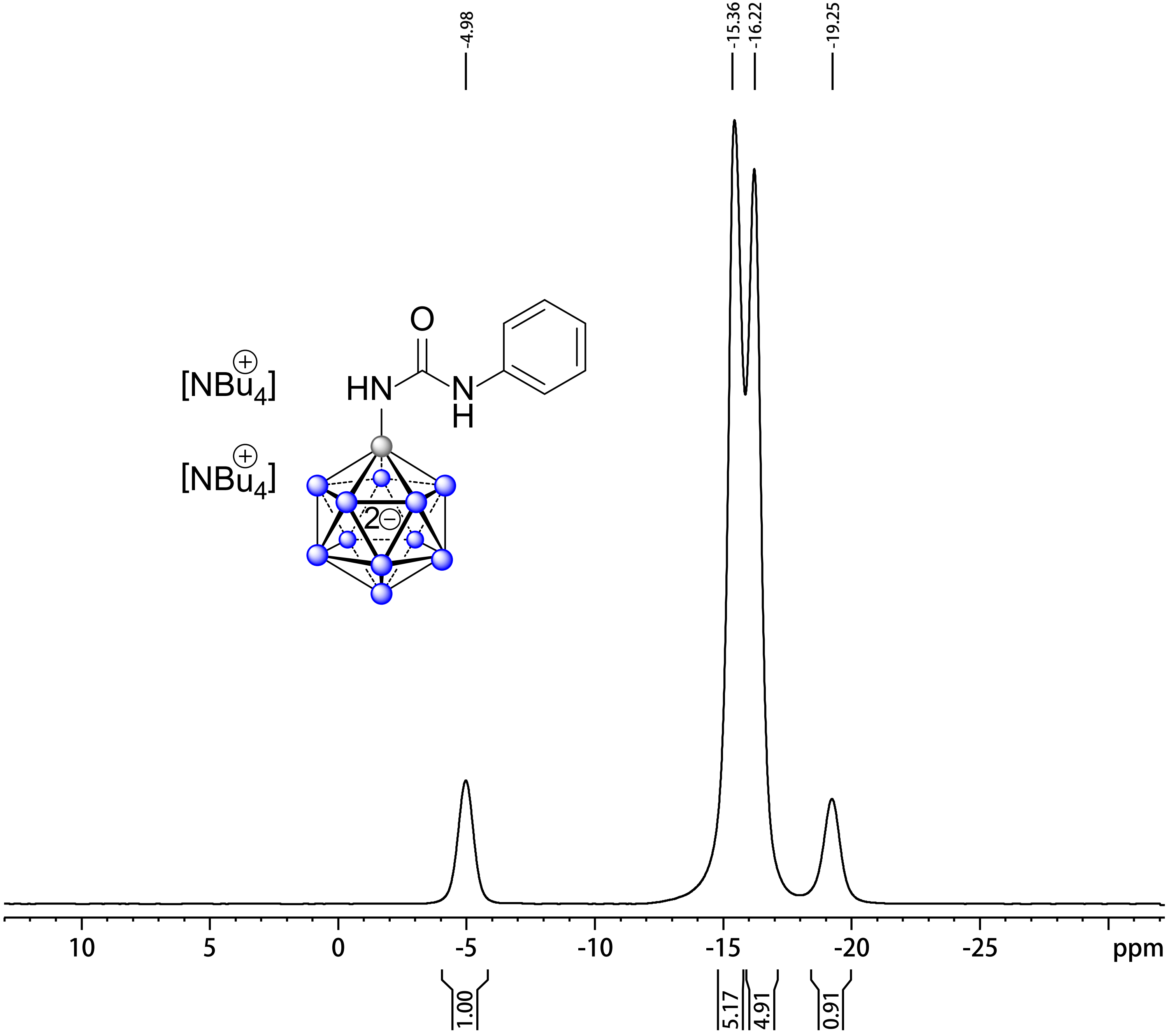


```
Current Data Parameters
NAME     20180602-B12H11NHCONHPh
EXPNO                    3
PROCNO                   1

F2 - Acquisition Parameters
Date_             20180603
Time                  3.50
INSTRUM              spect
PROBHD    5 mm PABBO BB/
PULPROG            zgpg30
TD                   65536
SOLVENT              CD3CN
NS                     128
DS                       4
SWH              25510.203 Hz
FIDRES            0.389255 Hz
AQ               1.2845056 sec
RG                  193.34
DW                  19.600 usec
DE                    6.50 usec
TE                   294.3 K
D1              1.00000000 sec
D11             0.03000000 sec
TD0                      1

======== CHANNEL f1 ========
NUC1                   11B
P1                    9.93 usec
PLW1           52.96599960 W
SFO1           128.3776050 MHz

======== CHANNEL f2 ========
CPDPRG[2           waltz16
NUC2                    1H
PCPD2                80.00 usec
PLW2           12.50000000 W
PLW12           0.43945000 W
PLW13           0.28125000 W
SFO2           400.1320007 MHz

F2 - Processing parameters
SI                   32768
SF             128.3776050 MHz
WDW                     EM
SSB      0
LB                   20.00 Hz
GB       0
PC                    1.40
```

20180603 [PPh4]2[B12H11NHCONHPh] 50mg dissolved in CD3CN
1H{11B} NMR 400 MHz

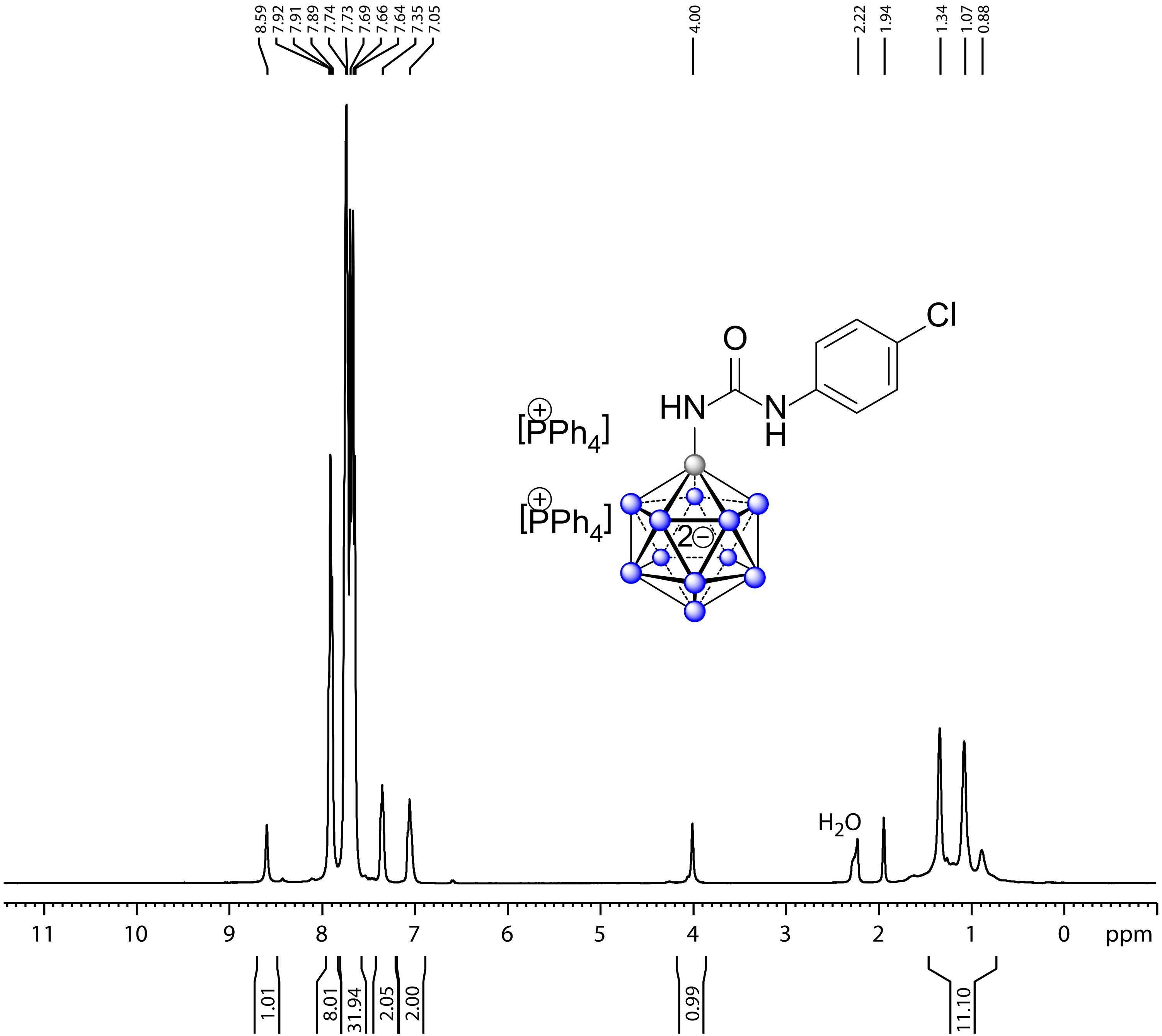


```
Current Data Parameters
NAME      20180604-zyb0704-B12NHCONHPhCl
EXPNO                    2
PROCNO                   1

F2 - Acquisition Parameters
Date_             20180605
Time                  3.33
INSTRUM              spect
PROBHD    5 mm PABBO BB/
PULPROG            zgig30
TD                   16384
SOLVENT              CD3CN
NS                      16
DS                       4
SWH               8012.820 Hz
FIDRES            0.489064 Hz
AQ               1.0223616 sec
RG                   64.43
DW                  62.400 usec
DE                    6.50 usec
TE                   294.4 K
D1              1.00000000 sec
D11             0.03000000 sec
TD0                      1

======== CHANNEL f1 ========
NUC1                    1H
P1                   15.00 usec
PLW1           12.50000000 W
SFO1           400.1320007 MHz

======== CHANNEL f2 ========
CPDPRG[2             garp4
NUC2                   11B
PCPD2                90.00 usec
PLW2           52.96599960 W
PLW12           0.64477998 W
SFO2           128.3776050 MHz

F2 - Processing parameters
SI                   32768
SF             400.1300123 MHz
WDW                     EM
SSB       0
LB                    1.00 Hz
GB        0
PC                    1.40
```

20180603 [PPh4]2[B12H11NHCONHPh] 50mg dissolved in CD3CN
13C{1H} NMR 101MHz

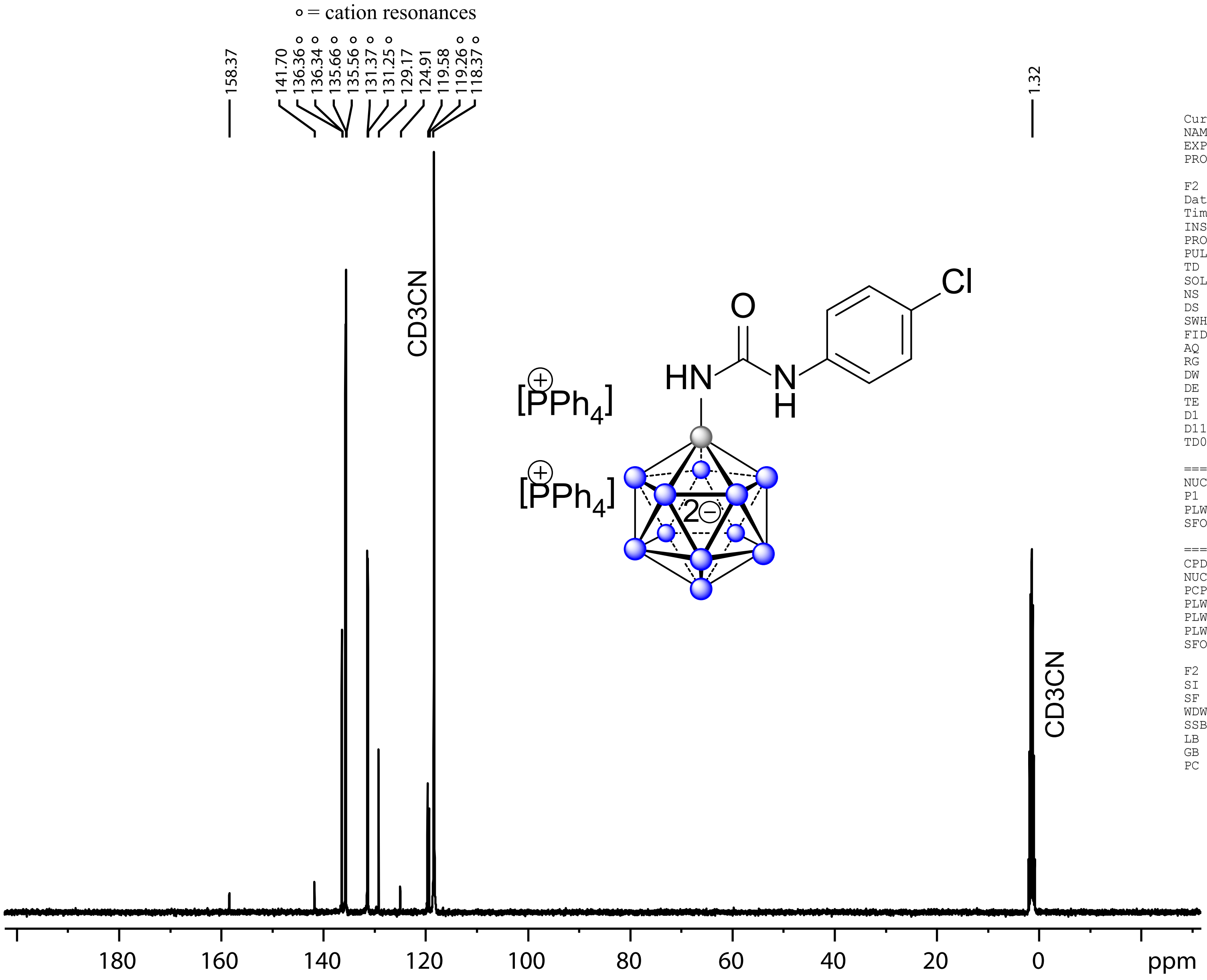


```
Current Data Parameters
NAME      20180604-zyb0704-B12NHCONHPhCl
EXPNO                    5
PROCNO                   1

F2 - Acquisition Parameters
Date_             20180605
Time                  4.09
INSTRUM              spect
PROBHD   5 mm PABBO BB/
PULPROG            zgpg30
TD                   65536
SOLVENT              CD3CN
NS                     512
DS                       4
SWH            29761.904 Hz
FIDRES          0.454131 Hz
AQ             1.1010048 sec
RG                  193.34
DW                  16.800 usec
DE                    6.50 usec
TE                   294.0 K
D1            1.50000000 sec
D11           0.03000000 sec
TD0                      1

======== CHANNEL f1 ========
NUC1                   13C
P1                   10.00 usec
PLW1           53.00000000 W
SFO1           100.6228293 MHz

======== CHANNEL f2 ========
CPDPRG[2           waltz16
NUC2                    1H
PCPD2                80.00 usec
PLW2           12.50000000 W
PLW12           0.43945000 W
PLW13           0.28125000 W
SFO2           400.1316005 MHz

F2 - Processing parameters
SI                   32768
SF             100.6126776 MHz
WDW                     EM
SSB      0
LB                    1.00 Hz
GB       0
PC                    1.40
```

20180603 [PPh4]2[B12H11NHCONHPh] 50mg dissolved in CD3CN
11B NMR 128 MHz

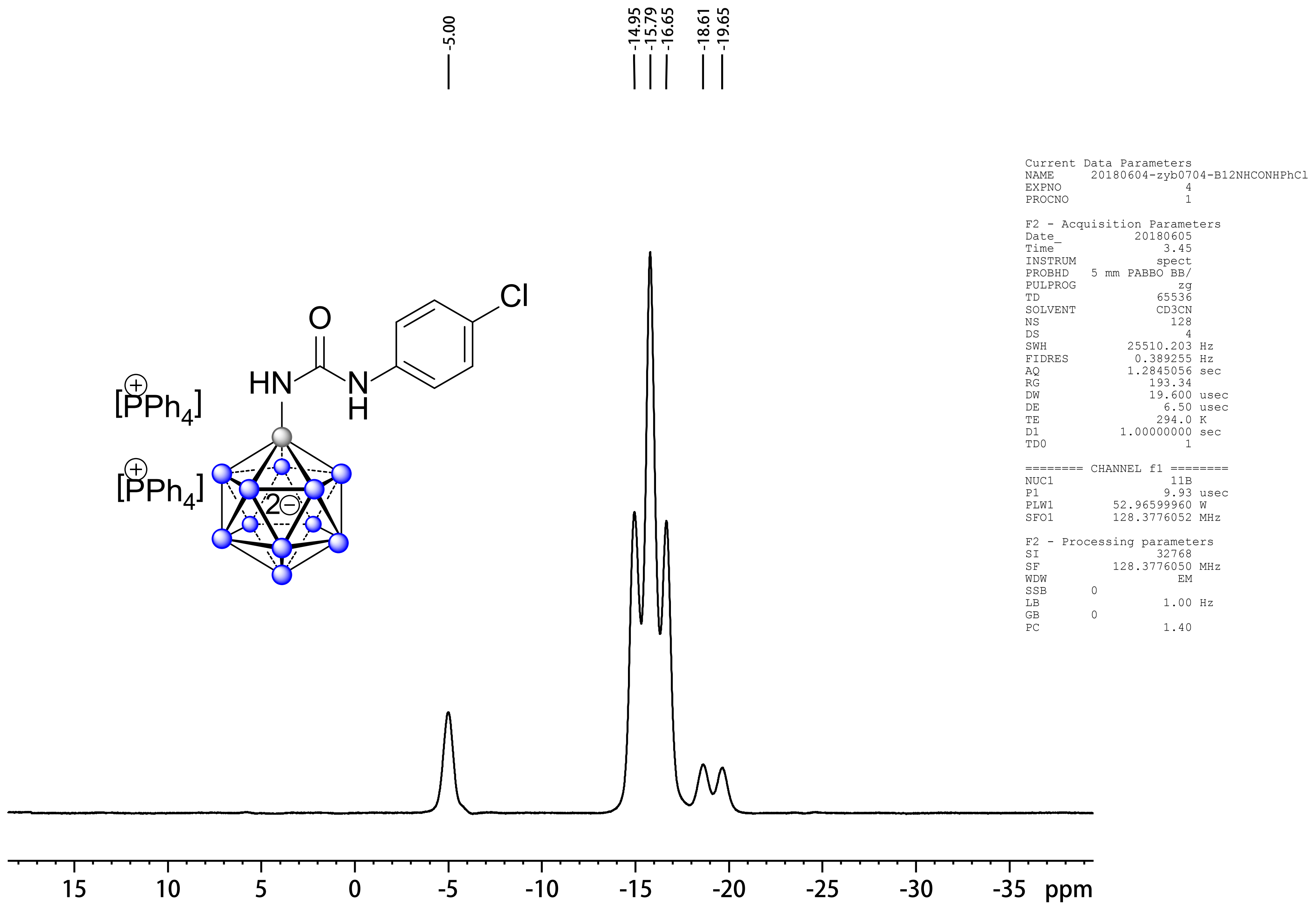

20180603 [PPh4]2[B12H11NHCONHPh] 50mg dissolved in CD3CN
11B{1H} NMR 128 MHz

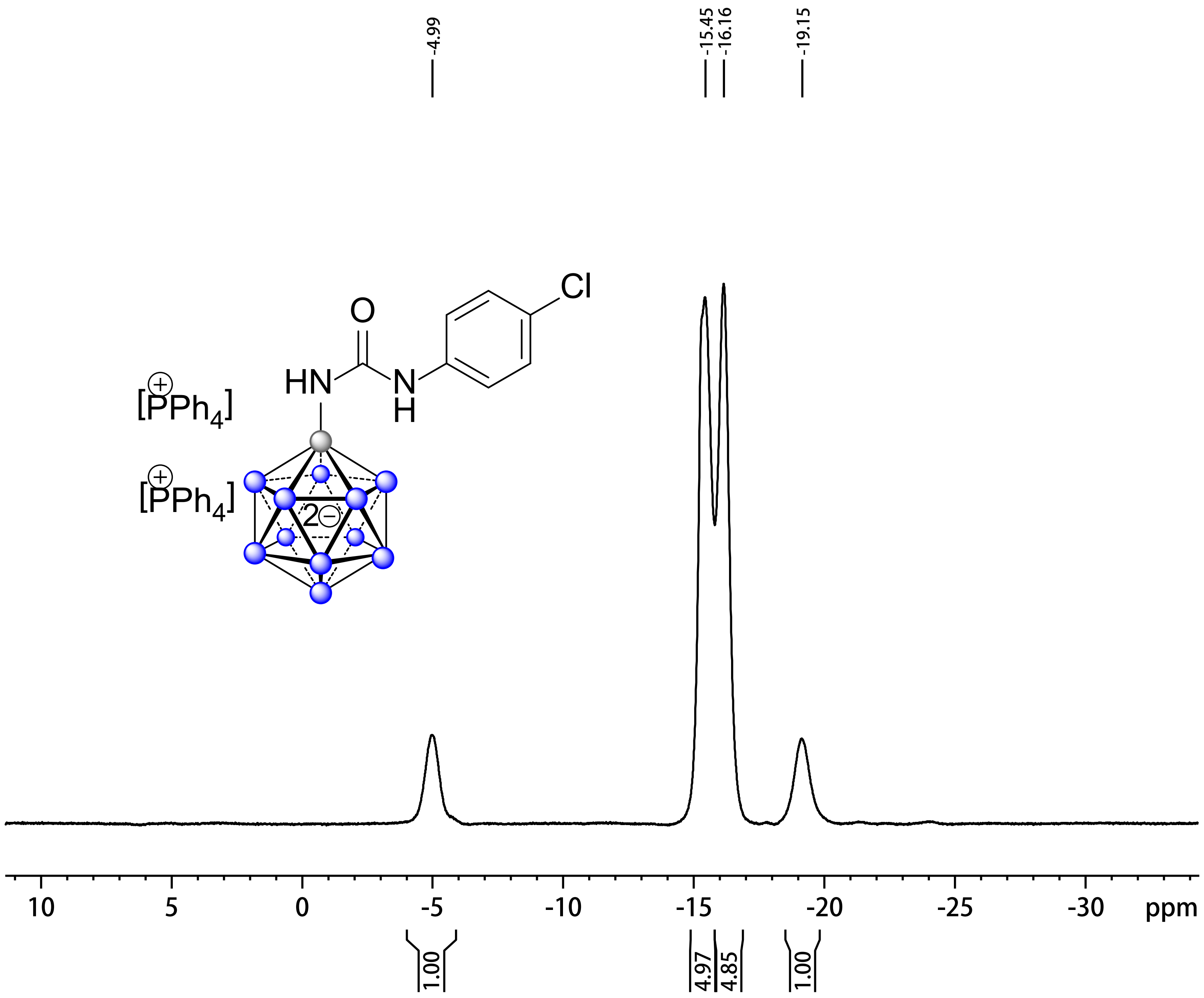


```
Current Data Parameters
NAME     20180604-zyb0704-B12NHCONHPhCl
EXPNO                  3
PROCNO                 1

F2 - Acquisition Parameters
Date_           20180605
Time                3.39
INSTRUM            spect
PROBHD   5 mm PABBO BB/
PULPROG          zgpg30
TD                 65536
SOLVENT            CD3CN
NS                   128
DS                     4
SWH            25510.203 Hz
FIDRES          0.389255 Hz
AQ             1.2845056 sec
RG                193.34
DW                19.600 usec
DE                  6.50 usec
TE                 294.1 K
D1            1.00000000 sec
D11           0.03000000 sec
TD0                    1

======== CHANNEL f1 ========
NUC1                 11B
P1                  9.93 usec
PLW1         52.96599960 W
SFO1         128.3776050 MHz

======== CHANNEL f2 ========
CPDPRG[2         waltz16
NUC2                  1H
PCPD2              80.00 usec
PLW2         12.50000000 W
PLW12         0.43945000 W
PLW13         0.28125000 W
SFO2         400.1320007 MHz

F2 - Processing parameters
SI                 32768
SF           128.3776050 MHz
WDW                   EM
SSB      0
LB                  1.00 Hz
GB       0
PC                  1.40
```

20160629 20 mg [MePPh3]2[B12H11NCO] dissolved in 0.6 mL CD3CN , 1H{11B} NMR, 500MHz

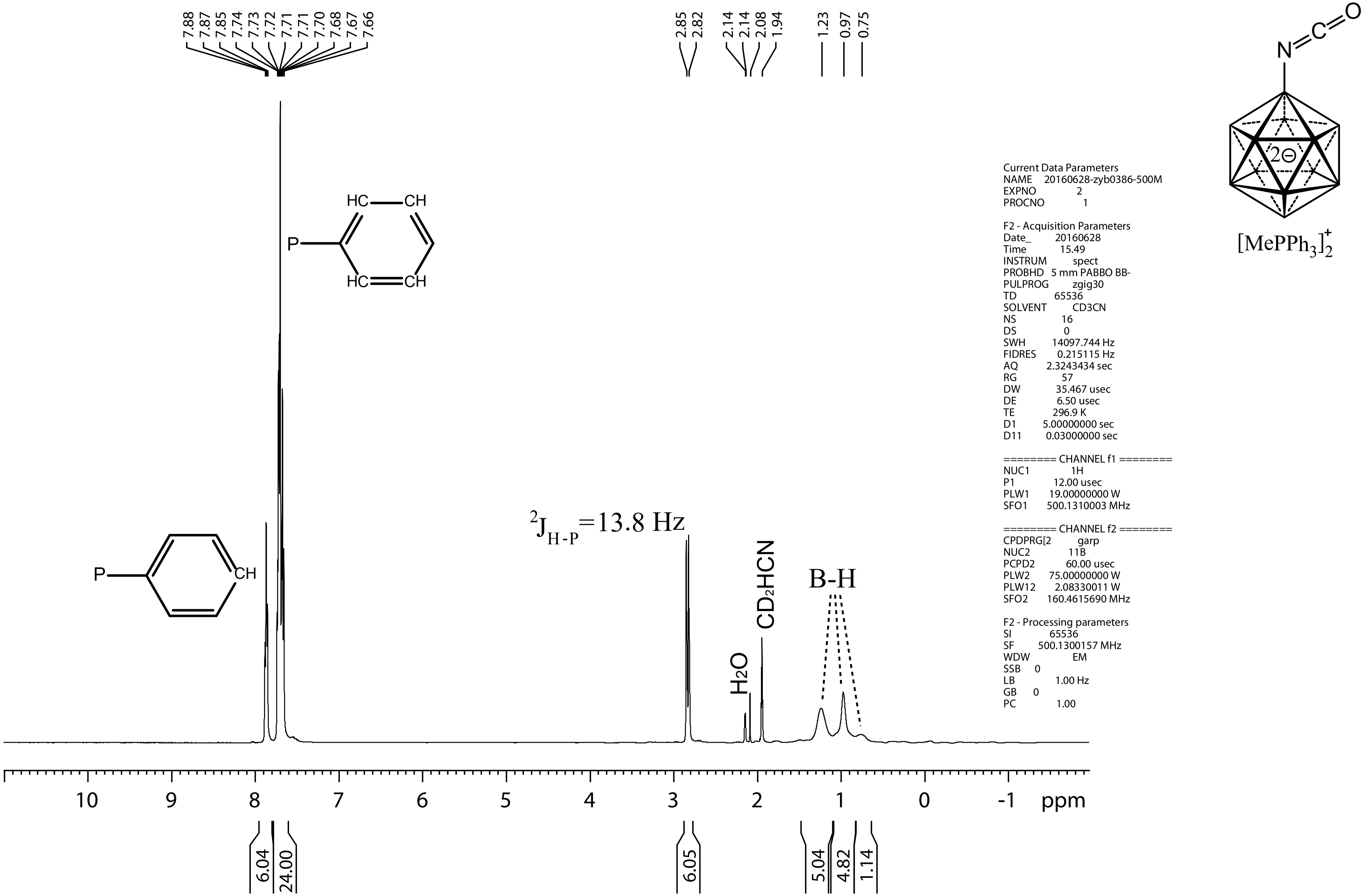

20160629 20 mg [MePPh3]2[B12H11NCO] dissolved in 0.6 mL CD3CN, 1H NMR, 400MHz
signal of NCO not detected

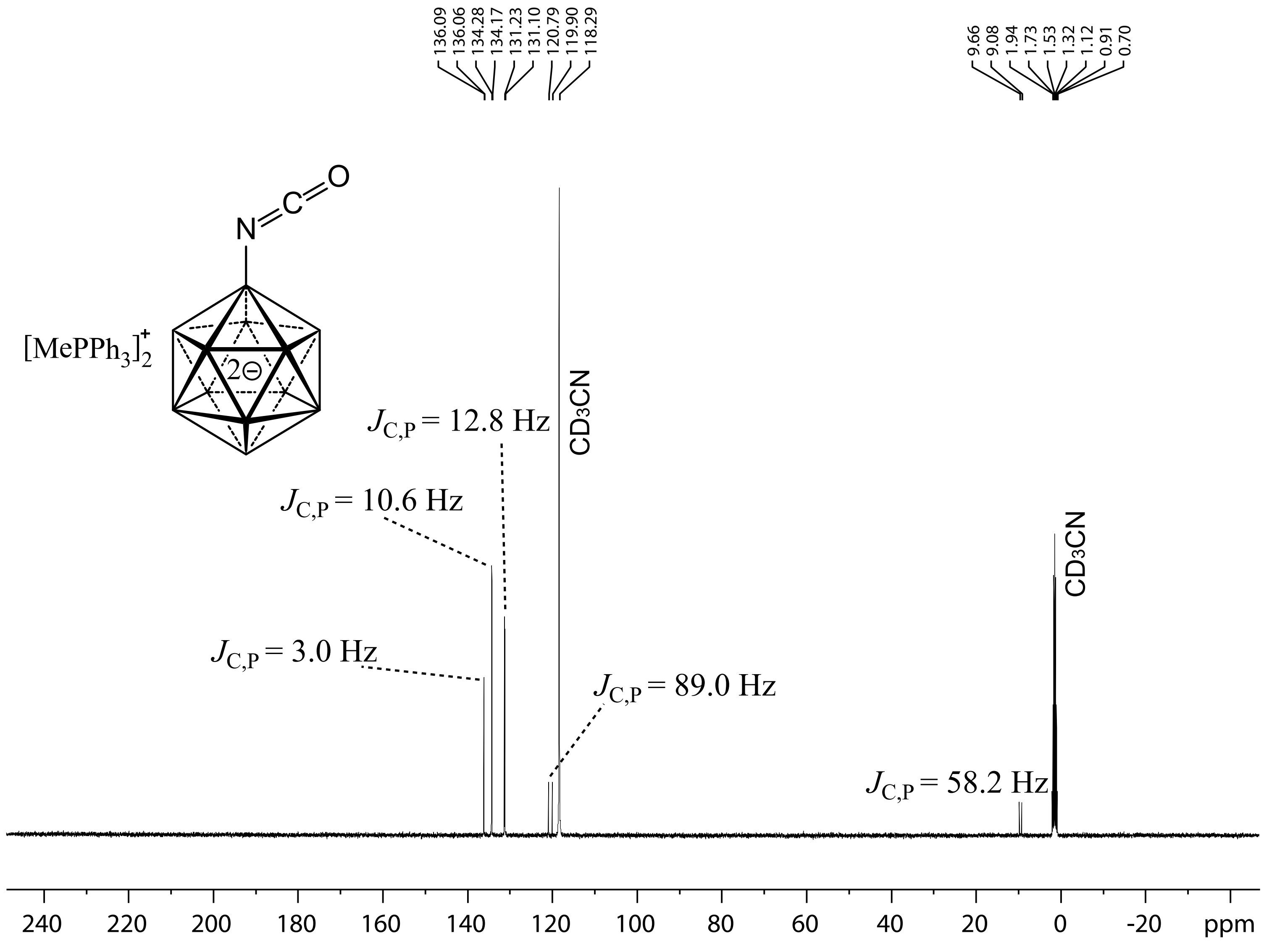

Current Data Parameters
NAME 20160628-zyb0386-CD3CN
EXPNO 2
PROCNO 1

F2 - Acquisition Parameters
Date_ 20160629
Time 9.18
INSTRUM spect
PROBHD 5 mm PABBO BB/
PULPROG zgpg30
TD 65536
SOLVENT CD3CN
NS 512
DS 4
SWH 29761.904 Hz
FIDRES 0.454131 Hz
AQ 1.1010048 sec
RG 193.34
DW 16.800 usec
DE 6.50 usec
TE 296.6 K
D1 1.50000000 sec
D11 0.03000000 sec
TD0 1

======== CHANNEL f1 ========
NUC1 13C
P1 10.00 usec
PLW1 53.00000000 W
SFO1 100.6228293 MHz

======== CHANNEL f2 ========
CPDPRG[2 waltz16
NUC2 1H
PCPD2 80.00 usec
PLW2 12.50000000 W
PLW12 0.43945000 W
PLW13 0.28125000 W
SFO2 400.1316005 MHz

F2 - Processing parameters
SI 32768
SF 100.6126752 MHz
WDW EM
SSB 0
LB 1.00 Hz
GB 0
PC 1.40

20 mg [MePPh3]2[B12H11NCO] dissolved in 0.6 mL CD3CN
11B NMR,160MHz

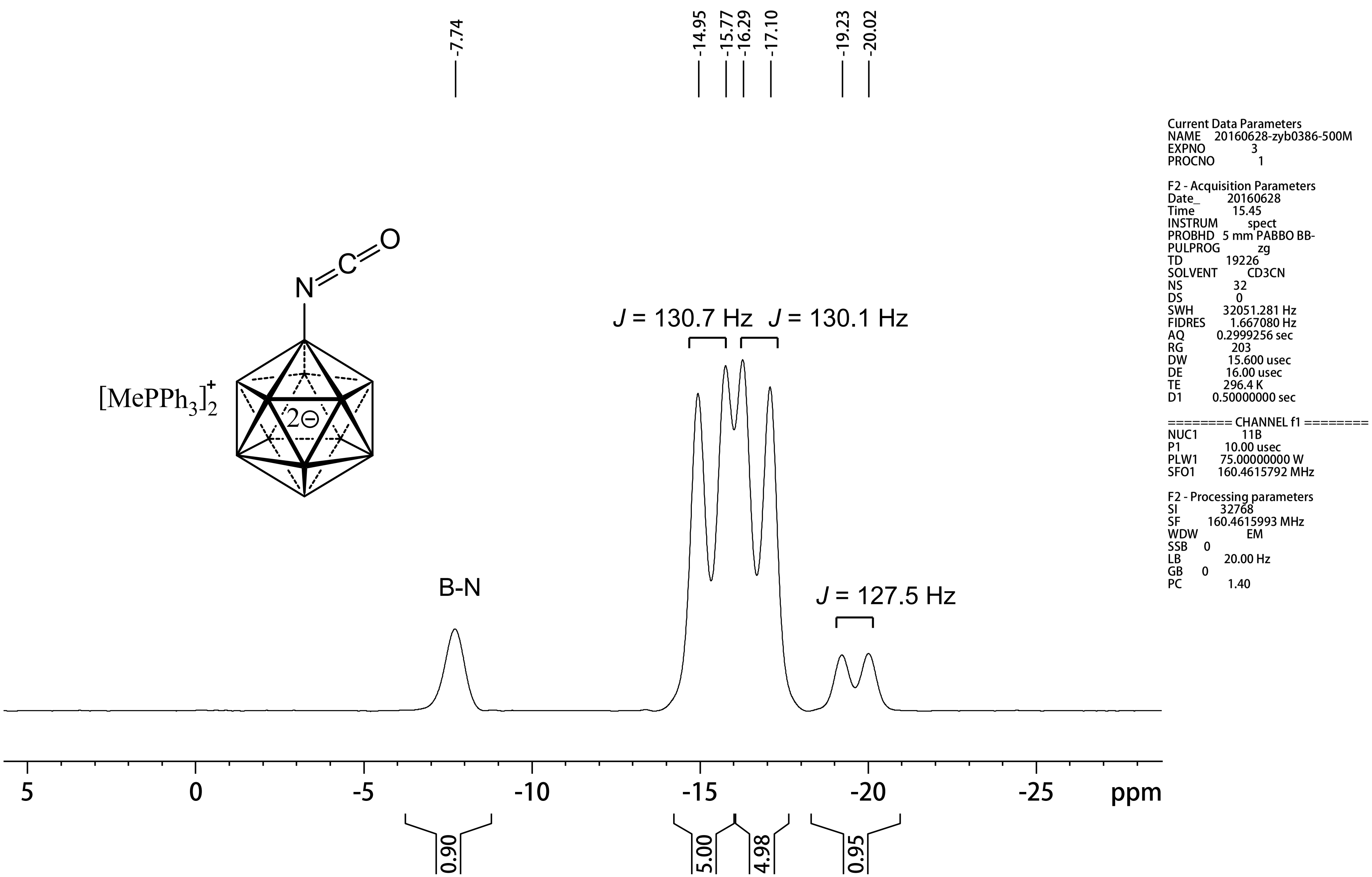

20 mg [MePPh3]2[B12H11NCO] dissolved in 0.6 mL CD3CN
11B{1H} NMR,160MHz

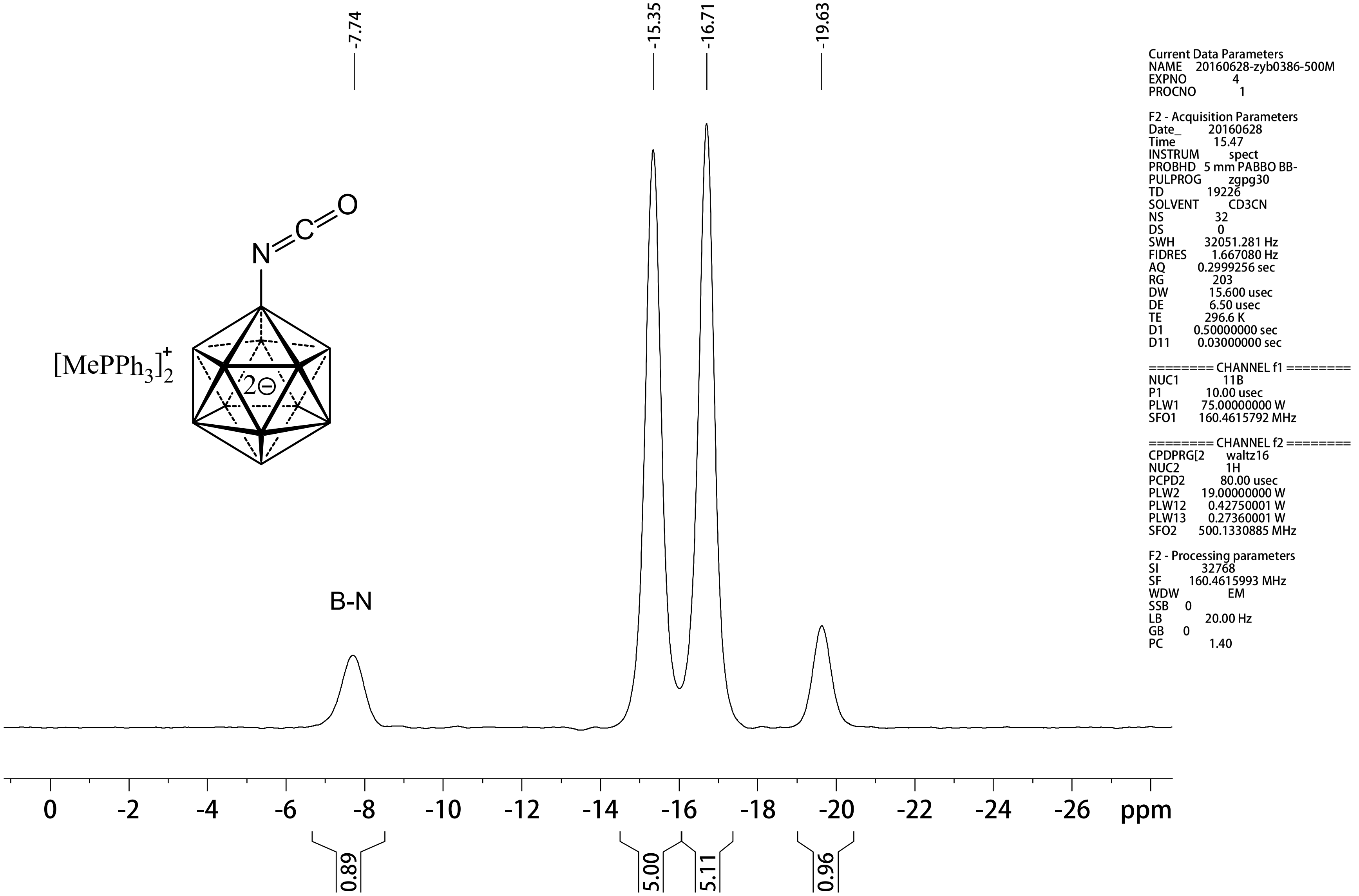

20150313-syj-0077-2, Et3NHB12H11NHCHNMe2
20150313, 400 MHz, 1H{11B}, 12.4mg in 0.6ml CD3CN*

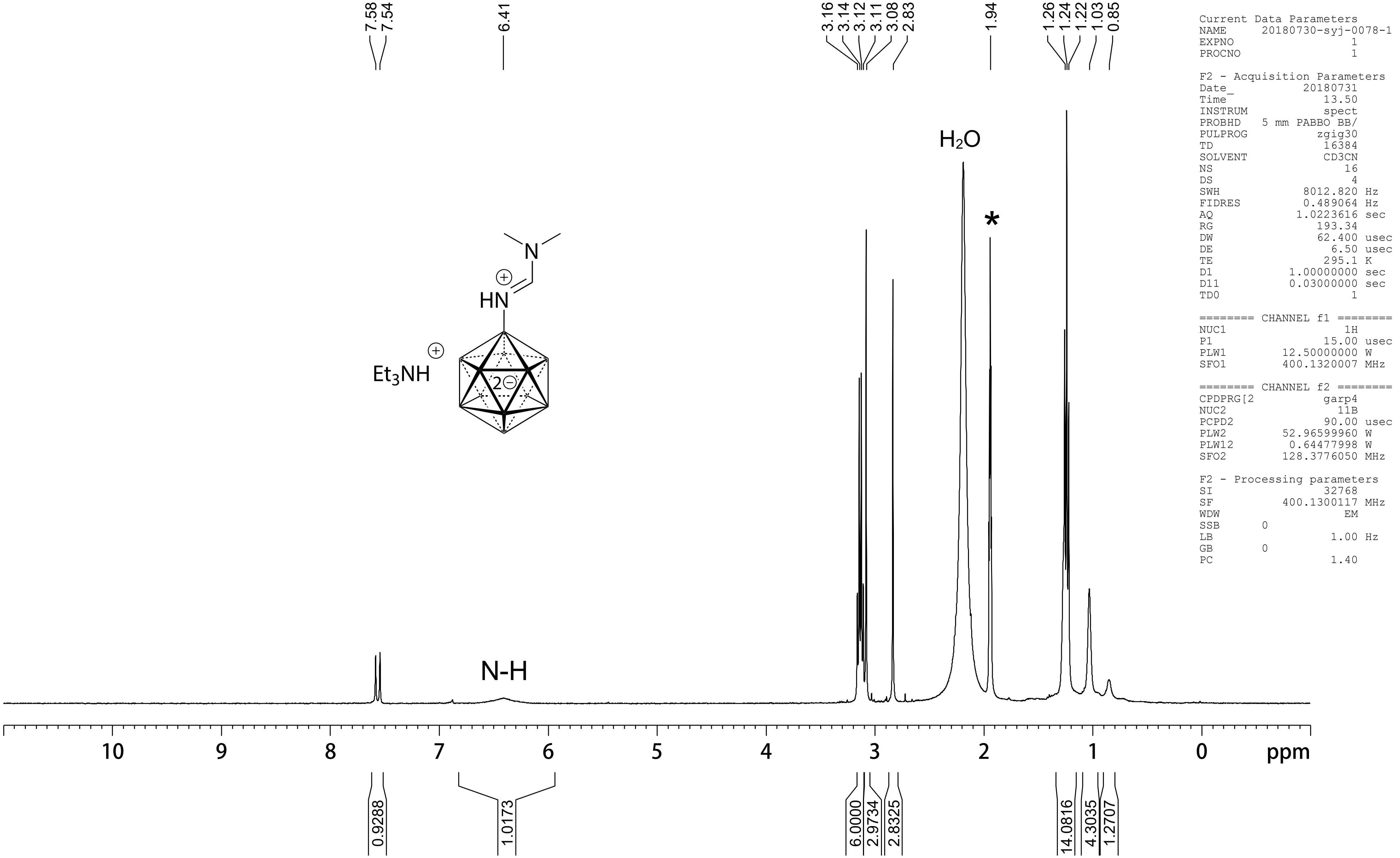

20150313-syj-0077-2, Et3NHB12H11NHCHNMe2
20150313, 100 MHz, 13{1H}, 12.4mg in 0.6ml CD3CN*

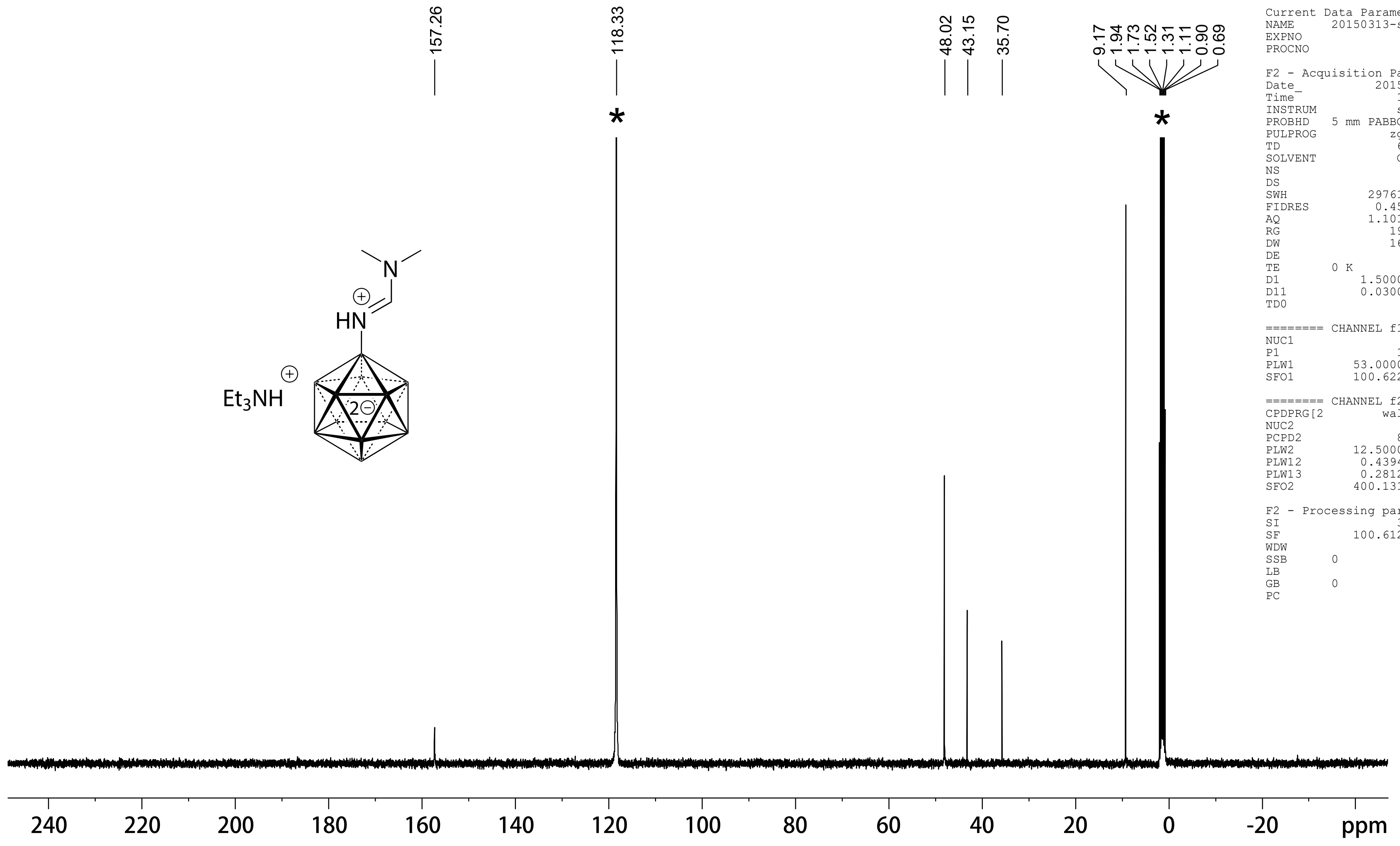


```
Current Data Parameters
NAME     20150313-syj-0078-1
EXPNO                      2
PROCNO                     1

F2 - Acquisition Parameters
Date_           20150314
Time               12.51
INSTRUM            spect
PROBHD   5 mm PABBO BB/
PULPROG          zgpg30
TD                65536
SOLVENT           CD3CN
NS                 1024
DS                    4
SWH           29761.904 Hz
FIDRES         0.454131 Hz
AQ            1.1010048 sec
RG               193.34
DW               16.800 usec
DE                 6.50 usec
TE         0 K
D1           1.50000000 sec
D11          0.03000000 sec
TD0                   1

======== CHANNEL f1 ========
NUC1                 13C
P1                 10.00 usec
PLW1         53.00000000 W
SFO1        100.6228293 MHz

======== CHANNEL f2 ========
CPDPRG[2         waltz16
NUC2                  1H
PCPD2              80.00 usec
PLW2         12.50000000 W
PLW12         0.43945000 W
PLW13         0.28125000 W
SFO2        400.1316005 MHz

F2 - Processing parameters
SI                 32768
SF           100.6126766 MHz
WDW                   EM
SSB       0
LB                  1.00 Hz
GB        0
PC                  1.40
```

20150313-syj-0077-2, Et3NHB12H11NHCHNMe2
20150316, 160 MHz, 11B, 12.4mg in 0.6ml CD3CN

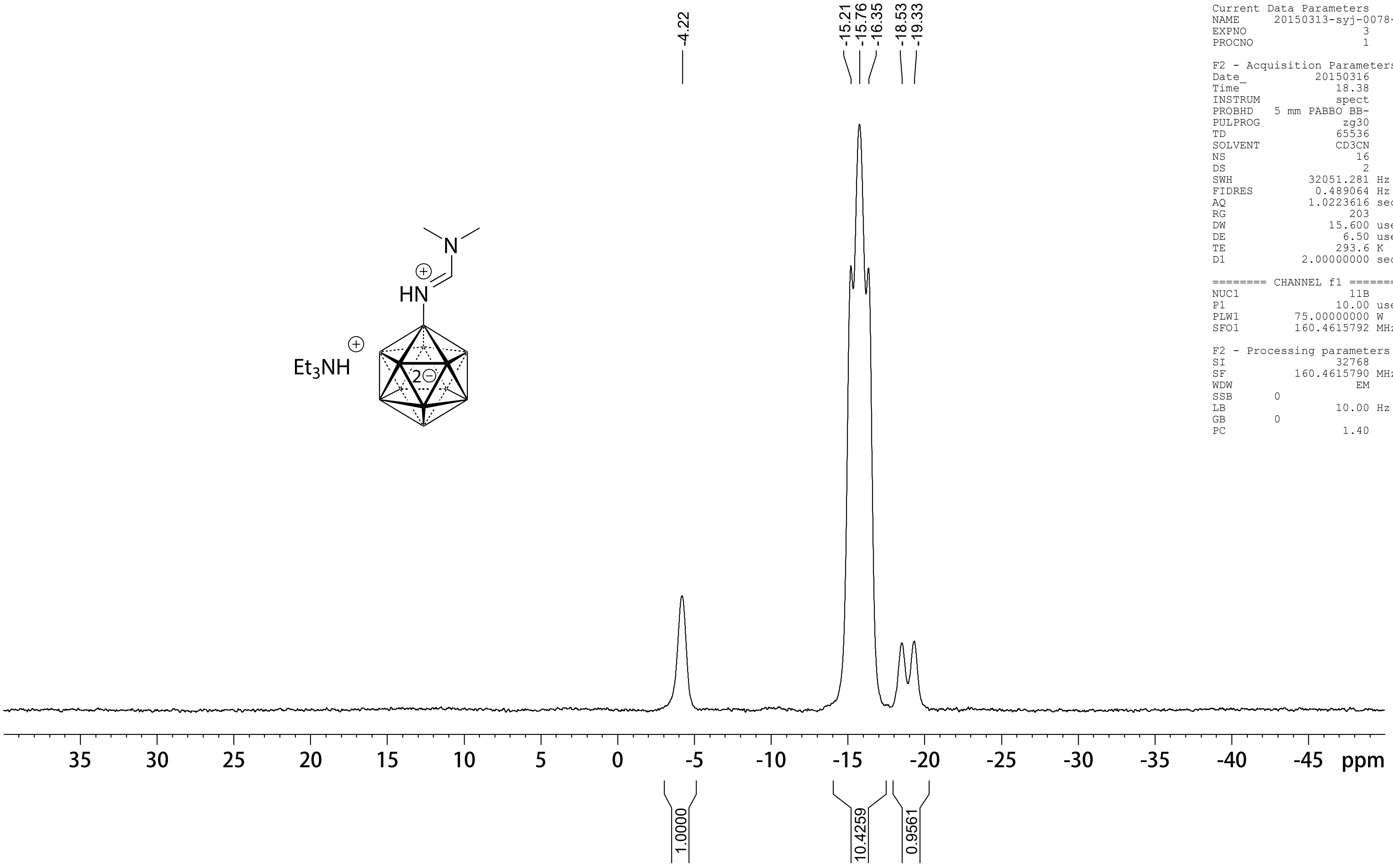


```
Current Data Parameters
NAME     20150313-syj-0078-1
EXPNO                      3
PROCNO                     1

F2 - Acquisition Parameters
Date_               20150316
Time                   18.38
INSTRUM                spect
PROBHD   5 mm PABBO BB-
PULPROG                 zg30
TD                     65536
SOLVENT                CD3CN
NS                        16
DS                         2
SWH                32051.281 Hz
FIDRES              0.489064 Hz
AQ                 1.0223616 sec
RG                       203
DW                    15.600 usec
DE                      6.50 usec
TE                     293.6 K
D1                2.00000000 sec

======== CHANNEL f1 ========
NUC1                     11B
P1                     10.00 usec
PLW1             75.00000000 W
SFO1             160.4615792 MHz

F2 - Processing parameters
SI                     32768
SF               160.4615790 MHz
WDW                       EM
SSB      0
LB                     10.00 Hz
GB       0
PC                      1.40
```

20150313-syj-0077-2, Et3NHB12H11NHCHNMe2
20150316, 160 MHz, 11B{1H}, 12.4mg in 0.6ml CD3CN

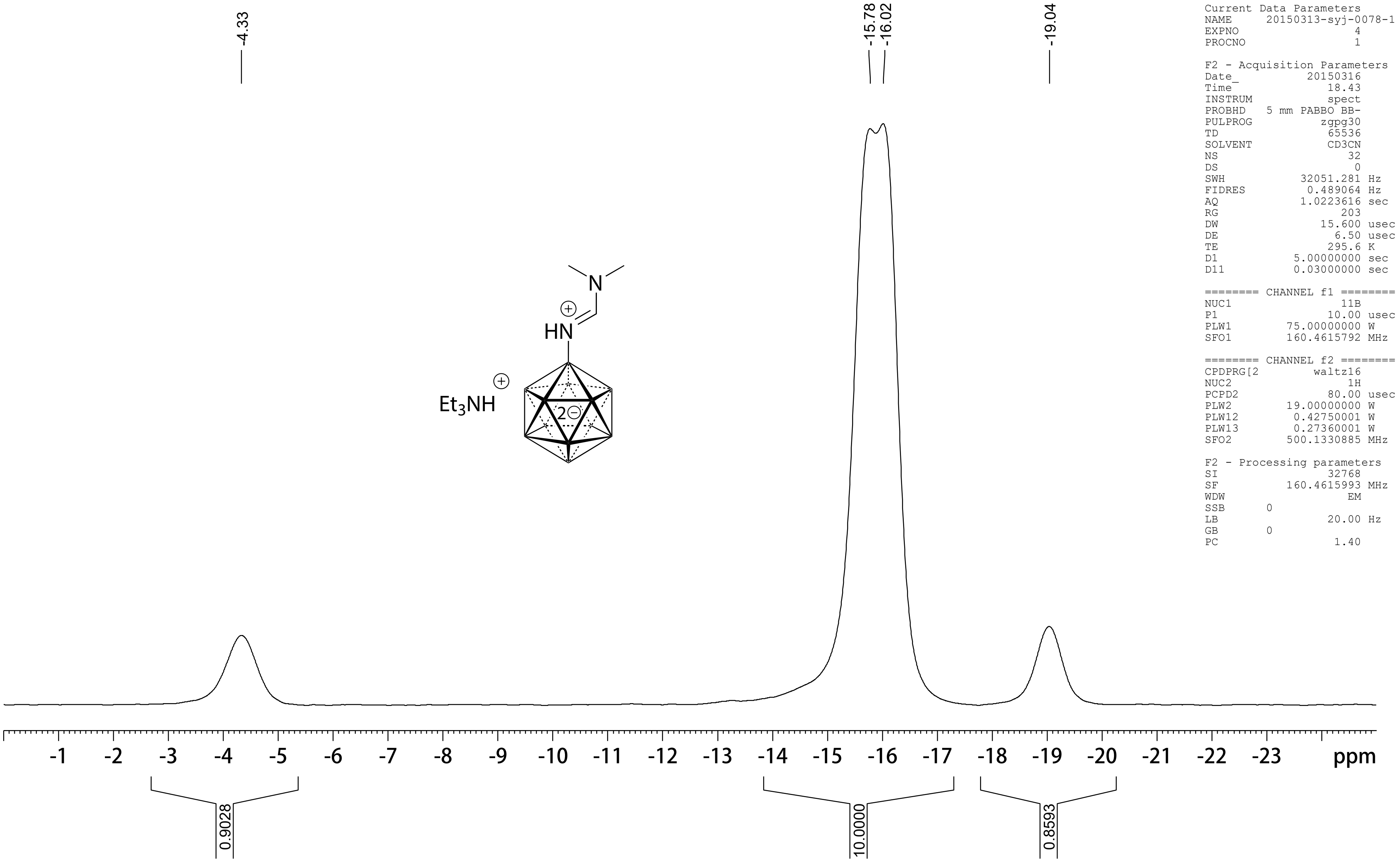

20160228-syj-0196-1, Et3NHB12NHC(C6H5)NHC6H5
20160228, 400 MHz, 1H{11B} NMR, 6.2 mg in 0.6 ml CD2Cl2*

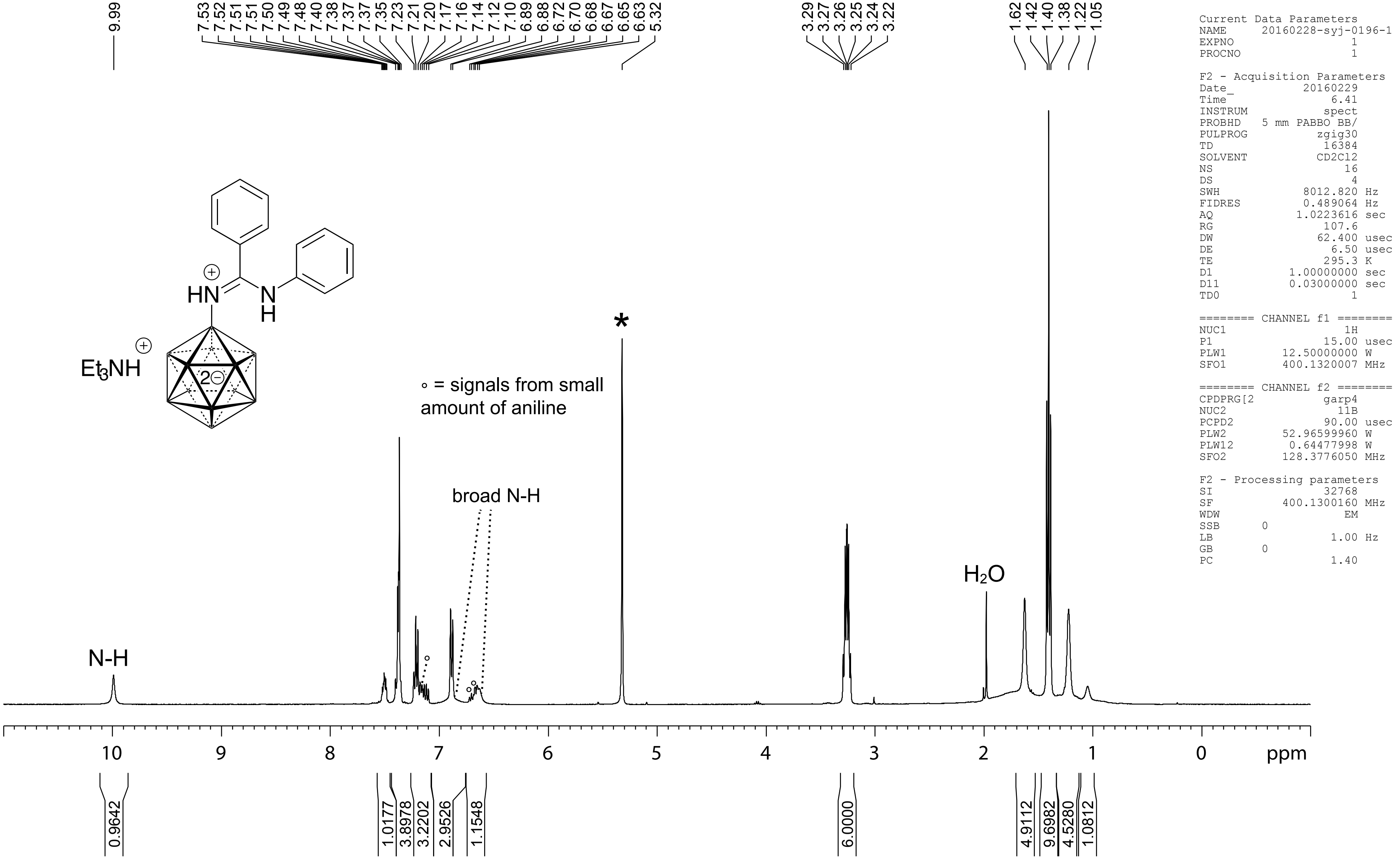

20160228-syj-0196-1, Et3NHB12NHC(C6H5)NHC6H5
20160228, 100 MHz, 13C{1H} NMR, in 0.6 ml CD3CN*

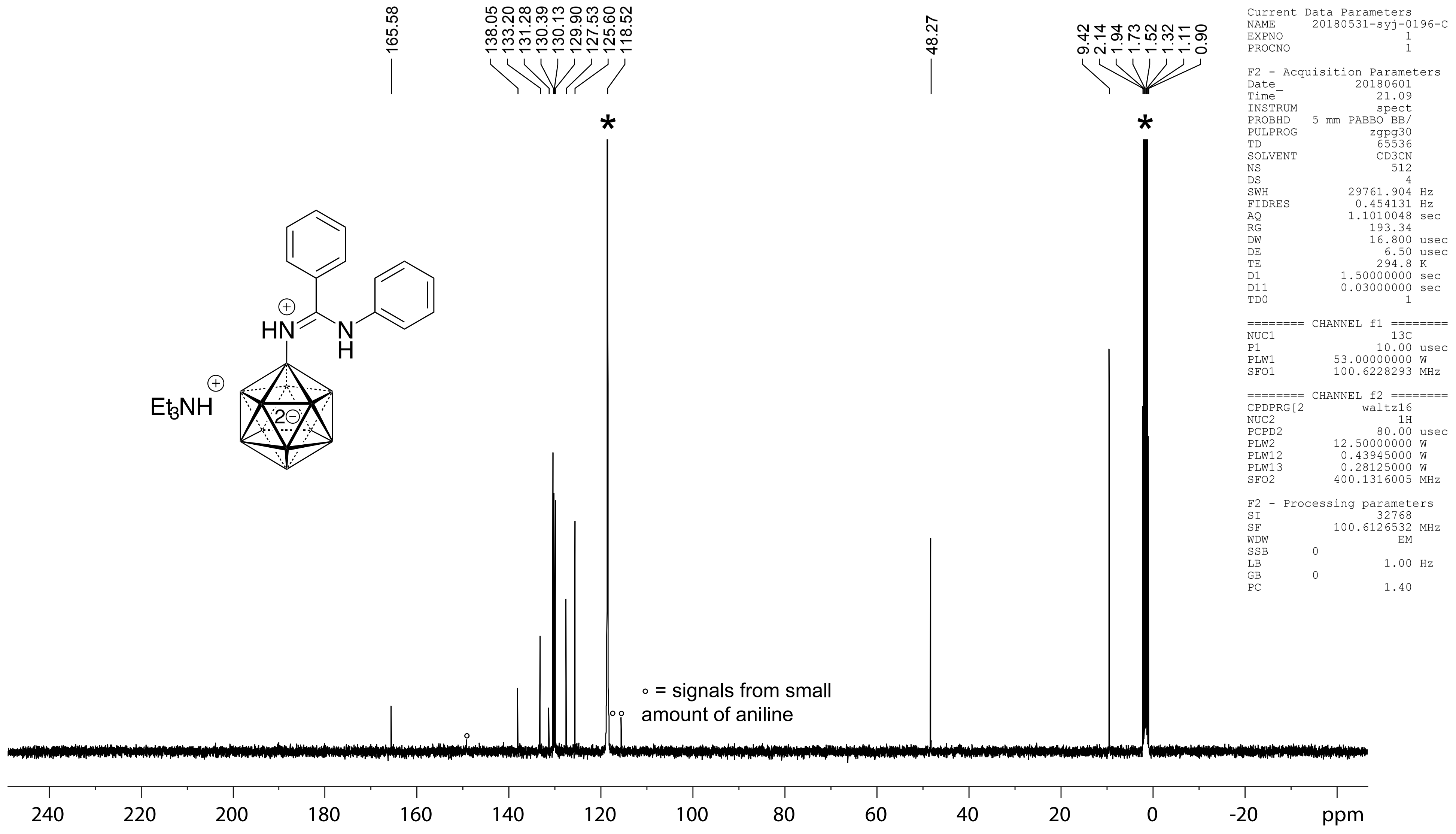

20160228-syj-0196-1, Et3NHB12NHC(C6H5)NHC6H5
20160228, 128 MHz, 11B NMR, 6.2 mg in 0.6 ml CD2Cl2

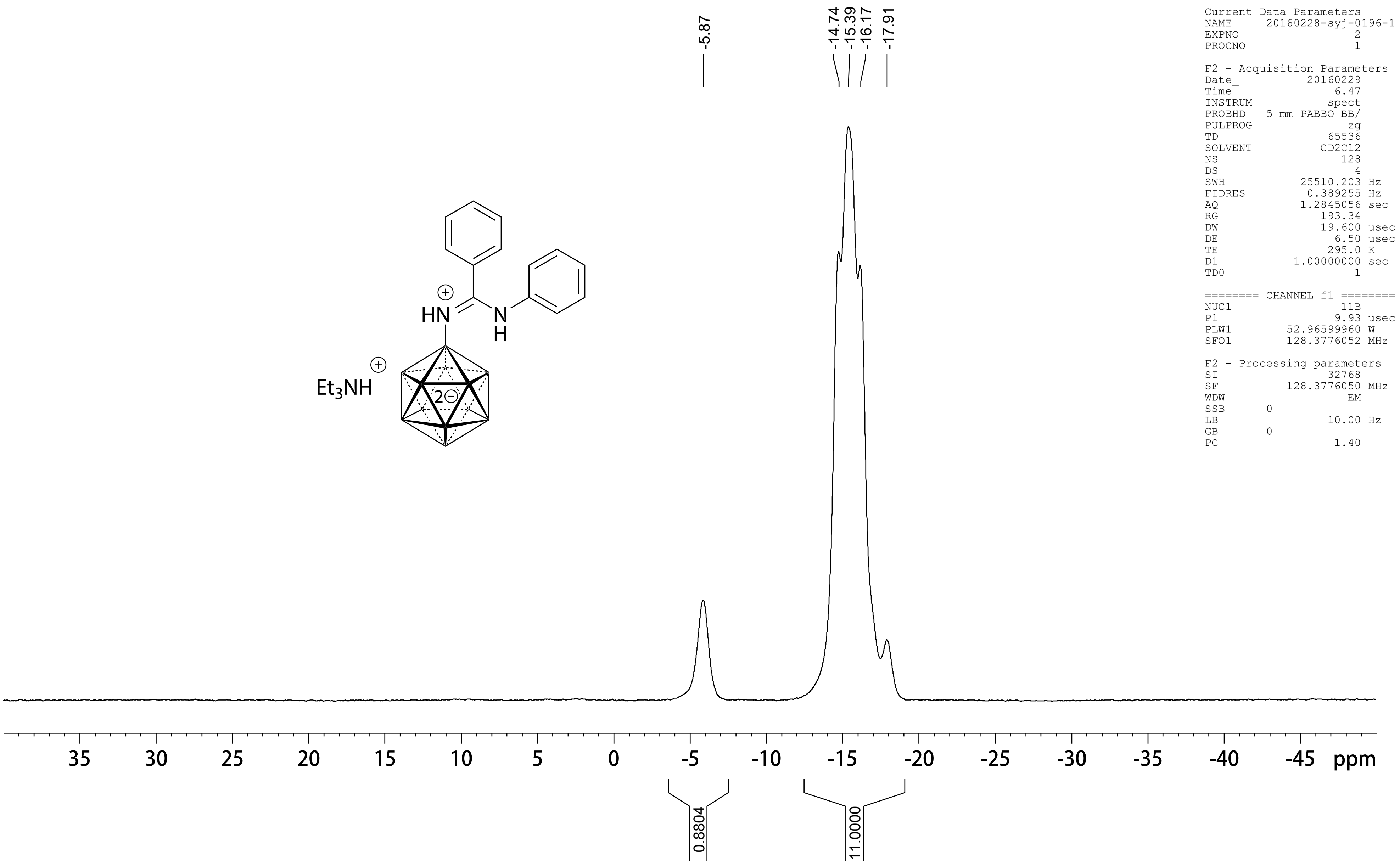

20160228-syj-0196-1, Et3NHB12NHC(C6H5)NHC6H5
20160228, 128 MHz, 11B{1H} NMR, 6.2 mg in 0.6 ml CD2Cl2

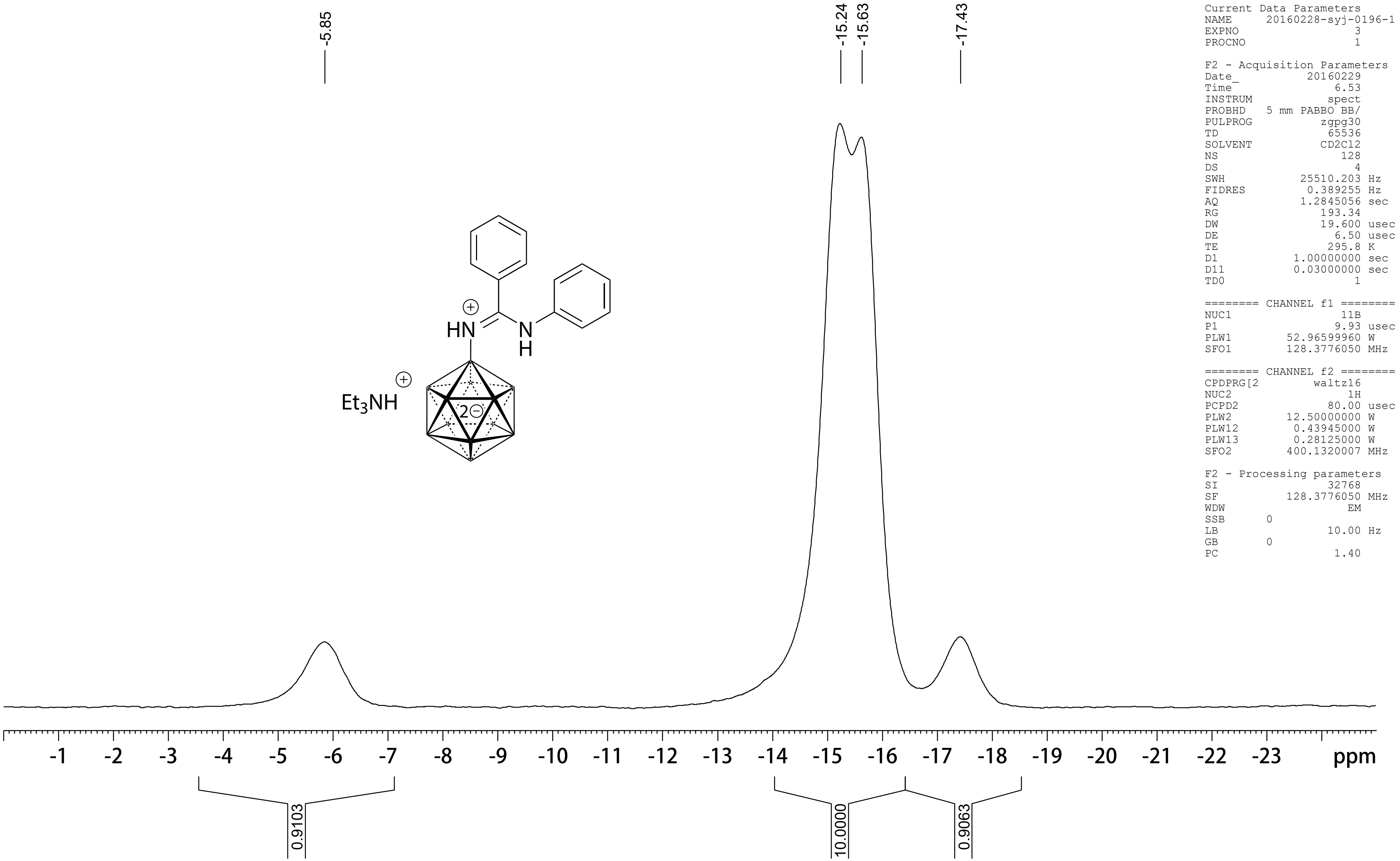

20160223-syj-0193-1, B12NHC(C6H3Cl2)NH(CH2)2NH(CH3)2
20160223, 400 MHz, 1H{11B} NMR, in 0.6 ml CD3CN*

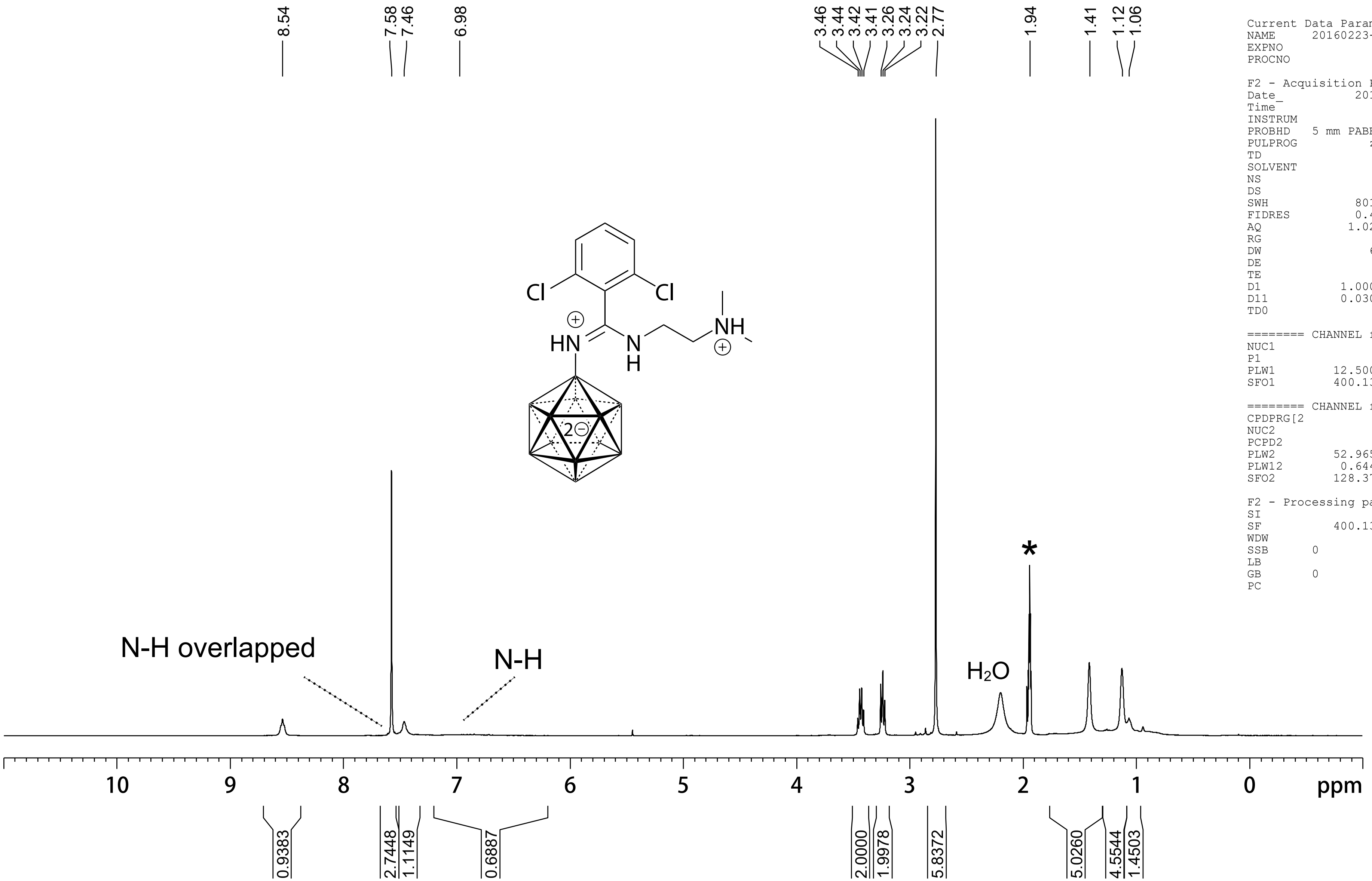


```
Current Data Parameters
NAME     20160223-syj-0193-1
EXPNO                  1
PROCNO                 1

F2 - Acquisition Parameters
Date_            20160224
Time                 1.52
INSTRUM             spect
PROBHD   5 mm PABBO BB/
PULPROG            zgig30
TD                  16384
SOLVENT             CD3CN
NS                     16
DS                      4
SWH              8012.820 Hz
FIDRES           0.489064 Hz
AQ              1.0223616 sec
RG                  107.6
DW                 62.400 usec
DE                   6.50 usec
TE                  291.3 K
D1             1.00000000 sec
D11            0.03000000 sec
TD0                     1

======== CHANNEL f1 ========
NUC1                   1H
P1                  15.00 usec
PLW1          12.50000000 W
SFO1          400.1320007 MHz

======== CHANNEL f2 ========
CPDPRG[2            garp4
NUC2                  11B
PCPD2               90.00 usec
PLW2          52.96599960 W
PLW12          0.64477998 W
SFO2          128.3776050 MHz

F2 - Processing parameters
SI                  32768
SF            400.1300119 MHz
WDW                    EM
SSB      0
LB                   1.00 Hz
GB       0
PC                   1.40
```

20160223-syj-0193-1, B12NHC(C6H3Cl2)NH(CH2)2NH(CH3)2
20160223, 100 MHz, 13C{1H} NMR, in 0.6 ml CD3CN*

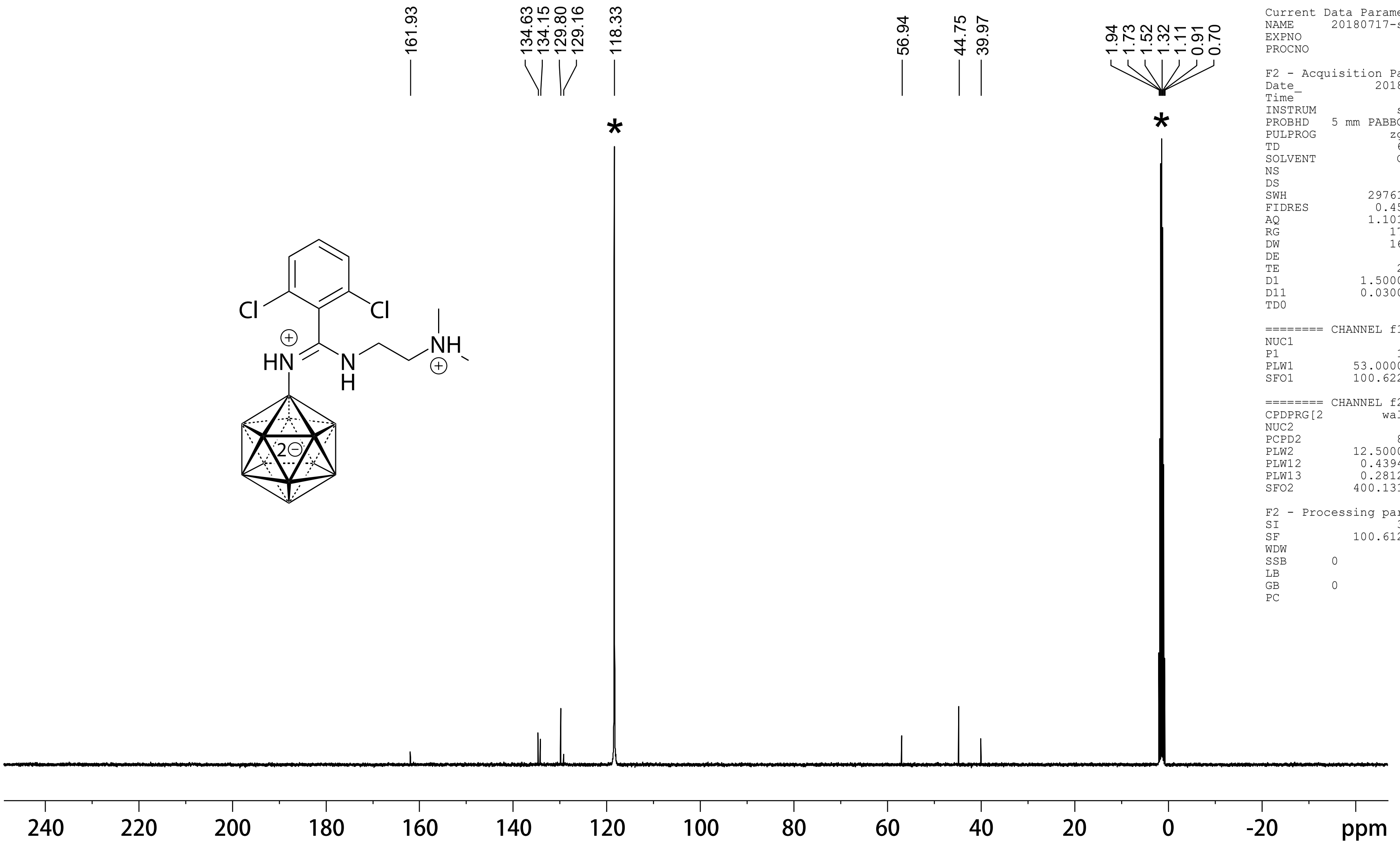


```
Current Data Parameters
NAME       20180717-syj-0193-C
EXPNO                    1
PROCNO                   1

F2 - Acquisition Parameters
Date_             20180718
Time                  0.24
INSTRUM              spect
PROBHD   5 mm PABBO BB/
PULPROG             zgpg30
TD                   65536
SOLVENT              CD3CN
NS                     512
DS                       4
SWH            29761.904 Hz
FIDRES          0.454131 Hz
AQ             1.1010048 sec
RG                  170.36
DW                  16.800 usec
DE                    6.50 usec
TE                   295.4 K
D1              1.50000000 sec
D11             0.03000000 sec
TD0                      1

======== CHANNEL f1 ========
NUC1                   13C
P1                   10.00 usec
PLW1           53.00000000 W
SFO1           100.6228293 MHz

======== CHANNEL f2 ========
CPDPRG[2           waltz16
NUC2                    1H
PCPD2                80.00 usec
PLW2           12.50000000 W
PLW12           0.43945000 W
PLW13           0.28125000 W
SFO2           400.1316005 MHz

F2 - Processing parameters
SI                   32768
SF             100.6126732 MHz
WDW                     EM
SSB      0
LB                    1.00 Hz
GB       0
PC                    1.40
```

20160223-syj-0193-1, B12NHC(C6H3Cl2)NH(CH2)2NH(CH3)2
20160223, 128 MHz, 11B NMR, in 0.6 ml CD3CN

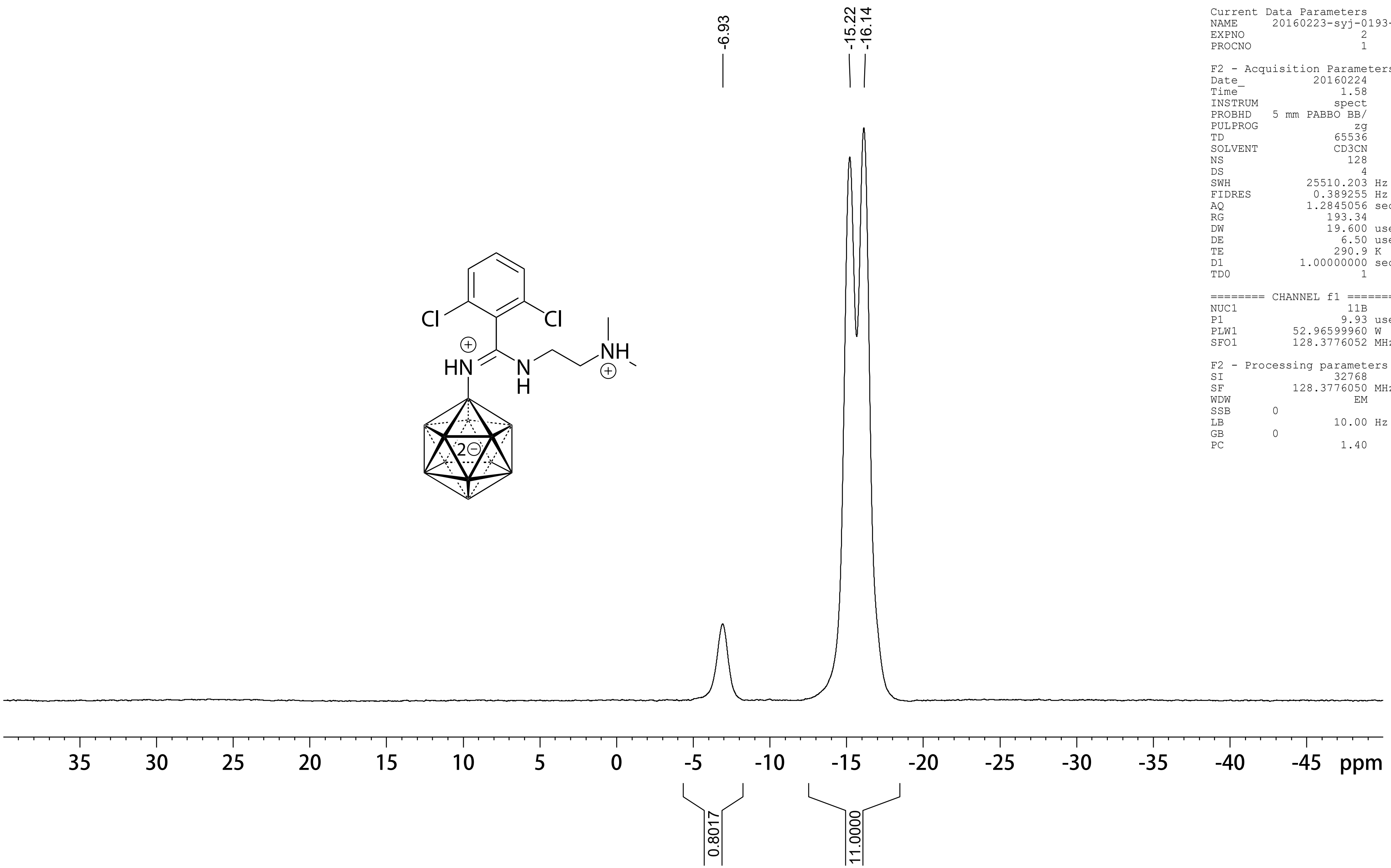


```
Current Data Parameters
NAME     20160223-syj-0193-1
EXPNO                      2
PROCNO                     1

F2 - Acquisition Parameters
Date_           20160224
Time                1.58
INSTRUM            spect
PROBHD   5 mm PABBO BB/
PULPROG               zg
TD                 65536
SOLVENT            CD3CN
NS                   128
DS                     4
SWH            25510.203 Hz
FIDRES          0.389255 Hz
AQ             1.2845056 sec
RG                193.34
DW                19.600 usec
DE                  6.50 usec
TE                 290.9 K
D1            1.00000000 sec
TD0                    1

======== CHANNEL f1 ========
NUC1                 11B
P1                  9.93 usec
PLW1         52.96599960 W
SFO1         128.3776052 MHz

F2 - Processing parameters
SI                 32768
SF           128.3776050 MHz
WDW                   EM
SSB      0
LB                 10.00 Hz
GB       0
PC                  1.40
```

20160223-syj-0193-1, B12NHC(C6H3Cl2)NH(CH2)2NH(CH3)2
20160223, 128 MHz, 11B{1H} NMR, in 0.6 ml CD3CN

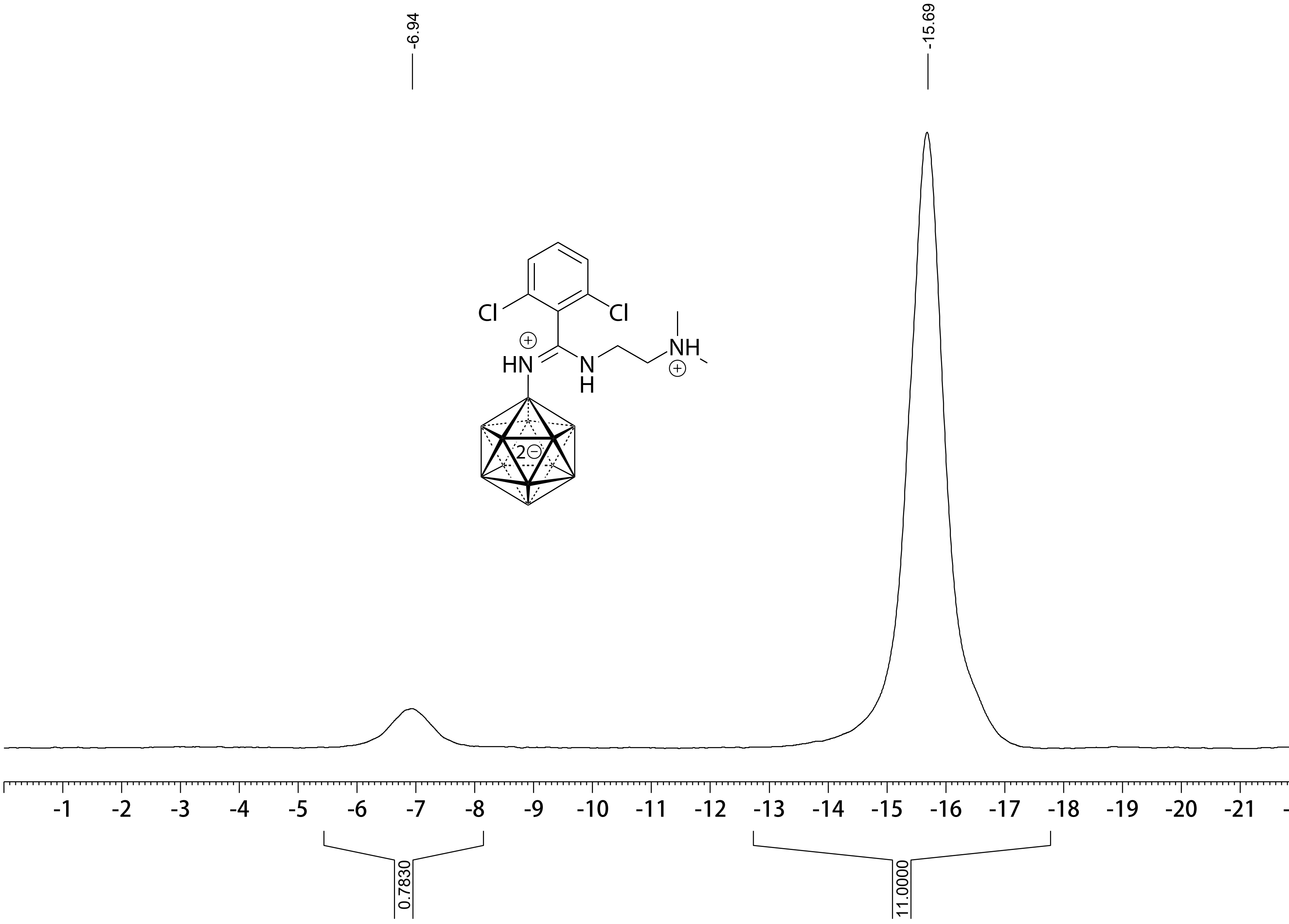


```
Current Data Parameters
NAME     20160223-syj-0193-1
EXPNO                      3
PROCNO                     1

F2 - Acquisition Parameters
Date_           20160224
Time                2.04
INSTRUM            spect
PROBHD   5 mm PABBO BB/
PULPROG          zgpg30
TD                 65536
SOLVENT            CD3CN
NS                   128
DS                     4
SWH            25510.203 Hz
FIDRES          0.389255 Hz
AQ             1.2845056 sec
RG                193.34
DW                19.600 usec
DE                  6.50 usec
TE                 291.7 K
D1            1.00000000 sec
D11           0.03000000 sec
TD0                    1

======== CHANNEL f1 ========
NUC1                 11B
P1                  9.93 usec
PLW1         52.96599960 W
SFO1        128.3776050 MHz

======== CHANNEL f2 ========
CPDPRG[2         waltz16
NUC2                  1H
PCPD2              80.00 usec
PLW2         12.50000000 W
PLW12         0.43945000 W
PLW13         0.28125000 W
SFO2        400.1320007 MHz

F2 - Processing parameters
SI                 32768
SF          128.3776050 MHz
WDW                   EM
SSB      0
LB                 10.00 Hz
GB       0
PC                  1.40
```
